# *Brevicoryne brassicae* aphids interfere with transcriptome responses of *Arabidopsis thaliana* to feeding by *Plutella xylostella* caterpillars in a density-dependent manner

**DOI:** 10.1007/s00442-016-3758-3

**Published:** 2016-10-22

**Authors:** Anneke Kroes, Colette Broekgaarden, Marcos Castellanos Uribe, Sean May, Joop J. A. van Loon, Marcel Dicke

**Affiliations:** 1 Laboratory of Entomology, Wageningen University, P.O. Box 16, 6700 AA Wageningen, The Netherlands; 2 Plant-Microbe Interactions, Department of Biology, Faculty of Science, Utrecht University, 3508 TB Utrecht, The Netherlands; 3 Nottingham Arabidopsis Stock Centre, University of Nottingham, School of Biosciences, Loughborough, LE12 5RD UK

**Keywords:** Feeding guilds, Microarray, Multiple herbivory, Plant defense, Transcriptome

## Abstract

Plants are commonly attacked by multiple herbivorous species. Yet, little is known about transcriptional patterns underlying plant responses to multiple insect attackers feeding simultaneously. Here, we assessed transcriptomic responses of *Arabidopsis thaliana* plants to simultaneous feeding by *Plutella xylostella* caterpillars and *Brevicoryne brassicae* aphids in comparison to plants infested by *P. xylostella* caterpillars alone, using microarray analysis. We particularly investigated how aphid feeding interferes with the transcriptomic response to *P. xylostella* caterpillars and whether this interference is dependent on aphid density and time since aphid attack. Various JA-responsive genes were up-regulated in response to feeding by *P. xylostella* caterpillars. The additional presence of aphids, both at low and high densities, clearly affected the transcriptional plant response to caterpillars. Interestingly, some important modulators of plant defense signalling, including *WRKY* transcription factor genes and ABA-dependent genes, were differentially induced in response to simultaneous aphid feeding at low or high density compared with responses to *P. xylostella* caterpillars feeding alone. Furthermore, aphids affected the *P. xylostella*-induced transcriptomic response in a density-dependent manner, which caused an acceleration in plant response against dual insect attack at high aphid density compared to dual insect attack at low aphid density. In conclusion, our study provides evidence that aphids influence the caterpillar-induced transcriptional response of *A. thaliana* in a density-dependent manner. It highlights the importance of addressing insect density to understand how plant responses to single attackers interfere with responses to other attackers and thus underlines the importance of the dynamics of transcriptional plant responses to multiple herbivory.

**Electronic supplementary material:**

The online version of this article (doi:10.1007/s00442-016-3758-3) contains supplementary material, which is available to authorized users.

## Introduction

Throughout the growing season, plants are commonly attacked by multiple herbivorous species. The responses of plants to one specific herbivore may impact the performance of other insects feeding on the same host plant (Rodriguez-Saona et al. 2010; Soler et al. 2012; Zhang et al. 2013; Stam et al. 2014). Interestingly, defenses induced in response to multiple insect feeding (Rodriguez-Saona et al. 2010; Tzin et al. 2015b; Onkokesung et al. 2016), can have positive or negative effects on the performance of one of the attacking herbivores (Soler et al. 2012; Ali et al. 2014; Li et al. 2014; Kroes et al. 2015). For example, while oleander aphids (*Aphis nerii*) developed more slowly on milkweed plants (*Asclepias syriaca*) previously infested by monarch caterpillars (*Danaus plexippus*) (Ali et al. 2014), *Brevicoryne brassicae* aphids developed faster on cabbage plants previously infested by *Pieris brassicae* caterpillars (Soler et al. 2012). Studies on the molecular aspects of plant-mediated interactions among multiple insects have shown that transcriptomic responses to multiple attacks are clearly different from responses to attacks by individual insects (Voelckel and Baldwin 2004; Rodriguez-Saona et al. 2010; Zhang et al. 2013; Coolen et al. 2016; Davila Olivas et al. 2016). For instance, tomato plants (*Solanum lycopersicum*) exhibited a different transcriptomic response to simultaneous attack by *Spodoptera exigua* caterpillars and *Macrosiphum euphorbiae* aphids than to attack by the caterpillar or aphid alone (Rodriguez-Saona et al. 2010). This study also demonstrated that aphid feeding suppressed the expression of caterpillar-induced genes and, vice versa, caterpillar feeding down-regulated the expression of genes up-regulated by aphids (Rodriguez-Saona et al. 2010). In dually infested *Arabidopsis thaliana* plants, the phloem-feeding whitefly *Bemisia tabaci* suppressed the expression of genes up-regulated by *Plutella xylostella* caterpillars (Zhang et al. 2013).

The differential effects of multiple insect feeding on induced defenses may not only be explained by species differences, but may also be dependent on insect density (Zhang et al. 2009; Kroes et al. 2015; Stewart et al. 2016). For instance, *B. brassicae* aphids had differential plant-mediated effects on the performance of *P. xylostella* caterpillars: at low aphid density caterpillar growth rate was enhanced while it was reduced at high aphid density (Kroes et al. 2015). Therefore, investigating effects of insect density on plant defense responses can provide novel insights in plant-mediated interactions between multiple attacking insects. Investigating how insects belonging to different feeding guilds affect each other’s individually induced plant transcriptional response, while including effects of insect density, is an important step towards unravelling plant responses to simultaneously feeding herbivores.

The phytohormones jasmonic acid (JA) and salicylic acid (SA) have been recognized to play key roles, with ethylene (ET) and abscisic acid (ABA) as co-regulators, in mediating plant defense responses against herbivorous insects (Erb et al. 2012; Broekgaarden et al. 2015). The different phytohormone signalling pathways operate in an interacting network allowing the plant to activate an adequate defense response depending on the feeding guild and species identity of the attacking insect (De Vos et al. 2005; Bidart-Bouzat and Kliebenstein 2011; Robert-Seilaniantz et al. 2011; Derksen et al. 2013; Pieterse et al. 2012; Appel et al. 2014). Specific nodes in this network, such as the transcriptional regulators WRKY70 and ORA59, integrate phytohormonal signalling and regulate plant defenses in response to herbivory (Caarls et al. 2015).

Plant defenses induced by caterpillars are mainly regulated by the phytohormone jasmonic acid (JA) and its derivatives such as methyl jasmonate (MeJA) and jasmonic acid-isoleucine (JA-Ile) (Thaler et al. 2002; Turner et al. 2002; Halitschke and Baldwin 2003; Koo and Howe 2009; Verhage et al. 2011; Rehrig et al. 2014). JA-Ile binds to the CORONATINE INSENSITIVE 1 (COI1) receptor thereby mediating the degradation of JAZ repressor proteins (Thines et al. 2007). In the absence of JA, these JAZ proteins bind to the transcription factor MYC2 that consequently prevents the regulation of JA-responsive genes such as *PLANT DEFENSIN 1.2* (*PDF1.2*) and *VEGETATIVE STORAGE PROTEIN 2* (*VSP2*) (Lorenzo et al. 2004; Memelink 2009; Kazan and Manners 2013). The JA-signalling pathway consists of two interconnected branches, either regulated by MYC2 or ERF (Lorenzo et al. 2003), that cross-communicate with the ET and ABA pathways depending on the plant attacker (Lorenzo and Solano 2005; Pieterse et al. 2012; Kazan and Manners 2013). MYC2 regulates the biosynthesis of defensive secondary metabolites such as glucosinolates (Dombrecht et al. 2007; Kazan and Manners 2013) and terpenoids (Hong et al. 2012; Kazan and Manners 2013). Different from leaf chewing by caterpillars, aphids feed on the plant’s phloem by inserting their stylets into the sieve elements (De Vos et al. 2007; Stam et al. 2014). Regulation of plant defenses to aphid feeding is mainly mediated by SA, but involvement of JA and ET has also been reported (Moran et al. 2002; De Vos et al. 2005; Couldridge et al. 2007; Kusnierczyk et al. 2008; Barah et al. 2013; Appel et al. 2014; Hillwig et al. 2016).

Defense mechanisms and the underlying phytohormonal signalling have been intensively studied for *A. thaliana* in combination with herbivorous insects from the two main insect feeding guilds, i.e. leaf chewers and phloem feeders. Defense responses induced by leaf chewers such as *P. xylostella* caterpillars mainly involve the activation of JA-regulated genes (Stotz et al. 2000; Ehlting et al. 2008; Herde et al. 2008; Bidart-Bouzat and Kliebenstein 2011; Zhang et al. 2013; Kroes et al. 2015), while phloem feeders such as *B. brassicae* aphids enhance the expression of genes involved in SA-dependent defenses (Moran et al. 2002; Kusnierczyk et al. 2008; Barah et al. 2013). In addition, Barah et al. (2013) found *B. brassicae*-induced expression of genes related to the biosynthesis of tryptophan-derived secondary metabolites, the ET signalling pathway, as well as to cell wall metabolism (Kusnierczyk et al. 2008). Moreover, also JA-regulated defenses have been found to be involved in responses to aphid feeding (Moran et al. 2002; Kusnierczyk et al. 2008; Morkunas et al. 2011). In the present study, we investigated how phloem feeding by *B. brassicae* aphids affects the transcriptional response of *A. thaliana* to leaf-chewing *P. xylostella* caterpillars. This study builds upon our recent finding that *B. brassicae* aphids feeding on *A. thaliana* plants at low or high aphid densities differentially affect defense of the plants to *P. xylostella* and that this is mediated by JA and SA signalling (Kroes et al. 2015). Here, we compared transcriptomic responses of *A. thaliana* plants to simultaneous feeding by *P. xylostella* caterpillars and *B. brassicae* aphids to transcriptomic responses of plants to infestation by *P. xylostella* caterpillars alone. We particularly addressed the question whether the transcriptomic response to simultaneous attack by aphids and caterpillars is affected by aphid density. To study density-dependent effects on transcriptomic responses, plants were infested with a low or high aphid density according to the same methods as we used previously (Kroes et al. 2015). In addition, transcriptional responses were studied at two time points to study the dynamics of simultaneous herbivory.

## Materials and methods

### Plant growth conditions

Seeds of *Arabidopsis thaliana* accession Columbia-0 (Col-0) were sown in autoclaved (80 °C for 4 h) potting soil (Lentse potgrond, Lent, The Netherlands). Plants were cultivated in a growth chamber at 21 ± 2 °C under an 8L:16D cycle [200 μmol m^−2^ s^−1^ photosynthetically active radiation (PAR) light intensity] and 60 ± 10 % relative humidity (RH). Two-week-old seedlings were transferred to individual pots (5 cm diameter) containing similar soil. Plants were watered three times per week. Five-week-old plants were exposed to different insect-infestation treatments. During the experiments, all plants remained in the vegetative state.

### Insects

Both the Cabbage aphid, *B. brassicae* L. (Hemiptera: Aphididae), and the Diamondback moth, *P. xylostella* L. (Lepidoptera: Yponomeutidae), were reared on Brussels sprouts plants (*Brassica oleracea* var *gemmifera* cv Cyrus) at 22 ± 1 °C, 50–70 % RH, 16L:8D cycle.

### Insect infestation treatments

Plants were infested with (1) two second-instar (L2) caterpillars (indicated as single infestation); (2) simultaneously infested with two L2 caterpillars plus a low density of five adult aphids (abbreviated as Dual LD for Dual Low Density); (3) simultaneously infested with two L2 caterpillars plus a high density of 25 adult aphids (abbreviated as Dual HD for Dual High Density), or (4) left uninfested (indicated as control). Insects were allowed to feed freely on the plants.

Individual plants were placed in a plastic container (diameter 8 cm × height 14 cm), covered with gauze cloth and closed with elastic bands. Containers were randomly distributed in a tray (12–15 containers per tray). Trays were placed in a growth chamber with a 16L:8D cycle [200 μmol m^−2^ s^−1^ PAR], at 21 ± 2 °C and 50–70 % RH.

Leaves damaged by insect feeding or control leaves from uninfested plants were collected after 24 or 48 h of infestation. The experiment was performed in two rounds starting on two successive days (Feb 2015). For each treatment and time point, four biological replicates were obtained by performing two biological replicates per round. One biological replicate consisted of six leaves pooled from three different plants. For each time point, a different set of plants was used. Insects were removed from the leaves before harvesting. Leaf samples were flash-frozen in liquid nitrogen and stored at −80 °C prior to analysis.

### RNA extraction and microarray hybridization

Total RNA was extracted from finely ground frozen leaf tissue using the RNeasy Plant Mini Kit (Qiagen, Hilden, Germany). RNA samples were treated with DNase (Qiagen, Hilden, Germany). The concentration and purity of RNA was determined by spectrophotometry and integrity was confirmed using an Agilent 2100 Bioanalyzer with the RNA 6000 Nano Kit (Agilent Technologies, Palo Alto, CA, USA). Whole-genome transcriptome analysis was conducted by hybridizing four biological samples of total RNA per treatment to Affymetrix Arabidopsis Gene 1.1 ST Array Strips (Affymetrix, Santa Clara, CA, USA).

### Microarray data analysis

The raw data files (CEL files) were normalized using the Robust Multi-array Average (RMA) background correction with quantile normalization, log_2_ transformation and mean probe-set summarization with adjustment for GC content. Normalized gene expression data obtained from the microarray experiments were initially statistically analysed with one-way and two-way ANOVA using the software TIGR MeV version 4.9 (Saeed et al. 2003, 2006) to study the effects of treatment, time point and their interaction on gene expression levels, with *α* = 0.05. Expression ratios of the genes significantly differentially expressed between the four treatment groups (Control, *P. xylostella*, Dual LD and Dual HD) and time points (24 and 48 h) were then used for further analysis.

#### Partial least squares discriminant analysis (PLS-DA) data analysis

Changes in the expression pattern of genes that were significantly different between treatments were analysed using projection to latent structures discriminant analysis (PLS-DA; Eriksson et al. 2013) using SIMCA-P+ version 14.0 statistical software (Umetrics AB, Umeå, Sweden). The analysis determines whether samples from different treatment groups can be separated on the basis of differences in their gene expression patterns. The results of the analysis are visualized in score plots. The score plot identifies patterns that discriminate the treatments according to model components of PLS-DA. The quality of each OPLS-DA model was evaluated using the parameter *R* ^2^ (goodness of fit) and *Q* ^2^ (predictive value) (Eriksson et al. 2013).

#### Differentially expressed genes

Differentially expressed genes (DEGs) were identified per time point for the different single and dual-infestation treatments. Differential gene expression in caterpillar- or dual-infested plants was determined compared to expression in uninfested control plants, with gene expression in dually infested plants additionally being compared to expression in *P. xylostella*-infested plants.

Genes were considered to be differentially regulated in a given pair of treatments if a *t* test demonstrated a significant result at *P* < 0.05 (accepting a false discovery rate of up to 0.2; Ehlting et al. 2008) and a log_2_-fold change of ≤−1 or ≥1 (TIGR MeV v4.9).

#### Functional enrichment

Identification and enrichment of DEGs within functional gene ontology (GO) terms for biological processes was done using the online tool provided by DAVID Bioinformatics Resources (http://david.abcc.ncifcrf.gov/; Huang et al. 2009). Only enrichment groups with an enrichment score ≥1.3 were examined (Huang et al. 2009). Genes were considered statistically enriched if Fisher’s exact test (EASE score) resulted in *P* < 0.05 and if the Benjamini–Hochberg correction for multiple comparisons returned *P* < 0.05.

#### Hierarchical clustering

Genes differentially expressed at 24 and 48 h after single *P. xylostella* or dual *P. xylostella* and *B. brassicae* infestation at low or high aphid density (measured relative to uninfested control samples) were organized further by hierarchical clustering. Hierarchical clustering analysis was performed with the Spearman rank correlation using average linkage in the software TIGR MeV version 4.9.

### Validation of microarray analysis by quantitative real-time PCR

cDNA was synthesized from the same RNA (1 µg) isolated for the microarray hybridization as described in the ‘RNA extraction and microarray hybridization’ section using iScript cDNA synthesis Kit (Bio-Rad). Transcript levels of the genes *TERPENE SYNTHASE 04* (*TPS04*) (At1g61120) (Snoeren et al. 2010), *VEGETATIVE STORAGE PROTEIN 2* (*VSP2*) (At5g24770) (Anderson et al. 2004) and *PLANT DEFENSIN 1.2* (*PDF1.2*) (At5g44420) (Anderson et al. 2004) and the reference gene *ELONGATION FACTOR 1α* (*EF1*-*α*) (At5g60390) (Remans et al. 2008) were quantified. Efficiency of each primer was determined before qRT-PCR analysis. Quantitative RT-PCR analysis was performed in a CFX96 Touch™ Real-Time PCR Detection System (Bio-Rad). Each reaction was performed in a total volume of 25 µl containing 12.5 µl SYBR Green Supermix (Bio-Rad), 5 µl cDNA and 1 µl of 10 µM forward and reverse gene-specific primer pair. For each reaction, two technical replicates were performed and average values were used in the analyses. The following thermal profile was used: 3 min 95 °C, followed by 40 cycles of 15 s at 95 °C, and 45 s at 60 °C.

Relative expression for each tested gene was calculated by using the 2^−ΔΔCt^ method (Livak and Schmittgen 2001) and subsequently log_2_ transformed. Relative expression levels of *TPS04*, *VSP2* and *PDF1.2* were compared to their respective log_2_-expression ratios found using microarray analysis (Online Resource 1).

## Results

### Transcriptomic changes in plants in response to feeding by *P. xylostella* alone or by both *P. xylostella* and *B. brassicae*

**Fig. 1:**
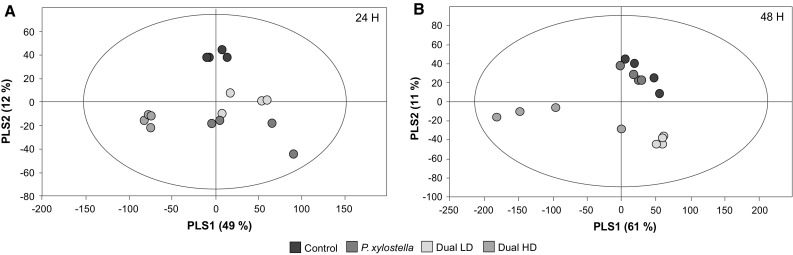
Partial least squares discriminant analysis (PLS-DA) of gene expression levels in *A. thaliana* at 24 h (**a**) and 48 h (**b**) after single *P. xylostella*, dual *P. xylostella* and *B. brassicae* and without infestation (control). Plants were infested with either a low (LD, 5 aphids per plant) or high (HD, 25 aphids per plant) density of *B. brassicae* aphids. The PLS-DA resulted in two models with six (24 h; *R* ^2^ *X* = 0.80, *R* ^2^ *Y* = 0.99 and *Q* ^2^ = 0.92) and five (48 h; *R* ^2^ *X* = 0.83, *R* ^2^ *Y* = 0.98 and *Q* ^2^ = 0.87) significant components, respectively. The *score plots* of the treatment samples at 24 and 48 h, with the percentage of explained variation in *parentheses*, is shown. The *ellipse* in the *score plots* defines the Hotelling’s T2 confidence region (95 %)

Transcriptional profiles in *A. thaliana* after 24 h (Fig. 1a) and 48 h (Fig. 1b) to feeding by *P. xylostella* only, or dual infestation by *P. xylostella* plus *B. brassicae* at low and high density and without infestation were analysed by PLS-DA using expression levels of all genes that showed significant differences in expression level between treatments (based on one-way ANOVA).

At 24 h, the first two significant PLS components explain 49 and 12 % of the total variance, respectively (Fig. 1a). The first component shows a clear separation between transcriptional profiles of dually infested plants at high density (Dual HD) versus the other three treatments, while the second component separated the profiles based on the presence or absence of herbivores.

At 48 h, the first two significant PLS components explain 61 and 11 % of the total variance, respectively (Fig. 1b). As was found for the 24-h time point, the first component shows a clear separation of transcriptional profiles between Dual HD plants versus the other three treatments, while the second component separates the profiles based on the presence of aphids. Interestingly, when comparing the position of *P. xylostella*-infested samples between the PLS-DA models of 24 and 48 h, the gene expression profiles in response to caterpillar feeding differs more strongly from that of non-infested plants after 24 h, whereas the profiles have converged after 48 h.

### Differentially expressed transcripts in response to *P. xylostella* alone or in response to both *P. xylostella* and *B. brassicae*

To identify differentially expressed genes (DEGs) in *A. thaliana* in response to caterpillars feeding alone or simultaneous feeding of caterpillars and aphids, we compared the expression of genes in uninfested control plants to that in plants treated with herbivores.

**Fig. 2:**
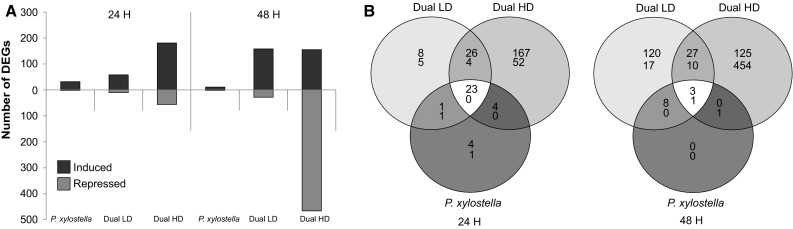
Differentially expressed genes (DEGs) (**a**) and Venn diagram of number of DEGs (**b**) in *A. thaliana* at 24 and 48 h after single *P. xylostella*, dual *P. xylostella* and *B. brassicae* and without infestation (control). Plants were infested with either a low (LD, 5 aphids per plant) or high (HD, 25 aphids per plant) density of *B. brassicae* aphids. Number of DEGs in *P. xylostella*-infested, Dual LD and Dual HD plants were compared with non-infested control plants. *Red bars* or *numbers* indicate up-regulated genes, while *green bars* or *numbers* represent down-regulated genes. Genes were considered to be differentially expressed if they met the criteria of log_2_-fold change ≤−1 or ≥1 and a *t* test *P* value <0.05

The number of DEGs induced in response to feeding by *P. xylostella* was larger than the number of repressed genes (Fig. 2a). However, the number of induced genes decreased over time. When *P. xylostella* caterpillars were feeding simultaneously with *B. brassicae* aphids, the number of DEGs was higher after 24 and 48 h compared to the response to caterpillars feeding alone (Fig. 2a). Interestingly, there was an aphid-density effect on the number of DEGs over time (Fig. 2a). After 24-h dual HD plants showed a larger number of DEGs compared to dual aphid and caterpillar infestation at low aphid density (Dual LD). More repressed DEGs were found after 48 h in Dual HD plants, while the number of induced DEGs was comparable to that in Dual LD plants after 48 h (Fig. 2a).

In total, only 10 % of induced DEGs were shared among the different treatments at 24 h (Fig. 2b). Moreover, the treatments only shared 1 % of their induced genes at 48 h (Fig. 2b). Thus, *A. thaliana* responses to aphids and caterpillars feeding simultaneously or caterpillars feeding alone are highly dissimilar.

Almost all DEGs in response to *P. xylostella* feeding were shared with DEGs found in Dual LD or Dual HD plants at both 24 and 48 h (Fig. 2b). Respectively, 41 % of up-regulated genes in response to Dual LD and 12 % of up-regulated genes in response to Dual HD were shared with DEGs up-regulated by *P. xylostella* feeding at 24 h. At 48 h, a low proportion of up-regulated DEGs (<7 %) in response to Dual LD and Dual HD were shared with DEGs up-regulated in response to *P. xylostella* feeding. Thus, the presence of aphids on *P. xylostella*-infested plants increased the number of DEGs, while the number of DEGs in response to *P. xylostella* feeding did not change.

In conclusion, dual herbivory by aphids and caterpillars resulted in different transcriptional responses compared to those induced by *P. xylostella* caterpillars feeding alone. Furthermore, specificity in transcriptional responses to simultaneous feeding of both herbivores or caterpillars feeding alone increased over time.

#### Gene clustering and GO terms

To identify biological functions of the DEGs we assigned GO terms for biological processes and performed a functional clustering analysis using the DAVID Functional Clustering Tool (Online Resource 2 and 3).

For *P. xylostella*-induced genes (at both 24 and 48 h), the clusters mainly relate to responses to biotic stress, including pathogen infection and wounding, and jasmonic acid stimuli. However, when caterpillars feed simultaneously with aphids at either of the two densities, clusters also associated with metabolism of organic acids, fatty acids and lipids.

After 48 h in Dual HD plants, repressed DEGs mainly clustered in classes that relate to photosynthesis and carbohydrate metabolism, including genes encoding for thioredoxins and glutaredoxins, Photosystem (PS I and II) proteins, PsbP proteins and proteins involved in the glycolysis pathway.

### Differentially expressed transcripts under dual herbivory at low or high aphid density

**Fig. 3:**
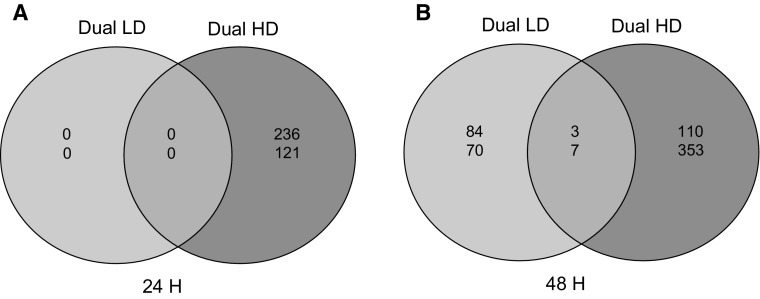
Venn diagram representing numbers of genes differentially expressed (DEGs) in *A. thaliana* at 24 h (**a**) and 48 h (**b**) after single *P. xylostella* and dual infestation by *P. xylostella* plus *B. brassicae*. Plants were infested with either a low (LD, 5 aphids per plant) or high (HD, 25 aphids per plant) density of *B. brassicae* aphids. Number of DEGs specifically expressed or co-expressed in the Dual LD and Dual HD treatments were compared with single *P. xylostella* infestation. *Numbers in red* indicate up-regulated genes, while *numbers in green* represent down-regulated genes. Genes were considered differentially expressed if they met the criteria of log_2_-fold change ≤−1 or ≥1 and a *t* test *P* value <0.05

To investigate how aphid density influences effects on transcriptional responses to caterpillar herbivory, differential gene expression was determined in comparison to expression in caterpillar-infested plants. We examined differences and overlap in DEGs compared to caterpillar-only treatment between Dual LD plants on the one hand and Dual HD plants on the other hand, both when the plants had been exposed to herbivory during 24 and 48 h (Fig. 3a, b).

There was no effect of a low aphid density on DEGs compared to caterpillar-only treatment after 24 h (Fig. 3a). However, a low aphid density caused changes in responses after 48 h (Fig. 3b). At 48 h, a total of 87 up-regulated and 77 down-regulated DEGs, compared to caterpillar-only treatment were found in Dual LD plants. Of these DEGs, only 2 % up- and down-regulated genes were shared with DEGs in Dual HD plants.

A total of 236 up-regulated DEGs after 24 h and 113 up-regulated DEGs after 48 h, in comparison to caterpillar-only treatment, respectively, were found in Dual HD plants. In addition, 121 and 360 DEGs were down-regulated after 24 and 48 h, respectively.

In conclusion, when investigating how aphid density influences differential expression compared to caterpillar-only treatment between Dual LD and Dual HD after 48 h, DEGs have very little overlap which indicates that DEGs are highly density-specific.

#### Gene clustering and GO terms

To identify biological functions of these genes, we assigned GO terms for biological processes and performed a functional clustering analysis using the DAVID Functional Clustering Tool (Online Resource 2 and 3).

After 48 h in Dual LD plants, up-regulated genes were associated with defense, cell death and auxin signalling. Repressed genes could not be clustered.

In Dual HD plants after 24 h, up-regulated genes in comparison to caterpillar-only treatment were associated with transcriptional responses to hormone signalling (ABA- and auxin-activated signalling pathways) and carbohydrate metabolism. Repressed genes could not be clustered. At 48 h, up-regulated genes could not be clustered. For repressed genes, clusters relate to carbohydrate metabolism, photosynthesis, responses to bacterium (several *WRKY* transcription factor genes) and biogenesis of cellular components.

### Clustering of gene expression levels

We compared gene expression patterns of *A. thaliana* in response to feeding by *P. xylostella* caterpillars alone and simultaneous feeding by caterpillars and aphids at two different densities to further investigate the effect of simultaneous aphid feeding and aphid density on responses to caterpillars.

#### Clustering after 24 h of herbivory (based on one-way ANOVA)

The cluster analysis shows similarities in gene expression levels in response to *P. xylostella* feeding alone and to Dual LD because both treatments cluster together and are separate from gene expression levels in response to Dual HD (Online Resource 4).

**Fig. 4:**
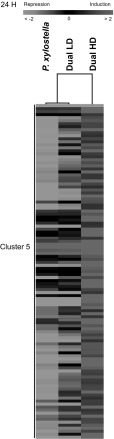
Heat map showing average log_2_-fold change ratios (measured relative to non-infested control samples) of genes expressed in *A. thaliana* at 24 h after single *P. xylostella* or dual *P. xylostella* and *B. brassicae* infestation. Plants were infested with either a low (LD) or high (HD) density of *B. brassicae* aphids. Hierarchical clustering (HCL) was performed using Spearman correlation with average linkage clustering. *Red* indicates up-regulated genes, while *green* shows down-regulated genes. *Black* represents no change in expression. Each *row* in the *columns* corresponds to a single gene. Cluster analysis is shown for cluster 5

Cluster 5 is clearly different across treatments and consists of 111 genes that were more repressed in response to *P. xylostella* feeding and to Dual LD compared to Dual HD (Fig. 4). Cluster 5 contains genes involved in defense responses (*MES7*, *PDF1.2B*, *PDF1.2*, *CCR2*, *PGIP2, ERD5*), in responses to phytohormones (*TTL3*, *ACR4*, *BT4*, *PYL4*, *PYL5*) and genes associated with cell wall remodelling (*PME3*, *EXT3*, *FLA13*, *AGP16*) (Online Resource 5A).

#### Clustering after 48 h of herbivory (based on one-way ANOVA)

Gene expression levels in response to *P. xylostella* feeding alone and to Dual HD cluster separately from those in response to Dual LD. This result shows that aphid density affects gene expression pattern and responses induced by caterpillar feeding are more similar to those induced by Dual HD (Online Resource 6).

**Fig. 5:**
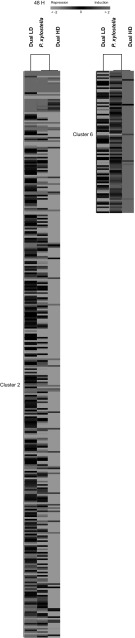
Heat map showing average log_2_-fold change ratios (measured relative to non-infested control samples) of genes expressed in *A. thaliana* at 48 h after single *P. xylostella* or dual *P. xylostella* and *B. brassicae* infestation. Plants were infested with either a low (LD) or high (HD) density of *B. brassicae* aphids. Hierarchical clustering (HCL) was performed using Spearman correlation with average linkage clustering. *Red* indicates up-regulated genes, while *green* shows down-regulated genes. *Black* represents no change in expression. Each *row* in the *columns* corresponds to a single gene. Cluster analysis is shown for clusters 2 and 6

For example, cluster 2 consists of genes that are more repressed in response to Dual HD and *P. xylostella* feeding compared to Dual LD (Fig. 5). Cluster 2 consists of 343 genes including genes involved in plant defense signalling (such as genes encoding TIFY protein family, *RIPK*, hevein-like protein, *GLR3.4*, MYB domain proteins and peroxidases), responses to phytohormones (such as genes involved in ABA, auxin and SA signalling), and photosynthesis (such as genes encoding for thioredoxins and glutaredoxins, Photosystem (PS I and II) proteins, PsbP proteins and proteins involved in the glycolysis pathway) (Online Resource 5B). In addition, cluster 2 consists of genes involved in JA-mediated induced plant defenses (*CHL1*, *JAZ9*, *JR1*, *NATA1*, *CORI3, JAZ1*, *PR4*, *JAZ2*, *PGIP2*, *AOC2*, *PDF1.2b*, *MES18*) and genes involved in the biosynthesis of isopentenyl diphosphate and carotenoid (terpenoid metabolic processes) (Online Resource 5B). However, cluster 6 consists of 86 genes that were more induced in response to feeding by *P. xylostella* caterpillars and to Dual HD compared to Dual LD (Fig. 5). Cluster 6 contains genes involved in secondary metabolism (*CYP706A2*, *CYP71B8*, *CYP710A3*), responses to phytohormones (ethylene and auxin response factors) and genes encoding transcription factors (such as MADS-box and NAC domain proteins) which are involved in controlling all major aspects of development and hormone signalling (Online Resource 5B).

#### Clustering over time (based on two-way ANOVA analysis)

When gene expression levels were clustered by time point and treatment, responses induced by Dual LD during 48 h and Dual HD during 24 h clustered together, indicating that insects attacking at high densities cause an acceleration in plant responses compared to insects attacking at low density (Online Resource 7). Moreover, plants with Dual LD treatment during 24 h and plants with Dual HD treatment during 48 h clustered together, suggesting that responses to Dual HD after 48 h diminish to levels found after 24 h of Dual LD (Online Resource 7). However, responses to dual infestations at both densities after 24 and 48 h, cluster separately from responses to dual infestations at low and high aphid density after 48 and 24 h, respectively. This indicates that responses between the two time points are distinctly different (Online Resource 7).

**Fig. 6:**
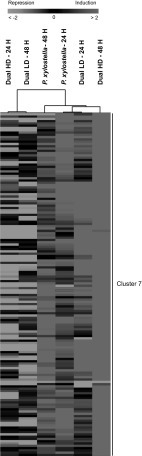
Heat map showing average log_2_-fold change ratios (measured relative to non-infested control samples) of genes expressed in *A. thaliana* at 24 and 48 h after single *P. xylostella* or dual *P. xylostella* and *B. brassicae* infestation. Plants were infested with either a low (LD) or high (HD) density of *B. brassicae* aphids. Hierarchical clustering (HCL) was performed using Spearman correlation with average linkage clustering. *Red* indicates up-regulated genes, while *green* shows down-regulated genes. *Black* represents no change in expression. Each *row* in the *columns* corresponds to a single gene. Cluster analysis is shown for cluster 7

For instance, in cluster 7, 150 genes were found that were more up-regulated by caterpillar feeding during 24 and 48 h, Dual LD during 24 h and Dual HD during 48 h compared to the other treatments (Fig. 6). Several regulatory genes involved in defense responses and disease resistance (*WRKY49, WRKY74, WRKY64*), genes encoding MYB domain proteins and genes involved in secondary metabolism (*CYP706A2*, *CYP71B8*, *CYP710A3*, *CYP71A28*) belong to this cluster (Online Resource 5C). In addition, cluster 7 consists of genes encoding transcription factors such as MADS-box, genes involved in defense responses (such as *PROPEP3*, *MLO5*, *MLP329*, *FRK1* and *LRC29*, *LRC17*, *LRC37*) and phytohormone-mediated signalling (such as *ERF115*, *EIL2* and *ARF23*) (Online Resource 5C).

## Discussion

Plants activate a complex array of defense reactions in response to feeding by insect herbivores (Kessler and Baldwin 2002; Mithofer and Boland 2012). Plants respond differently to leaf-chewing and piercing-sucking insects (De Vos et al. 2005; Bidart-Bouzat and Kliebenstein 2011; Appel et al. 2014). Here, we add to this knowledge by showing that herbivore density is an important modulator of such specific transcriptional responses of plants.

Studying the plant-mediated interactive effects of two insect attackers belonging to different feeding guilds can provide novel insights into the molecular aspects of plant-mediated interactions among multiple insects. In the present microarray analysis, we found differences in gene expression in *A. thaliana* plants induced by *P. xylostella* caterpillars alone compared to infestation by a combination of *P. xylostella* caterpillars and *B. brassicae* aphids (Figs. 1, 2). Only a few studies have investigated the molecular aspects of plant-mediated interactions among multiple insects (Voelckel and Baldwin 2004; Rodriguez-Saona et al. 2010; Zhang et al. 2013). Voelckel and Baldwin (2004) found that transcriptional responses of *Nicotiana attenuata* plants to simultaneous attack by the sap-feeding insect *Tupiocoris notatus* and the chewing caterpillar *Manduca sexta* were different from those from either herbivore alone. Furthermore, simultaneous feeding by the aphid *Macrosiphum euphorbiae* and the caterpillar *Spodoptera exigua* on tomato plants resulted in a different pattern of gene expression compared to transcriptional responses induced by caterpillars or aphids alone (Rodriguez-Saona et al. 2010). In *A. thaliana* plants, feeding by the whitefly *Bemisia tabaci* suppressed the up-regulation of a large number of genes induced by *P. xylostella* caterpillars (Zhang et al. 2013). In contrast, we identified a larger number of DEGs in response to simultaneous feeding by *P. xylostella* caterpillars and *B. brassicae* aphids compared to caterpillars feeding alone. This may indicate that aphids and whiteflies, although both phloem feeders, interfere in a different way with caterpillar-induced defenses and cautions against generalizations based on feeding guild. Moreover, based on our data showing that herbivore density can also be an important factor modulating plant-mediated interactions, this may provide an additional explanation for the differences between results of the studies on the effects of aphids and whiteflies.

When comparing transcriptional responses of *A. thaliana* plants exposed to caterpillars feeding alone or to simultaneous feeding by caterpillars and aphids, different plant responses are induced. We observed up-regulation of several JA-responsive genes (*PR4, HEL*, *VSP1*, *PDF1.2*, *TPS04*, *CORI3*, *JR1*) and genes involved in JA signal-transduction (*JAZ5*, *JAZ9*) in response to feeding by *P. xylostella* caterpillars (Online Resource 3). Several of these genes were also found to be up-regulated by *P. xylostella* infestation in a microarray study (Ehlting et al. 2008), which suggests that JA-mediated responses play an important role in plant defense against *P. xylostella* caterpillars (Zhang et al. 2013). In response to simultaneous aphid feeding, also genes related to metabolism of organic acids, fatty acids and oxylipins were up-regulated, compared to *P. xylostella* caterpillars feeding alone. Oxylipins are involved in plant responses to insect attack (Bostock 2005). For instance, oxylipin-related genes were up-regulated by *P. xylostella* feeding in *Arabidopsis* plants (Ehlting et al. 2008). Interestingly, also in response to aphid feeding, oxylipins are induced in *A. thaliana* and maize plants (Kusnierczyk et al. 2008; Tzin et al. 2015a). Therefore, oxylipins may be important for induced defenses in response to dual caterpillar and aphid infestation. Transcriptional interference between simultaneously feeding insect herbivores can lead to positive or negative effects on the performance of the herbivores. For example, in cabbage (*Brassica oleracea*) positive effects of *B. brassicae* aphid feeding on the performance of *Pieris brassicae* caterpillars were observed (Soler et al. 2012). In addition, caterpillars of the Monarch butterfly *Danaus plexippus* were positively affected on milkweed plants previously infested by oleander aphids *Aphis nerii*, whereas the aphids were negatively affected on milkweed plants previously infested by conspecific caterpillars (Ali et al. 2014).

### Effect of insect density on transcriptional responses

Induced plant responses to multiple herbivory can be influenced by the density of the attacking insects. For instance, interference by *B. brassicae* aphids with induced defenses against caterpillars depends on the density of the attacking aphids (Kroes et al. 2015; Ponzio et al. 2016). As a next step in the study of density-dependent interference of aphids with caterpillar-induced defenses, we studied the effect of different aphid densities on whole-genome transcriptional responses of *A. thaliana* to feeding by *P. xylostella* caterpillars.

We observed that transcriptional responses of *A. thaliana* to feeding by *P. xylostella* caterpillars were aphid density-dependent. There are differences in the nature of the differentially expressed genes when comparing plants with Dual LD and Dual HD treatments with caterpillar-infested plants after 48 h (Fig. 3). We found several *WRKY* transcription factor genes (*WRKY33*, *WRKY40* and *WRKY70*) only repressed in response to simultaneous aphid feeding at high density after 48 h. WRKY proteins belong to a large family of transcriptional regulators in *A. thaliana* plants (Rushton et al. 2010) and play an important role in regulating plant responses to pathogens (Pandey and Somssich 2009). For example, the transcription factor WRKY33 mediates defense responses to the necrotrophic fungus *Botrytis cinerea* in *A. thaliana* (Birkenbihl et al. 2012). Furthermore, WRKY70 has a key role in regulating interactions between SA- and JA-mediated signalling pathways (Li et al. 2004; Pieterse et al. 2012). Overexpression of WRKY70 induced the expression of SA-mediated *PR* genes, while it suppressed JA-responsive *PDF1.2* expression in *A. thaliana* plants (Li et al. 2004). It has been suggested that by activating the SA signalling pathway, aphids could interfere with JA-dependent defenses against caterpillars (Stam et al. 2014). Differential expression of *WRKY70* may underlie plant-mediated interactions between simultaneously attacking aphids and caterpillars. A negative correlation between SA-mediated *WRKY70* expression and JA-dependent *MYC2* expression in *A. thaliana* plants infested by both caterpillars and aphids was shown before and was also aphid-density dependent (Kroes et al. 2015). Expression of *WRKY70* was down-regulated in Dual HD plants, which led to the induction of JA-mediated defenses (Kroes et al. 2015). By activating JA-dependent defenses in response to simultaneous feeding of caterpillars and aphids at high density, plants could increase defense against aphids and caterpillars.

Also WRKY40 is involved in the crosstalk between JA and SA signalling (Xu et al. 2006). Moreover, WRKY40 negatively regulates ABA-responsive gene expression (Chen et al. 2010). The plant hormone ABA is an important modulator of plant defense responses (Morkunas et al. 2011; Lee and Luan 2012). Here, we detected ABA-dependent genes that were differentially induced in response to Dual HD after 24 h, compared to genes expressed in caterpillar-induced plants, but not in response to Dual LD (e.g. *ABF1*, *PYR1*, *PLC1*, *SRK2D* and *AHK2*). In addition, we found a group of genes (Cluster 5) that were more strongly up-regulated at 24 h in response to Dual HD compared to *P. xylostella* caterpillars feeding alone and to Dual LD (Fig. 4; Online Resource 5A). Cluster 5 contains the ABA receptors PYL4 and PYL5. These receptors inactivate plant PP2Cs, such as *ABI1* and *ABI2*, which are known to suppress ABA signalling (Ma et al. 2009; Park et al. 2009). Recently, it was shown that aphids feeding on *A. thaliana* and the legume *Medicago truncatula* increase ABA content in the plants (Guo et al. 2016; Hillwig et al. 2016). Furthermore, *M. persicae* aphid population development was negatively affected on ABA-deficient mutants compared to wild-type *A. thaliana* plants (Kerchev et al. 2013; Hillwig et al. 2016). Thus, these results suggest that plant responses to simultaneous caterpillar and aphid feeding involves ABA signalling, which is dependent on aphid density and decreases defense responses against the attacking aphids. To support this, performance of aphids at different densities feeding simultaneously with caterpillars on ABA-deficient mutants should be studied.

Activation of plant defenses in response to herbivory is costly. It requires the diversion of resources away from plant growth (Huot et al. 2014) and herbivory suppresses photosynthesis (Zangerl et al. 2002; Voelckel and Baldwin 2004; Appel et al. 2014; Huot et al. 2014; Zhu et al. 2015). In response to *P. xylostella* feeding, or to Dual HD, we found a group of genes (Cluster 2) that were more strongly down-regulated after 48 h compared to Dual LD (Fig. 5; Online Resource 5B). This cluster contains genes associated with photosynthesis, which suggests that simultaneous feeding by caterpillars and aphids at a low or a high density has a different impact on the expression of photosynthesis-related genes. As a consequence, induction of plant defenses may be differentially affected in response to aphid feeding at low or high densities.

### The temporal dynamics of simultaneous herbivory

Transcriptional responses to feeding by a single herbivore species are highly dynamic over time (Ehlting et al. 2008; Kusnierczyk et al. 2008; Appel et al. 2014; Tzin et al. 2015a; Davila Olivas et al. 2016). Transcriptional responses to caterpillars feeding alone and feeding by both caterpillars and aphids changed over time. Similar to the finding of Ehlting et al. (2008) that more genes are differentially expressed at early time points in response to *P. xylostella* feeding, we found that a higher number of DEGs was up-regulated by *P. xylostella* caterpillar feeding after 24 h as compared to *P. xylostella*-induced DEGs after 48 h. In addition, Voelckel and Baldwin (2004) showed that specific transcriptional changes to *M. sexta* caterpillar infestation on tobacco plants occur after 24 h but these disappeared after 5 days of feeding.

Our cluster analysis showed that time-dependent transcripts in response to dual infestation were affected by the density of the attacking aphids. In response to Dual LD during 48 h, a similar transcriptional pattern was expressed as that found in response to Dual HD during 24 h. This indicates that insects attacking at a high density cause an acceleration in plant responses compared to insects attacking at a low density. Furthermore, we found that plant responses to Dual HD during 48 h clustered together with responses found after 24 h of Dual LD. Interestingly, many of the up-regulated genes from Cluster 7 in response to the two aphid densities at both time points are known to be involved in plant defenses (e.g. *LCR17*, *LCR29*, *FRK1*, *PROPEP3*, *MYB78*, *LCR37*, *MLP329* and different cytochrome P450 genes) (Fig. 6; Online Resource 5C). For example, *MYB78* belongs to the R2R3-type *MYB* genes which are involved in plant defense responses (Stracke et al. 2001; Dubos et al. 2010). In *A. thaliana* plants, *MYB78* was shown to play a role in the defense response regulated by JA against *Botrytis cinerea* and *Alternaria brassicicola* infection (Mengiste et al. 2003). Another example is *PROPEP3* which encodes precursor proteins that, upon perception by two closely related receptor kinases, PEPR1 and PEPR2, activate plant defense (Bartels et al. 2013). Upon feeding by *Spodoptera littoralis* caterpillars, *PROPEP3* is up-regulated in *A. thaliana* plants (Klauser et al. 2015). Furthermore, the performance of *S. littoralis* was positively affected when feeding on *pepr1 pepr2* double mutants (Klauser et al. 2015). Also, *FRK1* (flg22-induced receptor-like kinase 1) expression was shown to be up-regulated to a significantly higher level by *B. brassicae*-derived elicitors compared to water-infiltrated *A. thaliana* leaves (Prince et al. 2014). In addition, SA signalling is involved in the regulation of *FRK1* expression (Yi et al. 2014). This may indicate that *FRK1* is involved in defense signalling against *B. brassicae* feeding.

## Conclusion

We determined if aphids interfere with transcriptional responses of *A. thaliana* plants to *P. xylostella* caterpillars and whether this interference was dependent on aphid density. We show that the density of simultaneously feeding aphids has a differential effect on transcriptional responses in *A. thaliana* plants attacked by *P. xylostella* caterpillars, i.e. high aphid density has a stronger effect on caterpillar-induced responses than a low aphid density. In addition, transcriptomic responses are dynamic over time. In response to *P. xylostella* feeding alone, transcriptional changes were strongest after 24 h and mainly involved JA-responsive genes. When comparing gene expression patterns between time points and insect treatments, transcriptional patterns were similar between dual infestation at low density during 48 h and dual infestation at high density during 24 h. This indicates that insects attacking at a high density cause an acceleration in plant responses compared to insects attacking at low density. Furthermore, response to dual infestation at low density during 24 h and dual infestation at high density during 48 h mainly involved plant defense genes.

This study highlights the importance of addressing insect density and temporal dynamics to understand plant responses to dual or single insect attack. Mutant analysis studies are needed to confirm the function of genes involved in plant responses to single or dual insect attack.

## Electronic supplementary material

Below is the link to the electronic supplementary material.

Supplementary material 1 (PDF 901 kb)

Supplementary material 2 (PDF 297 kb)

Supplementary material 3 (XLSX 38 kb)

Supplementary material 4 (PDF 1055 kb)

Supplementary material 5 (PDF 365 kb)

Supplementary material 6 (PDF 536 kb)

Supplementary material 7 (PDF 657 kb)

Supplementary material 8 (PDF 2148 kb)

Supplementary material 9 (PDF 3816 kb)
