# Supplementary figures and images for: Brevicoryne brassicae aphids interfere with transcriptome responses of Arabidopsis thaliana to feeding by Plutella xylostella caterpillars in a density-dependent manner
Source: Oecologia. 2016 Oct 22;183(1):107–20. doi: 10.1007/s00442-016-3758-3 (PMC5239811; doi:10.1007/s00442-016-3758-3)

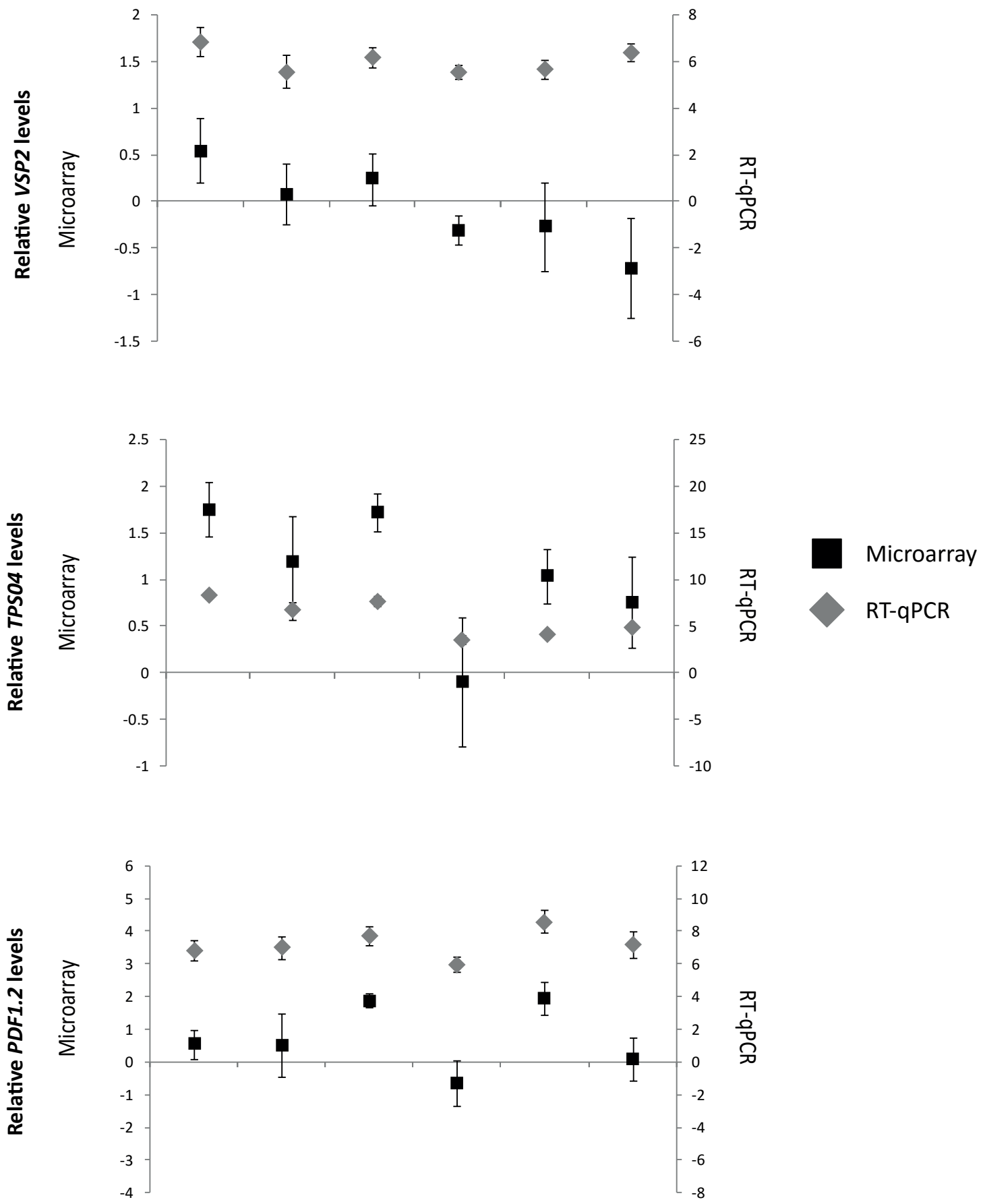

Supplement: Supplementary file 1 — Supplementary material 1 (PDF 901 kb) [file 442_2016_3758_MOESM1_ESM.pdf]

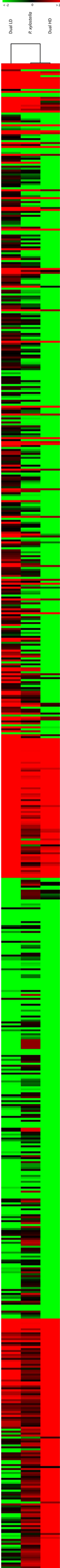

48 H

Cluster 2

Cluster 3

Cluster 1

Cluster 4

Cluster 5

Cluster 6

Supplement: Supplementary file 8 — Supplementary material 8 (PDF 2148 kb) [file 442_2016_3758_MOESM8_ESM.pdf]
