# Supplementary material for: Brevicoryne brassicae aphids interfere with transcriptome responses of Arabidopsis thaliana to feeding by Plutella xylostella caterpillars in a density-dependent manner
Source: Oecologia. 2016 Oct 22;183(1):107–20. doi: 10.1007/s00442-016-3758-3 (PMC5239811; doi:10.1007/s00442-016-3758-3)
Supplement: Supplementary file 2 — Supplementary material 2 (PDF 297 kb) [file 442_2016_3758_MOESM2_ESM.pdf]

Brevicoryne brassicae aphids interfere with the whole-genome transcriptional responses of *Arabidopsis thaliana* to feeding by *Plutella xylostella* caterpillars in a density-dependent manner

Oecologia

Anneke Kroes, Colette Broekgaarden, Marcos Castellanos Uribe, Sean May, Joop JA van Loon, Marcel Dicke
Wageningen University, annekekroes@hotmail.com

ESM 2. Functional clustering analysis (using DAVID Functional Annotation Clustering with enrichment score ≥ 1.3) for differentially expressed genes for pair-wise comparisons among Control (CT), *P. xylsotella* (PX), 'dual low density' (LD) and 'dual high density' (HD). Only GO clusters for biological processes are shown (* P < 0.05 for modified Fisher's exact test, ** P < 0.05 with Benjamini-Hochberg adjustment for multiple comparisons)

Biological Process GO Annotation		Induced								Repressed	
		24 h				48 h				48 h	
Identity	Type of Process	PX (vs CT)	LD (vs CT)	HD (vs CT)	HD (vs PX)	PX (vs CT)	LD (vs CT)	HD (vs CT)	LD (vs PX)	HD (vs CT)	HD (vs PX)
0006952	Defense response	**		**			**	*	*		
0042742	Defense response to bacterium									*	*
0050832	Defense response to fungus	**	**	**			**	*			
0009617	Response to bacterium									*	*
0010200	Response to chitin			*							
0009620	Response to fungus	**	**	**			**	*			
0009611	Response to wounding	*	**								
0042493	Response to drug			*							
0010033	Response to organic substance	**		**	**	**			*		
0010035	Response to inorganic substance						*				
0009651	Response to salt stress			**							
0006970	Response to osmotic stress			*							
0009725	Response to hormone			*	**		**		*		
0006979	Response to oxidative stress						*				
0009266	Response to temperature stimulus										
0009409	Response to cold			*							
0001906	Cell killing		*						*		
0031640	Killing of cells of other organism		*						*		
	Response to plant hormones										
0032870	Cellular response to hormone stimulus				*						

0009755	Hormone-mediated signaling pathway				*						
0009737	Response to abscisic acid stimulus				*						
0009738	Abscisic acid-activated signaling pathway				*						
0009733	Response to auxin				*				*		
0009753	Response to jasmonic acid stimulus	**	**	**		**	**	*			
0009695	Jasmonic acid biosynthetic process		**	**			*				
0009694	Jasmonic acid metabolic process		**	**			*				
	Cellular process										
0045454	Cell redox homeostasis									*	
0019725	Cellular homeostasis									*	
0022900	Electron transport chain						*		*	*	
0007167	Enzyme linked receptor protein signaling pathway									*	
0006091	Generatin of precursor metabolites and energy									*	
0035556	Intracellular signal transduction				*						
0007169	Transmembrane receptor protein tyrosine kinase signaling pathway									*	
	Cellular component biogenesis										
0022613	Ribonucleoprotein complex biogenesis										*
0042254	Ribosome biogenesis										*
	Cellular component organization										
0071555	Cell wall organization									*	
0045229	External encapsulating structure organization									*	
0009828	Plant-type cell wall loosening									*	
	Developmental process										
0006949	Syncytium formation									*	
	Metabolic process										
0051188	Cofactor biosynthetic process										*
0051186	Cofactor metabolic process									*	*
0018130	Heterocycle biosynthetic process										*
0042440	Pigment metabolic process										*
0006778	Porphyrin-containing compound metabolic										*
0033013	Tetrapyrrole metabolic process										*
0033014	Tetrapyrrole biosynthetic process										*

	Amino acid metabolism								
0006865	Amino acid transport							*	
0008652	Cellular amino acid biosynthetic process								
0009063	Cellular amino acid catabolic process								
	Carbohydrate metabolism								
0009743	Response to carbohydrate			*					
0016052	Carbohydrate catabolic process							*	
0008643	Carbohydrate transport			*				*	*
0034285	Response to disaccharide			*					*
0009750	Response to fructose			*					
0009749	Response to glucose			*					
0006007	Glucose catabolic process							*	
0006006	Glucose metabolic process							*	*
0006096	Glycolytic process							*	*
0009746	Response to hexose			*					
0019320	Hexose catabolic process							*	*
0019318	Hexose metabolic process							*	
0034284	Response to monosaccharide			*					
0046365	Monosaccharide catabolic process							*	*
0005996	Monosaccharide metabolic process							*	*
0005976	Polysaccharide metabolic process			*					
0044264	Cellular polysaccharide metabolic process			*					
	Fatty acid & lipid metabolism								
0006633	Fatty acid biosynthetic process	*	*						
0006631	Fatty acid metabolic process		*						
0008610	Lipid biosynthetic process	*	*						
0031408	Oxylipin biosynthetic process	**	**		**				
0031407	Oxylipin metabolic process	**	**		**				
	Organic substance metabolism								
0046164	Alcohol catabolic process							*	*
0009309	Amine biosynthetic process								
0015837	Amine transport							*	
0046394	Carboxylic acid biosynthetic process	*	**		*				

0016053	Organic acid biosynthetic process		*	**			*				
	RNA metabolism										
0006351	Transcription, DNA-templated						*	*			
0006355	Regulation of transcription							*			
0051252	Regulation of RNA metabolic process							*			
	Photosynthesis										
0015979	Photosynthesis									*	*
0019684	Photosynthesis, light reaction									*	
0009773	Photosynthetic electron transport in photosystem I									*	
	Transport										
0008643	Carbohydrate transport			**							
0006855	Drug transmembrane transport			*							
0015893	Drug transport			*							
0055085	Transmembrane transport			**							
