# Supplementary material for: Brevicoryne brassicae aphids interfere with transcriptome responses of Arabidopsis thaliana to feeding by Plutella xylostella caterpillars in a density-dependent manner
Source: Oecologia. 2016 Oct 22;183(1):107–20. doi: 10.1007/s00442-016-3758-3 (PMC5239811; doi:10.1007/s00442-016-3758-3)
Supplement: Supplementary file 5 — Supplementary material 5 (PDF 365 kb) [file 442_2016_3758_MOESM5_ESM.pdf]

Brevicoryne brassicae aphids interfere with the whole-genome transcriptional responses of *Arabidopsis thaliana* to feeding by *Plutella xylostella* caterpillars in a density-dependent manner

Oecologia

Anneke Kroes, Colette Broekgaarden, Marcos Castellanos Uribe, Sean May, Joop JA van Loon, Marcel Dicke
Wageningen University, annekekroes@hotmail.com

ESM 5A. Annotation for genes up- or down-regulated based on fold-change ratios compared to control treatments within each cluster in response to single *P. xylostella* and dual *P. xylostella* and *B. brassicae* at low or high density at 24 h.

Cluster	Probe-ID	AGI	Protein/Description	Gene
1	13448215	AT3G12145	polygalacturonase inhibitory protein-like protein	FLR1
	13369126	AT1G17380	protein TIFY 11A	JAZ5
	13351851	AT1G53885		
	13351875	AT1G53885		
	13374940	AT1G32640	Basic helix-loop-helix (bHLH) DNA-binding family protein	MYC2
	13530600	AT5G24770	Vegetative storage protein 2	VSP2
	13402643	AT2G39030	Acyl-CoA N-acyltransferases (NAT) superfamily protein, response to JA stimuli	NATA1
	13472475	AT4G23600	Coronatine induced 3	CORI3
	13474404	AT4G27860	vacuolar iron transporter-like protein	
	13520416	AT5G67080	mitogen-activated protein kinase kinase kinase 19	MAPKKK19
	13379315	AT1G54020	GDSL esterase/lipase	
	13531726	AT5G28237	tryptophan synthase beta chain	
	13376396	AT1G45145	thioredoxin H5	TRX5
	13351005	AT1G51760	IAA-amino acid hydrolase ILR1-like 4	IAR3
	13450509	AT3G17330		ECT6
	13354462	AT1G62660	beta-fructofuranosidase	
	13396195	AT2G23010	serine carboxypeptidase-like 9	SCPL9
	13424055	AT3G04140	ankyrin repeat-containing protein	
	13486048	AT4G17470	palmitoyl-protein thioesterase	
	13469598	AT4G16590	cellulose synthase-like A01	CSLA01
	13353943	AT1G61120	terpene synthase 04	TPS04
	13412564	AT2G24850	Probable aminotransferase TAT3	TAT3

	13341372	AT1G17420	lipoxygenase 3	LOX3
	13483986	ATCSLA15	putative mannan synthase 15	ATCSLA15
	13358339	AT1G72520	PLAT/LH2 domain-containing lipoxygenase family protein	LOX4
	13500911	AT5G13220	protein TIFY 9	JAZ10
	13438530	AT3G52360		
	13355103	AT1G64500	glutaredoxin-like protein	
	13482365	AT4G08870	putative arginase	
	13378857	AT1G52890	NAC domain-containing protein 19	NAC019
	13484924	AT4G15210	beta-amylase 5	BAM5
	13522131	AT5G04950	Nicotianamine synthase 1	NAS1
2	13538661	AT5G52390	PAR1 protein	
	13391402	AT2G03933	defensin-like protein 59	
3	13386828	AT1G73325	Kunitz family trypsin and protease inhibitor protein	
	13434803	AT3G31051		
	13413731	AT2G27310	F-box protein	
	13492711	AT3G47730	homeobox protein ATH1	ATH1
	13520271	AT5G66740		
	13439813	AT3G55566		
	13479091	AT4G38310	galactosyl transferase GMA12/MNN10 family protein	
	13471623	AT4G21366	protein kinase family protein	
	13514353	AT5G53200	Transcription factor TRY	TRY
	13419757	AT2G41250	haloacid dehalogenase-like hydrolase domain-containing protein	
	13503787	AT5G19970		
	13408967	AT2G15020		
	13378483	AT1G52000	jacalin-like lectin domain-containing protein	
	13422367	AT2G47180	galactinol synthase 1	GolS1
	13437659	AT3G50280	uncharacterized acetyltransferase	
	13356558	AT1G68238		
	13453308	AT3G24460	Serinc-domain containing serine and sphingolipid biosynthesis protein	
	13496475	AT5G02230	haloacid dehalogenase-like hydrolase domain-containing protein	
	13501560	AT5G14760	L-aspartate oxidase	AO
	13542430	AT5G62140		
	13471834	AT4G21760	beta-glucosidase 47	BGLU47

13395385	AT2G21320	B-box zinc finger-like protein	
13339532	AT1G13080	cytochrome P450 71B2	CYP71B2
13383205	AT1G64780	ammonium transporter 1;2	AMT1;2
13380121	AT1G55960	putative polyketide cyclase/dehydrase and lipid transport-like protein	
13426086	AT3G09450		
13493685	AT4G35025		
13543280	AT5G64170	dentin sialophosphoprotein-like protein	
13485055	AT4G15550	indole-3-acetate beta-D-glucosyltransferase	IAGLU
13349716	AT1G48720		
13541350	AT5G59340	WUSCHEL-related homeobox 2	WOX2
13381126	AT1G59920	MADS-box family protein	
13435988	AT3G46130	transcription factor MYB48	MYB48

13339973	AT1G14250	GDA1/CD39 nucleoside phosphatase family protein	
13467396	AT4G11320	putative cysteine proteinase	
13530607	AT5G24780	Vegetative storage protein 1	VSP1
13357574	AT1G70700	protein TIFY 7	JAZ9
13450164	AT3G16470	Jacalin-related lecting 35	JR1
13351211	AT1G52410	TSK-associating protein 1	TSA1
13418960	AT2G39330	Jacalin-related lectin 23	JAL23
13404550	AT2G43530	defensin-like protein 194	
13498037	AT5G05600	xidoreductase, 2OG-Fe(II) oxygenase family protein	
13408088	AT2G06050	12-oxophytodienoate reductase 3	OPR3
13338866	AT1G11580	bifunctional pectinesterase 18/rRNA N-glycosylase	PMEPCRA
13530354	AT5G24420	6-phosphogluconolactonase 5	PGL5
13342166	AT1G19180	protein TIFY 10A	JAZ1, TIFY10A
13382114	AT1G61890	MATE efflux family protein	
13444660	AT3G04720	Hevein-like protein	PR4
13396571	AT2G24210	myrcene/ocimene synthase, involved in monoterpene (C10) biosynthesis	TPS10
13476200	AT4G31800	WRKY transcription factor 18	WRKY18
13402751	AT2G39420	alpha/beta-hydrolase domain-containing protein	
13386407	AT1G72450	protein TIFY 11B	JAZ6
13387476	AT1G74950	Protein TIFY 10B	TIFY10B
13460558	AT3G55970	Jasmonate-regulated protein JRG21	JRG21

13370075	AT1G19570	dehydroascorbate reductase	DHAR1
13378498	AT1G52040	myrosinase-binding protein 1	MBP1
13383834	AT1G66100	thionin	
13491180	AT4G29700	alkaline-phosphatase-like protein	
13452680	AT3G22740	Homocysteine S-methyltransferase 3	HMT3
13433248	AT3G25770	Allene oxide cyclase 2, chloroplastic	AOC2
13541003	AT5G58670	Phosphoinositide phospholipase C 1	PLC1
13545106	ATMG00110	cytochrome c biogenesis orf206	ccb206
13491711	AT4G30740		
13392226	AT2G07768	Cytochrome C assembly protein	
13413371	AT2G26740	soluble epoxide hydrolase	SEH
13414799	AT2G30250	Probable WRKY transcription factor 25	WRKY25
13344795	AT1G26730	phosphate transporter PHO1-7	
13540555	AT5G57550	xyloglucan:xyloglucosyl transferase	XTH25
13522407	AT5G05730	Anthranilate synthase component I-1, chloroplastic	ASA1
13440117	AT3G56200	transmembrane amino acid transporter-like protein	
13498809	AT5G07440	glutamate dehydrogenase 2	GDH2
13425044	AT3G06500	putative neutral invertase	
13534610	AT5G42650	Allene oxide synthase, chloroplastic	AOS
13519141	AT5G64260	protein EXORDIUM like 2	EXL2
13493404	AT4G34230	cinnamyl alcohol dehydrogenase 5, involved in response to wounding	CAD5
13522950	AT5G07010	sulfotransferase 2A	ST2A
13471894	AT4G21865		
13373610	AT1G29330	ER lumen protein retaining receptor	ERD2
13443544	AT3G02140	UPF0737 protein AFP4	TMAC2
13479362	AT4G39030	Enhanced disease susceptibility 5	EDS5
13521368	AT5G02940		
13527674	AT5G18020	SAUR-like auxin-responsive protein	
13351195	AT1G52400	beta glucosidase 18	BGLU18
13434249	AT3G28220	TRAF-like family protein	
13382362	AT1G62510	bifunctional inhibitor/lipid-transfer protein/seed storage 2S albumin-like protein	
13370131	AT1G19670	chlorophyllase 1	CLH1
13417017	AT2G34930	disease resistance-like protein/LRR domain-containing protein	

13404563	AT2G43550	defensin-like protein 197	
13371915	AT1G24070	putative mannan synthase 10	CSLA10
13505062	AT5G23820	MD-2-related lipid recognition domain-containing protein	
13400810	AT2G34810	FAD-binding and BBE domain-containing protein	
13433243	AT3G25760	Allene oxide cyclase 1, chloroplastic	AOC1
13404541	AT2G43510	defensin-like protein 195	TI1
13536497	AT5G47220	Ethylene-responsive transcription factor 2	ERF2
13517407	AT5G60300	L-TYPE LECTIN RECEPTOR KINASE I.9, involved in Phytophthora resistance	LecRK-1.9
13480976	AT4G02520	glutathione S-transferase PM24	GSTF2
13336673	AT1G06640	1-aminocyclopropane-1-carboxylate oxidase-2	
13503924	AT5G20230	blue-copper-binding protein, involved in (defense) response to wounding and fungus	BCB
13483621	AT4G12490	bifunctional inhibitor/lipid-transfer protein/seed storage 2S albumin-like protein	
13491495	AT4G30270	Xyloglucan endotransglucosylase/hydrolase protein 24	XTH24
13375843	AT1G36370	serine hydroxymethyltransferase 7	SHM7
13499587	AT5G09440	protein exordium like 4	EXL4
13467637	AT4G11890	protein kinase family protein	
13449744	AT3G15356	lectin-like protein	
13468928	AT4G15233	BC transporter G family member 42	
13393289	AT2G15960		
13370766	AT1G21130	O-methyltransferase-like protein	
13456715	AT3G46320	histone H4	
13412050	AT2G23600	acetone-cyanohydrin lyase	ACL
13451377	AT3G19553	Amino acid permease family protein	
13441605	AT3G60130	beta glucosidase 16	BGLU16
13505091	AT5G23860	Tubulin beta-8 chain	TUB8
13466519	AT4G08950	Phosphate-responsive 1 family protein	EXO
13392255	AT2G07785	NADH dehydrogenase I subunit 1	
13376292	AT1G44350	IAA-leucine resistant (ILR)-like gene 6	ILL6
13463327	AT3G62540	pentatricopeptide repeat-containing protein	
13472868	AT4G24350	phosphorylase family protein	
13498516	AT5G06870	Polygalacturonase inhibitor 2, involved in plant defense response, induced by MeJA	PGIP2
13355915	AT1G66760	MATE efflux family protein	
13393081	AT2G15090	3-ketoacyl-CoA synthase 8	KCS8

13387939	AT1G76090	24-methylenesterol C-methyltransferase 3	SMT3
13381231	AT1G60140	TREHALOSE PHOSPHATE SYNTHASE 10, enzyme involved in trehalose biosynthesis	TPS10
13411448	AT2G21660	COLD, CIRCADIAN RHYTHM, AND RNA BINDING 2, involved in innate immune responses	CCR2
13445533	AT3G06435		
13440012	AT3G55880	Alpha/beta hydrolase related protein	
13450223	AT3G16565	putative nucleotide binding protein	
13409382	AT2G16660	putative nodulin protein	
13516261	AT5G57655	xylose isomerase	
13337662	AT1G08890	sugar transporter ERD6-like 1	
13454669	AT3G27960	tetratricopeptide repeat domain-containing protein	
13503938	AT5G20250	Involved in the synthesis of raffinose, a major soluble carbohydrate in seeds	DIN10
13416526	AT2G33830	dormancy/auxin associated protein	
13351642	AT1G53430	Leucine-rich repeat transmembrane protein kinase	
13463143	AT3G62150	ABC transporter B family member 21	PGP21
13509621	AT5G42420	Nucleotide-sugar transporter family protein	
13378592	AT1G52200	PLAC8 family protein	
13397214	AT2G25930	Protein EARLY FLOWERING 3	ELF3
13457078	AT3G47340	Asparagine synthetase [glutamine-hydrolyzing]	ASN1
13475813	AT4G31000	Calmodulin-binding protein	
13424520	AT3G05220	heavy-metal-associated domain-containing protein	
13455353	AT3G30775	EARLY RESPONSIVE TO DEHYDRATION 5, defense response to bacterium	ERD5
13404135	AT2G42580	TETRATRICOPETIDE-REPEAT THIOREDOXIN-LIKE 3, involved in auxin signalling	TTL3
13433466	AT3G26200	cytochrome P450 71B22	CYP71B22
13545250	ATMG00160	cytochrome c oxidase subunit 2	cox2
13402290	AT2G38310	Abscisic acid (ABA) receptor PYL4	PYL4
13455581	AT3G42628	phosphoenolpyruvate carboxylase-related protein	
13468117	AT4G13340	leucine-rich repeat extensin-like protein 3	
13337711	AT1G08920	sugar transporter ERD6-like 3	ESL1
13523183	AT5G07620	protein kinase family protein	
13416588	AT2G33990	IQ-domain 9 protein	iqd9
13338220	AT1G10070	Branched-chain-amino-acid aminotransferase, chloroplastic	BCAT-2
13540599	AT5G57630	CBL-interacting serine/threonine-protein kinase 21	CIPK21
13515927	AT5G56870	Beta-galactosidase 4	BGAL4

13479173	AT4G38470	ACT-like protein tyrosine kinase family protein	
13449404	AT3G14310	Pectin methyl esterase 3, cell wall modification	PME3
13336964	AT1G07420	Methylsterol monooxygenase 2-1	SMO2-1
13386364	AT1G72416	chaperone DnaJ-domain containing protein	
13450196	AT3G16530	legume lectin-like protein	
13511600	AT5G47240	nudix hydrolase homolog 8, involved in response to wounding	NUDT8
13453505	AT3G24982	receptor like protein 40	RLP40
13461246	AT3G57520	Probable aquaporin SIP2-1	SIP2
13535388	AT5G44420	PLANT DEFENSIN 1.2	PDF1.2
13340261	AT1G15125	S-adenosyl-L-methionine-dependent methyltransferase domain-containing protein	
13486518	AT4G18440	adenylosuccinate lyase	
13397254	AT2G26020	PLANT DEFENSIN 1.2B	PDF1.2b
13412028	AT2G23560	Methyl esterase 7, involved in MeSA hydrolysis	MES7
13370802	AT1G21310	Extensin-3, structural constituent of cell wall	EXT3
13544741	AT5G67480	BTB and TAZ domain protein, involved in response to auxin, gibberellin, JA and SA	BT4
13454697	AT3G28120		
13371333	AT1G22570	putative peptide/nitrate transporter	
13484659	AT4G14680	3'-phosphoadenosine 5'-phosphosulfate synthase	APS3
13437483	AT3G49620	2-oxoglutarate-Fe(II)-dependent oxygenase domain-containing protein	DIN11
13356853	AT1G69040	ACT DOMAIN REPEAT 4, involved in response to cytokinin	ACR4
13494433	AT4G36670	putative polyol transporter 6	
13452877	AT3G23550	mate efflux domain-containing protein	
13517784	AT5G61160	anthocyanin 5-aromatic acyltransferase 1	AACT1
13492817	AT4G33150	lysine-ketoglutarate reductase/saccharopine dehydrogenase bifunctional enzyme	
13510327	AT5G44130	Fasciclin-like arabinogalactan-protein 13	FLA13
13514432	AT5G53410		
13343602	AT1G22882	Galactose-binding protein	
13476018	AT4G31354		
13392616	AT2G12905		
13389138	AT1G78850	D-mannose binding lectin protein with Apple-like carbohydrate-binding domain	
13437802	AT3G50700	indeterminate-domain 2 protein	IDD2
13401770	AT2G37025	Protein TRF-like 8	TRFL8
13523421	AT5G08150	suppressor of phytochrome b 5	SOB5

13344936	AT1G27020		
13530385	AT5G24470	two-component response regulator-like APRR5	PRR5
13350405	AT1G50460	hexokinase	HKL1
13538799	AT5G52882	putative ATP binding protein	
13538343	AT5G51550	protein EXORDIUM like 3	EXL3
13476805	AT4G32790	Exostosin family protein	
13527814	AT5G18380	40S ribosomal protein S16-3	
13476424	AT4G32285	putative clathrin assembly protein	
13373290	AT1G28330	dormancy-associated protein-like 1	DYL1
13410034	AT2G18340	Homeobox protein 24	HB24
13441274	AT3G59350	protein kinase family protein	
13460154	AT3G54880		
13421972	AT2G46330	Arabinogalactan peptide 16	AGP16
13392212	AT2G07672		
13545118	AT2G07727	cytochrome b	
13497869	AT5G05440	Abscisic acid (ABA) receptor PYL5	PYL5
13539982	AT5G56250	protein hapless 8	HAP8
13545140	AT2G07835		
13403960	AT2G42040		
13387975	AT1G76160	SKU5 similar 5 protein	sks5
13343614	AT1G22890		
13514548	AT5G53550	Metal-nicotianamine transporter YSL3	YSL3
13337731	AT1G08930	Sugar transporter ERD6	ERD6
13501351	AT5G14120	major facilitator protein	
13338744	AT1G11260	Sugar transport protein 1	STP1
13543434	AT5G64570		XYL4
13399175	AT2G31010	protein kinase domain-containing protein	
13543872	AT5G65660	hydroxyproline-rich glycoprotein family protein	
13487754	AT4G21870	heat shock protein class V 15.4	
13410499	AT2G19800	Inositol oxygenase 2	MIOX2
13436648	AT3G47800	aldose 1-epimerase	
13354674	AT1G63330	pentatricopeptide repeat-containing protein	
13371319	AT1G22530	Patellin-2	PATL2

	13497749	AT5G05140	Transcription elongation factor (TFIIS) family protein	
	13398059	AT2G28120	major facilitator protein	
	13398991	AT2G30600	BTB/POZ domain-containing protein	
	13392233	AT2G07678		
	13430753	AT3G19930	Sugar transport protein 4	STP4
	13362424	AT1G02660	alpha/beta-hydrolase domain-containing protein	
	13389129	AT1G78830	curculin-like (mannose-binding) lectin-like protein	
	13536625	AT5G47610	RING-H2 finger protein	ATL79
	13399351	AT2G31380	Salt tolerance-like protein	STH
	13408378	AT2G07820		
	13393304	AT2G16019		
	13446320	AT3G07850	galacturan 1,4-alpha-galacturonidase	
	13400962	AT2G35208		
	13334650	AT1G02074		
	13453371	AT3G24516		
	13545721	AT2G13422		
	13483523	AT4G12220		
	13458748	AT3G51642		
	13395740	AT2G22055	protein RALF-like 15	RALFL15
	13373972	AT1G30060	COP1-interacting protein-like protein	
	13468983	AT4G15350	cytochrome P450, family 705, subfamily A, polypeptide 2	CYP705A2
	13433910	AT3G27400	pectate lyase	
	13431605	AT3G21930	putative cysteine-rich repeat secretory protein 19	CRRSP19
6	13456185	AT3G44990	encodes a xyloglucan endotransglycosylase, involved in cell wall organization	XTR8
	13500637	AT5G12110	Elongation factor 1-beta 1	
	13381404	AT1G60590	Pectin lyase-like protein	
	13419467	AT2G40610	Expansin-A8	EXPA8
	13463007	AT3G61826		
	13375237	AT1G33607	putative defensin-like protein 26	
	13531035	AT5G25870		
	13395268	AT2G21010	calcium-dependent lipid-binding domain	
	13414291	AT2G28755	UDP-D-glucuronate carboxy-lyase-related protein	
	13409181	AT2G16120	polyol/monosaccharide transporter 1	PMT1

13453682	AT3G25650	S-phase kinase-associated protein 1	SK15
13524672	AT5G11027		
13495917	AT4G39986		
13397273	AT2G26120	glycine-rich protein	
13342911	AT1G21245	leucine-rich repeat receptor-like protein kinase	
13460452	AT3G55573		
13482388	AT4G08967		
13456144	AT3G44784		
