# Supplementary material for: Brevicoryne brassicae aphids interfere with transcriptome responses of Arabidopsis thaliana to feeding by Plutella xylostella caterpillars in a density-dependent manner
Source: Oecologia. 2016 Oct 22;183(1):107–20. doi: 10.1007/s00442-016-3758-3 (PMC5239811; doi:10.1007/s00442-016-3758-3)
Supplement: Supplementary file 6 — Supplementary material 6 (PDF 536 kb) [file 442_2016_3758_MOESM6_ESM.pdf]

Brevicoryne brassicae aphids interfere with the whole-genome transcriptional responses of *Arabidopsis thaliana* to feeding by *Plutella xylostella* caterpillars in a density-dependent manner

Oecologia

Anneke Kroes, Colette Broekgaarden, Marcos Castellanos Uribe, Sean May, Joop JA van Loon, Marcel Dicke
Wageningen University, annekekroes@hotmail.com

ESM 5B. Annotation for genes up- or down-regulated based on fold-change ratios compared to control treatments within each cluster in response to single *P. xylostella* and dual *P. xylostella* and *B. brassicae* at low or high density at 48 h.

Cluster	Probe-ID	AGI	Protein/Description	Gene
	13457959	AT3G49670	receptor-like kinase BAM2	BAM2
	13364821	AT1G07705	CCR4-NOT transcription complex subunit 2	
	13493685	AT4G35025		
	13373355	AT1G28550	RAB GTPase-like protein A1I	RABA1i
	13486146	AT4G17585	aluminum activated malate transporter family protein	
1	13449491	AT3G14510	geranylgeranyl diphosphate synthase, type II	
	13375237	AT1G33607	putative defensin-like protein 26	
	13453371	AT3G24516		
	13545357	ATMG01030		
	13342911	AT1G21245	leucine-rich repeat receptor-like protein kinase	
	13425753	AT3G08520	60S ribosomal protein L41	
	13339973	AT1G14250	GDA1/CD39 nucleoside phosphatase family protein	
	13351195	AT1G52400	beta glucosidase 18	BGLU18
	13434249	AT3G28220	TRAF-like family protein	
	13382362	AT1G62510	bifunctional inhibitor/lipid-transfer protein/seed storage 2S albumin-like protein	
	13370131	AT1G19670	CORONATINE-INDUCED PROTEIN 1	CLH1
	13357574	AT1G70700	Protein TIFY 7	JAZ9
	13422367	AT2G47180	galactinol synthase 1	GolS1
	13450164	AT3G16470	Jacalin-related lecting 35	JR1
	13351211	AT1G52410	TSK-associating protein 1	TSA1
	13402643	AT2G39030	Acyl-CoA N-acyltransferases (NAT) superfamily protein, response to JA stimuli	NATA1
	13472475	AT4G23600	Coronatine induced 3	CORI3

13418960	AT2G39330	Jacalin-related lectin 23	JAL23
13404550	AT2G43530	defensin-like protein 194	
13449554	AT3G14770	nodulin MtN3-like protein	
13342166	AT1G19180	protein TIFY 10A	JAZ1
13438530	AT3G52360		
13356558	AT1G68238		
13505062	AT5G23820	MD-2-related lipid recognition domain-containing protein	
13444660	AT3G04720	Hevein-like protein, pathogenesis-related 4, induced by JA	PR4
13413731	AT2G27310	F-box protein	
13400810	AT2G34810	FAD-binding and BBE domain-containing protein	
13532568	AT5G36220	Cytochrome P450 81D1	CYP81D1
13387476	AT1G74950	Protein TIFY 10B	JAZ2
13385322	AT1G69360		
13435633	AT3G44860	farnesoic acid carboxyl-O-methyltransferase	FAMT
13399351	AT2G31380	Salt tolerance-like protein	STH
13450509	AT3G17330		
13540188	AT5G56850		
13439861	AT3G55630	DHFS-FPGS homolog D	DFD
13448560	AT3G12750	Zinc transporter 1	ZIP1
13452477	AT3G21870	cyclin-U1-1	CYCP2;1
13498516	AT5G06870	Polygalacturonase inhibitor 2, involved in plant defense response, induced by MeJA	PGIP2
13503555	AT5G19500	Tryptophan/tyrosine permease	
13408577	AT2G12461		
13538661	AT5G52390	PAR1 protein	
13433248	AT3G25770	Allene oxide cyclase 2, chloroplastic	AOC2
13374910	AT1G32520		
13402512	AT2G38780		
13334720	AT1G02110		
13429613	AT3G17640	leucine-rich repeat-containing protein	
13486518	AT4G18440	adenylosuccinate lyase	
13530918	AT5G25610	Dehydration-responsive protein RD22, induction by salt stress, ABA and dehydration	RD22
13522293	AT5G05340	peroxidase 52	
13489365	AT4G25290	DNA photolyase	

13502293	AT5G16540	Zinc finger CCCH domain-containing protein 57, DNA-binding	ZFN3
13364296	AT1G06690	NAD(P)-linked oxidoreductase-like protein	
13373203	AT1G28190		
13348790	AT1G44575	Chlorophyll A-B binding family protein	NPQ4
13520589	AT5G67560	ADP-ribosylation factor-like A1D	ARLA1D
13433381	AT3G26060	Peroxiredoxin Q, chloroplastic	ATPRX
13401284	AT2G35830		
13353308	AT1G58520	lipase/hydrolase	RXW8
13448738	AT3G13120	30S ribosomal protein S10	
13483795	AT4G12980	putative auxin-responsive protein	
13489281	AT4G25100	Superoxide dismutase [Fe], chloroplastic	FSD1
13415936	AT2G32640	Lycopene beta/epsilon cyclase protein	
13417861	AT2G36835		
13404755	AT2G44160	Methylenetetrahydrofolate reductase 2	MTHFR2
13376206	AT1G44000		
13538174	AT5G51110	transducin/WD40 domain-containing protein	
13367467	AT1G13440	Glyceraldehyde-3-phosphate dehydrogenase, key enzyme in glycolysis	GAPC2
13493533	AT4G34630		
13478656	AT4G37080		
13400476	AT2G34170		
13336673	AT1G06640	1-aminocyclopropane-1-carboxylate oxidase-2	
13426598	AT3G10760	myb family transcription factor	
13443754	AT3G02730	Thioredoxin F-type 1, chloroplastic	TRXF1
13348842	AT1G44835	YbaK/aminoacyl-tRNA synthetase-associated domain-containing protein	
13397254	AT2G26020		PDF1.2b
13460664	AT3G56160	Sodium bile acid symporter family protein	
13453709	AT3G25717	protein rotundifolia like 16	RTFL16
13500341	AT5G11450	Mog1/PsbP/DUF1795-like photosystem II reaction center PsbP family protein	PPD5
13530156	AT5G24120	RNA polymerase sigma factor	SIGE
13373610	AT1G29330	ER lumen protein retaining receptor	ERD2
13452637	AT3G22600	bifunctional inhibitor/lipid-transfer protein/seed storage 2S albumin-like protein	
13509573	AT5G42310	pentatricopeptide repeat-containing protein	
13427204	AT3G12320		

13460174	AT3G54900	Monothiol glutaredoxin-S14, chloroplastic	CXIP1
13447654	AT3G10940	dual-specificity protein-like phosphatase 3	
13382220	AT1G62250		
13398908	AT2G30150	UDP-glucoronosyl/UDP-glucosyl transferase family protein	
13350204	AT1G49975		
13362633	AT1G03055		
13422760	AT3G01210	RNA recognition motif-containing protein	
13431920	AT3G22620	bifunctional inhibitor/lipid-transfer protein/seed storage 2S albumin-like protein	
13337524	AT1G08650	Phosphoenolpyruvate carboxylase kinase 1	PPCK1
13444774	AT3G05030	Sodium/hydrogen exchanger 2	NHX2
13446087	AT3G07300	translation initiation factor eIF-2B beta subunit	
13453585	AT3G25410	putative sodium-bile acid cotransporter	
13379948	AT1G55670	Photosystem I reaction center subunit V, chloroplastic	PSAG
13405943	AT2G46790	two-component response regulator-like APRR9	PRR9
13402772	AT2G39470	PsbP-like protein 2, chloroplastic	PPL2
13537235	AT5G48930	hydroxycinnamoyl-CoA shikimate/quinate hydroxycinnamoyl transferase, involved in response to wounding	HCT
13434201	AT3G28130	nodulin MtN21 /EamA-like transporter protein	
13425411	AT3G07350		
13535113	AT5G43870		
13406739	AT2G01290	ribose-5-phosphate isomerase 2	RPI2
13423490	AT3G02830	zinc finger CCCH domain-containing protein 33	ZFN1
13370697	AT1G20950	putative pyrophosphate-dependent phosphofructokinase alpha subunit	
13533250	AT5G38430	Ribulose bisphosphate carboxylase (small chain) family protein	RBCS1B
13471571	AT4G21280	Oxygen-evolving enhancer protein 3-1, required for photosystem II assembly/stability	PSBQA
13498318	AT5G06290	2-Cys peroxiredoxin BAS1-like, chloroplastic	2-Cys
13536344	AT5G46800	mitochondrial carnitine/acylcarnitine carrier-like protein	BOU
13386129	AT1G71710	endonuclease/exonuclease/phosphatase domain-containing protein	
13510502	AT5G44572		
13454697	AT3G28120		
13522906	AT5G06860	Polygalacturonase inhibitor 1	PGIP1
13350511	AT1G50732		
13500532	AT5G11790	protein N-MYC downregulated-like 2	NDL2

13471775	AT4G21670	RNA polymerase II C-terminal domain phosphatase-like 1, involved in ABA signalling and response to wounding	CPL1
13539841	AT5G55710		ATTIC20-V
13419687	AT2G41010	Calmodulin-binding protein that functions as a negative regulator of osmotic stress tolerance + regulation of SA metabolic process	CAMBP25
13400712	AT2G34620	transcription termination factor-like protein	
13537205	AT5G48850	tetratricopeptide repeat domain-containing protein	ATSDI1
13443363	AT3G01550	phosphoenolpyruvate (pep)/phosphate translocator 2	PPT2
13501057	AT5G13510	Ribosomal protein L10 family protein	
13509561	AT5G42270	VARIEGATED 1, PSII associated light-harvesting complex II catabolic process	VAR1
13338612	AT1G10960	Ferredoxin-1, chloroplastic	FD1
13360235	AT1G77490	L-ascorbate peroxidase T, chloroplastic	TAPX
13515291	AT5G55220	trigger factor type chaperone family protein	
13516003	AT5G57030	Lycopene epsilon cyclase, chloroplastic	LUT2
13386165	AT1G71810	aarF domain-containing kinase	
13356287	AT1G67730	Very-long-chain 3-oxoacyl-CoA reductase 1, this protein is involved in the pathway fatty acid biosynthesis	KCR1
13429028	AT3G16260	tRNAse Z4	TRZ4
13540856	AT5G58300	Methylesterase 18, JA metabolic process	MES18
13417425	AT2G35930	U-box domain-containing protein 23	PUB23
13352904	AT1G56430	nicotianamine synthase	NAS4
13493027	AT4G33470	Histone deacetylase 14	hda14
13430702	AT3G19800		
13336011	AT1G05200	Glutamate receptor 3.4, involved in response to wounding	GLR3.4
13528828	AT5G20700		
13512961	AT5G50100	putative thiol-disulfide oxidoreductase DCC	
13519470	AT5G64940	putative ABC transporter	ATH13
13476781	AT4G32770	Tocopherol cyclase, chloroplastic	VTE1
13397081	AT2G25605		
13540849	AT5G58260	oxidoreductase	
13477107	AT4G33580	beta carbonic anhydrase 5	BCA5
13413501	AT2G26930	4-diphosphocytidyl-2-C-methyl-D-erythritol kinase, chloroplastic, isopentenyl diphosphate biosynthetic process	CDPMEK

13440708	AT3G57810	Cysteine proteinases-like protein	
13542012	AT5G61130	glucan endo-1,3-beta-glucosidase-like protein 2	PDCB1
13472628	AT4G23940	FtsH extracellular protease	
13419402	AT2G40370	laccase 5	LAC5
13539851	AT5G55740	pentatricopeptide repeat-containing protein	CRR21
13421104	AT2G44090	ankyrin repeat-containing protein	
13503420	AT5G19290	esterase/lipase/thioesterase family protein	
13413721	AT2G27290		
13407995	AT2G05620	Protein PROTON GRADIENT REGULATION 5, chloroplastic	PGR5
13358555	AT1G73060	low PSII accumulation 3 protein	LPA3
13337337	AT1G08390		
13405917	AT2G46710	Rho GTPase activating protein with PAK-box/P21-Rho-binding domain	
13399746	AT2G32295	EXS (ERD1/XPR1/SYG1) domain-containing protein	
13396588	AT2G24280	alpha/beta-hydrolase domain-containing protein	
13536190	AT5G46290	3-oxoacyl-[acyl-carrier-protein] synthase I	KAS I
13355396	AT1G65230		
13407183	AT2G02500	2-C-methyl-D-erythritol 4-phosphate cytidylyltransferase, chloroplastic, isopentenyl diphosphate biosynthetic process	ISPD
13493399	AT4G34220	receptor protein kinase-like protein	
13528015	AT5G18700	Protein kinase family protein with ARM repeat domain	RUK
13414889	AT2G30390	Ferrochelatase-2, chloroplastic	FC2
13520508	AT5G67330	Metal transporter Nramp4	NRAMP4
13434151	AT3G28040	probably inactive leucine-rich repeat receptor-like protein kinase	
13388394	AT1G77090	PsbP domain-containing protein 4	
13346348	AT1G30520	acyl-activating enzyme 14	AAE14
13504132	AT5G20935		
13474876	AT4G28730	glutaredoxin-C5	
13464742	AT4G02100	Heat shock protein DnaJ with tetratricopeptide repeat	
13496582	AT5G02450	60S ribosomal protein L36-3	
13358536	AT1G72970	protein HOTHEAD	HTH
13463455	AT3G62880	translocase Oep16	ATOEP16-4
13450449	AT3G17170	Translation elongation factor EF1B/ribosomal protein S6 family protein	RFC3
13492719	AT4G33010	glycine dehydrogenase [decarboxylating] 2	GLDP1

13477446	AT4G34290	SWIB/MDM2 domain-containing protein	
13368864	AT1G16730		UP6
13370145	AT1G19700	BEL1-like homeodomain 10	BEL10
13372331	AT1G25440	zinc finger protein CONSTANS-LIKE 16	
13388306	AT1G76930	Encodes an extensin gene, involved in response to ABA, JA, SA and wounding	EXT4
13469756	AT4G16980	arabinogalactan family protein	
13445933	AT3G07090	PPPDE putative thiol peptidase family protein	
13534454	AT5G42110		
13485963	AT4G17230	Scarecrow-like protein 13	SCL13
13456801	AT3G46550	fasciclin-like arabinogalactan protein 4	SOS5
13490579	AT4G27820	beta glucosidase 9	BGLU9
13542685	AT5G62840	phosphoglycerate mutase-like protein	
13397176	AT2G25840	tryptophanyl-tRNA synthetase	OVA4
13352563	AT1G55480	protein containing PDZ domain, a K-box domain, and a TPR region, involved in photosynthesis	ZKT
13386428	AT1G72500		
13531184	AT5G26230		
13421979	AT2G46340	protein SUPPRESSOR OF PHYA-105 1	SPA1
13475215	AT4G29400		
13493158	AT4G33760	aspartyl-tRNA synthetase	
13473203	AT4G25050	acyl carrier protein 4	ACP4
13543253	AT5G64120	peroxidase 71	
13508753	AT5G40500		
13499587	AT5G09440	protein exordium like 4	EXL4
13494433	AT4G36670	putative polyol transporter 6	
13470679	AT4G19100		
13502561	AT5G17170	rubredoxin family protein	ENH1
13387934	AT1G76080	thioredoxin-like protein CDSP32	CDSP32
13446898	AT3G09410	putative pectinacetylesterase	
13355531	AT1G65490		
13517992	AT5G61670		
13523004	AT5G07180	LRR receptor-like serine/threonine-protein kinase ERL2	ERL2
13498909	AT5G07690	myb domain protein 29	MYB29
13465432	AT4G03635		

13385301	AT1G69295	plasmodesmata callose-binding protein 4	PDCB4
13502865	AT5G18080	SAUR-like auxin-responsive protein	
13460363	AT3G55330	PsbP-like protein 1	PPL1
13373323	AT1G28400		
13516378	AT5G57930	APO protein 2	APO2
13526682	AT5G15740	O-fucosyltransferase family protein	
13463447	AT3G62870	60S ribosomal protein L7a-2	
13359343	AT1G75210	HAD-superfamily hydrolase, subfamily IG, 5'-nucleotidase	
13447167	AT3G10050	threonine dehydratase biosynthetic	OMR1
13480918	AT4G02420	concanavalin A-like lectin kinase-like protein	
13400731	AT2G34655		
13438719	AT3G52960	peroxiredoxin-2E	
13485126	AT4G15810	GTP binding protein	
13480813	AT4G02075	protein pitchoun 1	PIT1
13473038	AT4G24570	dicarboxylate carrier 2	DIC2
13386414	AT1G72480	lung seven transmembrane receptor-like protein	
13545583	AT4G32600	C3H4 type zinc finger protein	
13504183	AT5G21100	L-ascorbate oxidase	
13425812	AT3G08680	putative inactive receptor kinase	
13530667	AT5G24890		
13536080	AT5G46160	large subunit ribosomal protein L14	
13396722	AT2G24592		
13457018	AT3G47070		
13336879	AT1G07180	alternative NAD(P)H dehydrogenase 1	NDA1
13379873	AT1G55530	RING/U-box domain-containing protein	
13469984	AT4G17560	50S ribosomal protein L19-1	
13389583	AT1G79550	Phosphoglycerate kinase, involved in glycolytic process	PGK
13449744	AT3G15356	lectin-like protein	
13528418	AT5G19850	hydrolase, alpha/beta fold family protein	
13355470	AT1G65380	receptor-like protein CLAVATA2	CLV2
13476018	AT4G31354		
13431845	AT3G22420	Serine/threonine-protein kinase WNK2	WNK2
13514219	AT5G52970	thylakoid lumenal protein 2	

13401680	AT2G36830	aquaporin TIP1-1	GAMMA-TIP
13367225	AT1G12900	glyceraldehyde 3-phosphate dehydrogenase A subunit 2	GAPA-2
13544835	ATCG00210	cytochrome b6/f complex subunit N	petN
13531174	AT5G26220	ChaC-like family protein	
13458629	AT3G51330	aspartyl protease family protein	
13484431	AT4G14240	CBS and DUF21 domain-containing protein	
13478204	AT4G35880	aspartyl protease family protein	
13392616	AT2G12905		
13465848	AT4G04925		
13413589	AT2G27040	argonaute 4	AGO4
13427403	AT3G12930	Lojap-related protein	
13399415	AT2G31610	40S ribosomal protein S3-1	
13345095	AT1G27340	F-box only protein 6	
13540270	AT5G56990		
13429390	AT3G17070	peroxidase 29	
13503094	AT5G18670	inactive beta-amylase 9	BMY3
13405400	AT2G45530	RING/U-box domain-containing protein	
13535223	AT5G44090	protein phosphatase 2 (formerly 2A), regulatory subunit B"	
13467520	AT4G11630	ribosomal protein L19	
13413371	AT2G26740	soluble epoxide hydrolase	SEH
13367202	AT1G12820	Protein AUXIN SIGNALING F-BOX 3	AFB3
13408998	AT2G15290	translocon at inner membrane of chloroplasts 21	TIC21
13397436	AT2G26500	putative cytochrome b6f complex subunit	
13513630	AT5G51560	leucine-rich repeat protein kinase-like protein	
13528178	AT5G19260		
13399897	AT2G32540	cellulose synthase-like protein B4	CSLB04
13454110	AT3G26910	hydroxyproline-rich glycoprotein family protein	
13415422	AT2G31440	gamma-secretase subunit APH1-like protein	
13449877	AT3G15630		
13486584	AT4G18670	leucine-rich repeat extensin-like protein 5	
13349836	AT1G49032		
13441841	AT3G60620	cytidinediphosphate diacylglycerol synthase 5	CDS5
13438427	AT3G52155	Phosphoglycerate mutase family protein	

13505475	AT5G25040	major facilitator protein	
13521038	AT5G01820	CBL-interacting serine/threonine-protein kinase 14	SR1
13389869	AT1G79920	Heat shock protein 70	
13498512	AT5G06860	Polygalacturonase inhibitor 1	PGIP1
13517163	AT5G59690	histone H4	
13358502	AT1G72930	toll/interleukin-1 receptor-like protein	TIR
13428254	AT3G14720	mitogen-activated protein kinase 19	MPK19
13493131	AT4G33680	LL-diaminopimelate aminotransferase	AGD2
13346141	AT1G30230	elongation factor 1-delta 1	
13421630	AT2G45520		
13420890	AT2G43620	chitinase-like protein	
13365404	AT1G09020	sucrose nonfermenting 4-like protein	SNF4
13545211	AT2G07749	Mitovirus RNA-dependent RNA polymerase	
13418127	AT2G37340	arginine/serine-rich zinc knuckle-containing protein 33	RSZ33
13462903	AT3G61650	Tubulin gamma-1 chain	TUBG1
13338472	AT1G10657		
13455581	AT3G42628	phosphoenolpyruvate carboxylase-related protein	
13417937	AT2G36885		
13482544	AT4G09670	uncharacterized oxidoreductase	
13428751	AT3G15640	cytochrome c oxidase subunit Vb	
13446640	AT3G08943	armadillo/beta-catenin-like repeat-containing protein	
13452100	AT3G21190	O-fucosyltransferase family protein	
13463128	AT3G62120	prolyl-tRNA synthetase	
13490383	AT4G27450	aluminum induced protein with YGL and LRDR motifs	
13544968	ATCG00220	photosystem II protein M	psbM
13470305	AT4G18300	Trimeric LpxA-like enzyme	
13523358	AT5G08040	mitochondrial import receptor subunit TOM5-like protein	TOM5
13382993	AT1G64040	serine/threonine-protein phosphatase PP1 isozyme 3	TOPP3
13452401	AT3G21670	major facilitator protein	
13544882	ATCG00690	photosystem II protein T	psbT
13517236	AT5G59890	Actin-depolymerizing factor 4	ADF4
13471363	AT4G20840	FAD-binding and BBE domain-containing protein	
13545250	ATMG00160	cytochrome c oxidase subunit 2	cox2

13519988	AT5G65990	Transmembrane amino acid transporter family protein	
13466659	AT4G09200	SPla/RYanodine receptor (SPRY) domain-containing protein	
13395532	AT2G21580	40S ribosomal protein S25-2	
13363290	AT1G04630	GRIM-19 protein	MEE4
13418529	AT2G38300	myb-like HTH transcriptional regulator-like protein	
13539269	AT5G54170	Lipid-binding START domain-containing protein	
13392212	AT2G07672		
13444562	AT3G04500	RNA recognition motif-containing protein	
13453844	AT3G26220	cytochrome P450 71B3	CYP71B3
13438484	AT3G52290	protein IQ-domain 3	IQD3
13345851	AT1G29510	SAUR-like auxin-respionsive protein family	SAUR68
13443749	AT3G02700	NC domain-containing protein	
13545118	AT2G07727	cytochrome b	
13480156	AT4G00620	Amino acid dehydrogenase family protein	
13439087	AT3G53870	40S ribosomal protein S3-2	
13466766	AT4G09630		
13415538	AT2G31670	Stress responsive alpha-beta barrel domain protein	
13390604	AT2G01850	xyloglucan:xyloglucosyl transferase	EXGT-A3
13335374	AT1G03610		
13534578	AT5G42530		
13380460	AT1G56580		
13349578	AT1G48440	protein B-cell receptor-associated 31-like protein	
13450030	AT3G15970	NUP50 protein	
13434746	AT3G30380	esterase/lipase domain-containing protein	
13419256	AT2G40000	HS1 PRO-1 2-like protein	HSPRO2
13544124	AT5G66120	putative 3-dehydroquinate synthase	
13545128	AT2G07774		
13545190	AT2G07774		
13340042	AT1G14370	protein kinase 2A	APK2A
13532169	AT5G35220	Peptidase M50 family protein	EGY1
13391844	AT2G05440	glycine-rich protein 9	GRP9
13542220	AT5G61590	ethylene-responsive transcription factor ERF107	

13408065	AT2G05940	Encodes a receptor-like cytoplasmic kinase that phosphorylates the host target RIN4, leading to the activation of a plant innate immune receptor RPM1	RIPK
13361537	AT1G80440	F-box/kelch-repeat protein	
13448857	AT3G13275		
13515550	AT5G55970	RING/U-box domain-containing protein	
13530480	AT5G24660	response to low sulfur 2	LSU2
13390271	AT1G80920	chaperone protein dnaJ 8	J8
13517686	AT5G60890	myb domain protein 34, modulates expression of ASA1, a key point of control in the tryptophan pathway. Involved in defence response to insect, JA, indole glucosinolate biosynthetic process	MYB34
13412683	AT2G25200		
13545285	ATMG00060	NADH dehydrogenase subunit 5	nad5
13467396	AT4G11320	putative cysteine proteinase	
13530607	AT5G24780	Vegetative storage protein 1	VSP1
13486048	AT4G17470	palmitoyl-protein thioesterase	
13353943	AT1G61120	terpene synthase 04	TPS04
13351851	AT1G53885		
13351875	AT1G53885		
13417017	AT2G34930	disease resistance-like protein/LRR domain-containing protein	
13376292	AT1G44350	IAA-leucine resistant (ILR)-like gene 6	ILL6
13358339	AT1G72520	lipoxygenase 4	LOX4
13382114	AT1G61890	MATE efflux family protein	
13433243	AT3G25760	Allene oxide cyclase 1, chloroplastic	AOC1
13404541	AT2G43510	defensin-like protein 195	TI1
13402751	AT2G39420	alpha/beta-hydrolase domain-containing protein	
13460558	AT3G55970	jasmonate-regulated protein JRG21	JRG21
13542308	AT5G61890	ethylene-responsive transcription factor ERF114	
13452680	AT3G22740	Homocysteine S-methyltransferase 3	HMT3
13362335	AT1G02340	transcription factor HFR1	HFR1
13480976	AT4G02520	glutathione S-transferase PM24	GSTF2
13503924	AT5G20230	blue-copper-binding protein, involved in (defense) response to wounding and fungus	BCB
13483621	AT4G12490	bifunctional inhibitor/lipid-transfer protein/seed storage 2S albumin-like protein	
13529974	AT5G23660	MTN3-like protein	MTN3

13535388	AT5G44420		PDF1.2
13461246	AT3G57520	putative galactinol--sucrose galactosyltransferase 2	SIP2
13435464	AT3G44550	Fatty acid reductase 5 (alcohol-forming fatty acyl-CoA reductases), generates fatty alcohols in wound-induced leaf tissue	FAR5
13476550	AT4G32480		
13443480	AT3G01970	WRKY DNA-binding protein 45	WRKY45
13544741	AT5G67480	BTB and TAZ domain protein, involved in response to SA, JA and wounding	BT4
13540555	AT5G57550	xyloglucan:xyloglucosyl transferase	XTH25
13428695	AT3G15500	Encodes an ATAF-like NAC-domain transcription factor	NAC3
13502069	AT2G02800	stress-induced protein KIN2	KIN2
13511600	AT5G47240	nudix hydrolase homolog 8, involved in response to wounding	NUDT8
13453505	AT3G24982	receptor like protein 40	RLP40
13413305	AT2G26560	phospholipase A 2A	PLA2A
13512671	AT5G49520	putative WRKY transcription factor 48	WRKY48
13478398	AT4G36410	Probable ubiquitin carrier protein E2 17	UBC17
13362949	AT1G03850	monothiol glutaredoxin-S13	
13457466	AT3G48360	BTB AND TAZ DOMAIN PROTEIN 2, involved in different responses such as to ABA, auxin, JA, SA and wounding	BT2
13417478	AT2G36080	B3 domain-containing protein	
13494509	AT4G36850	PQ-loop repeat family protein / transmembrane family protein	
13414799	AT2G30250	Probable WRKY transcription factor 25	WRKY25
13483625	AT4G12500	bifunctional inhibitor/lipid-transfer protein/seed storage 2S albumin-like protein	
13478745	AT4G37260	myb domain protein 73	MYB73
13515927	AT5G56870	beta-galactosidase 4	BGAL4
13540561	AT5G57560	Encodes a cell wall modifying enzyme, involved in response to auxin	TCH4
13461382	AT3G57930		
13389138	AT1G78850	D-mannose binding lectin protein with Apple-like carbohydrate-binding domain	
13523421	AT5G08150	suppressor of phytochrome b 5	SOB5
13396584	AT2G24240	BTB/POZ domain with WD40/YVTN repeat-containing protein	
13498597	AT5G07100	WRKY DNA-binding protein 26	WRKY26
13473709	AT4G26150	putative GATA transcription factor 22	CGA1
13469187	AT4G15670	monothiol glutaredoxin-S7	
13467419	AT4G11360	RING-H2 zinc finger protein RHA1b	RHA1B

13430665	AT3G19680		
13542795	AT5G63160	BTB and TAZ domain protein 1	BT1
13469234	AT4G15800	protein ralf-like 33	RALFL33
13449274	AT3G14090	exocyst complex component 7	EXO70D3
13389129	AT1G78830	curculin-like (mannose-binding) lectin-like protein	
13497869	AT5G05440	abscisic acid receptor PYL5	PYL5
13469199	AT4G15700	monothiol glutaredoxin-S3	
13457078	AT3G47340	Asparagine synthetase [glutamine-hydrolyzing]	ASN1
13535298	AT5G44260	zinc finger CCCH domain-containing protein 61	
13398059	AT2G28120	major facilitator protein	
13378592	AT1G52200	PLAC8 family protein	
13392255	AT2G07785	NADH dehydrogenase I subunit 1	
13366036	AT1G10140		
13501351	AT5G14120	major facilitator protein	
13338744	AT1G11260	Sugar transport protein 1	STP1
13338784	AT1G11380	PLAC8 family protein	
13503938	AT5G20250	putative galactinol--sucrose galactosyltransferase 6	DIN10
13486904	AT4G19420	putative Pectinacetylesterase	
13343195	AT1G21910	Encodes a member of the DREB subfamily A-5 of ERF/AP2 transcription factor family, involved in ET signalling pathway, response to JA and SA	DREB26
13417706	AT2G36630	Sulfite exporter TauE/SafE family protein	
13355103	AT1G64500	glutaredoxin-like protein	
13536625	AT5G47610	RING-H2 finger protein ATL79	
13492711	AT3G47730	homeobox protein ATH1	ATH1
13450367	AT3G16910	acyl-activating enzyme 7	AAE7
13412084	AT2G23670	homolog of Synechocystis YCF37	YCF37
13515419	AT5G55620		
13453308	AT3G24460	Serinc-domain containing serine and sphingolipid biosynthesis protein	
13509362	AT5G41900	hydrolase, alpha/beta fold family protein	
13456185	AT3G44990	xyloglucan:xyloglucosyl transferase	XTR8
13496475	AT5G02230	haloacid dehalogenase-like hydrolase domain-containing protein	
13374919	AT1G32550	Encodes FdC2, a ferredoxin protein	FDC2
13351979	AT1G54200		

13535385	AT5G44410	FAD-binding and BBE domain-containing protein	
13405356	AT2G45340	putative leucine-rich repeat transmembrane protein kinase	
13542430	AT5G62140		
13500637	AT5G12110	Elongation factor 1-beta 1	
13387187	AT1G74180	receptor like protein 14	RLP14
13471834	AT4G21760	beta-glucosidase 47	BGLU47
13381404	AT1G60590	Pectin lyase-like protein	
13503787	AT5G19970		
13462022	AT3G59400	tetrapyrrole-binding protein	GUN4
13408967	AT2G15020		
13443014	AT3G01060		
13336822	AT1G07010	calcineurin-like metallo-phosphoesterase-like protein	
13521349	AT5G02830	pentatricopeptide repeat-containing protein	
13358147	AT1G72030	Acyl-CoA N-acyltransferases-like protein	
13417922	AT2G36870	xyloglucan:xyloglucosyl transferase	XTH32
13406354	AT2G47590	photolyase/blue-light receptor 2	PHR2
13369792	AT1G18650	plasmodesmata callose-binding protein 3	PDCB3
13526218	AT5G14640	Shaggy-related protein kinase epsilon	SK13
13542506	AT5G62430	Dof zinc finger protein DOF5.5	CDF1
13409748	AT2G17695		
13419467	AT2G40610	Expansin-A8	EXPA8
13395385	AT2G21320	B-box zinc finger-like protein	
13514998	AT5G54585		
13339532	AT1G13080	cytochrome P450 71B2	CYP71B2
13467786	AT4G12390	pectin methylesterase inhibitor 1	PME1
13484612	AT4G14480	protein kinase family protein	
13457134	AT3G47500	Dof zinc finger protein DOF3.3	CDF3
13352731	AT1G55910	zinc transporter 11	ZIP11
13444908	AT3G05180	GDSL esterase/lipase	
13437511	AT3G49670	receptor-like kinase BAM2	BAM2
13406279	AT2G47490	NAD+ transporter 1	NDT1
13383205	AT1G64780	ammonium transporter 1;2	AMT1;2
13510120	AT5G43630	zinc knuckle (CCHC-type) family protein	TZP

13400176	AT2G33250		
13426301	AT3G10060	FKBP-like peptidyl-prolyl cis-trans isomerase-like protein	
13468983	AT4G15350	cytochrome P450, family 705, subfamily A, polypeptide 2	CYP705A2
13463804	AT4G00050	transcription factor UNE10	UNE10
13414849	AT2G30320	putative tRNA pseudouridine synthase	
13403215	AT2G40400		
13334797	AT1G02205	CER1 protein	CER1
13395116	AT2G20605	Plant thionin family protein	
13411980	AT2G23430	Cyclin-dependent kinase inhibitor 1	ICK1
13354850	AT1G63850	TIR-NBS-LRR class disease resistance protein	
13442913	AT3G63370	pentatricopeptide repeat-containing protein	OTP86
13444258	AT3G03770	leucine-rich repeat protein kinase-like protein	
13428988	AT3G16175	thioredoxin family protein	
13387161	AT1G74070	cyclophilin-like peptidyl-prolyl cis-trans isomerase-like protein	
13514353	AT5G53200	Transcription factor TRY	TRY
13419757	AT2G41250	haloacid dehalogenase-like hydrolase domain-containing protein	
13495716	AT4G39460	S-adenosylmethionine carrier 1	SAMC1
13418251	AT2G37640	expansin-A3	EXP3
13387209	AT1G74260	phosphoribosylformylglycinamidine synthase	PUR4
13380121	AT1G55960	putative polyketide cyclase/dehydrase and lipid transport-like protein	
13414988	AT2G30520	Root phototropism protein 2	RPT2
13540671	AT5G57780		
13357877	AT1G71500	Rieske (2Fe-2S) domain-containing protein	
13406291	AT2G47500	putative kinesin heavy chain	
13401303	AT2G35860	fasciclin-like arabinogalactan protein 16	FLA16
13337838	AT5G45720	AAA-type ATPase family protein	
13370341	AT5G45720	AAA-type ATPase family protein	
13494665	AT5G45720	AAA-type ATPase family protein	
13531589	AT5G45720	AAA-type ATPase family protein	
13544460	AT5G45720	AAA-type ATPase family protein	
13340414	AT1G15510	pentatricopeptide repeat-containing protein	ECB2
13451016	AT3G18750	putative serine/threonine-protein kinase WNK6	WNK6
13371792	AT1G23740	Alkenal/one oxidoreductase, helps to maintain the photosynthetic process	AOR

13500309	AT5G11380	1-deoxy-D-xylulose 5-phosphate synthase 3	DXPS3
13367754	AT1G14225		
13361019	AT1G79270		
13406443	AT2G47844		
13381161	AT1G60000	RNA recognition motif-containing protein	
13520345	AT5G66820		
13388299	AT1G76880	putative trihelix DNA-binding protein	
13521663	AT5G03760	glucomannan 4-beta-mannosyltransferase 9	ATCSLA09
13395165	AT2G20750	expansin B1	EXPB1
13355217	AT1G64680		
13494881	AT4G37800	xyloglucan:xyloglucosyl transferase	XTH7
13473876	AT4G26520	Aldolase superfamily protein, involved in glycolytic process	ATFBA7
13402227	AT2G38140	30S ribosomal protein S31	PSRP4
13340615	AT1G15950	cinnamoyl coa reductase 1	CCR1
13339925	AT1G14150	Encodes a subunit of the NAD(P)H dehydrogenase complex	PQL2
13417929	AT2G36880	S-adenosylmethionine synthase 3	MAT3
13525307	AT5G12470		
13434078	AT3G27830	50S ribosomal protein L12-1	RPL12-A
13426086	AT3G09450		
13464412	AT4G01460	transcription factor bHLH57	
13369699	AT1G18360	alpha/beta-hydrolase domain-containing protein	
13447998	AT3G11750	Dihydroneopterin aldolase	FOLB1
13482463	AT4G09350	chaperone DnaJ-domain containing protein	
13366928	AT1G12000	pyrophosphate--fructose-6-phosphate 1-phosphotransferase	
13490082	AT4G26850	Encodes a novel protein involved in ascorbate biosynthesis, defense response by callose deposition in cell wall and JA	VTC2
13435123	AT3G43540		
13346985	AT1G32160		
13385338	AT1G69440	argonaute-like protein	AGO7
13364633	AT1G07350	RNA recognition motif-containing protein	
13495851	AT4G39800	inositol-3-phosphate synthase isozyme 1	MIPS1
13429756	AT3G17840	putative inactive receptor kinase RLK902	RLK902
13504425	AT5G22340		

13373761	AT1G29720	Leucine-rich repeat transmembrane protein kinase	
13382398	AT1G62710	vacuolar-processing enzyme beta-isozyme	BETA-VPE
13465479	AT4G04020	FIBRILLIN, involved in abscisic acid-mediated photoprotection	FIB
13436413	AT3G47430	peroxisomal membrane protein 11B	PEX11B
13529473	AT5G22620	phosphoglycerate/bisphosphoglycerate mutase family protein	
13387889	AT3G05520	F-actin-capping protein subunit alpha	
13458322	AT3G05520	F-actin-capping protein subunit alpha	
13531188	AT5G26330	plastocyanin-like domain-containing protein / putative mavicyanin	
13385435	AT1G69730	wall-associated receptor kinase-like 9	
13475697	AT4G30825	pentatricopeptide repeat-containing protein	
13456759	AT3G46450	SEC14 cytosolic factor family protein / phosphoglyceride transfer family protein	
13369517	AT1G18060		
13431526	AT3G21560	sinapate 1-glucosyltransferase	UGT84A2
13521035	AT5G01790		
13349820	AT1G49010	myb/SANT-like DNA-binding domain-containing protein	
13499340	AT5G08570	pyruvate kinase	
13396399	AT2G23670	HOMOLOG OF SYNECHOCYSTIS YCF37	YCF37
13452760	AT3G22980	elongation factor EF-2	
13448447	AT3G12580	heat shock protein 70-4	HSP70
13386911	AT1G73540	nudix hydrolase 21	NUDT21
13378982	AT1G53280	4-methyl-5(b-hydroxyethyl)-thiazole monophosphate biosynthesis	
13368108	AT1G14840	microtubule-associated proteins 70-4	MAP70-4
13400845	AT2G34925	protein CLAVATA3/ESR-related 42	CLE42
13473175	AT4G24972	tapetum determinant 1	TPD1
13390506	AT2G01590	chlororespiratory reduction 3	CRR3
13449344	AT3G14200	chaperone DnaJ-domain containing protein	
13361499	AT1G80320	2-oxoglutarate (2OG) and Fe(II)-dependent oxygenase-like protein	
13339015	AT1G11850		
13427672	AT3G13470	TCP-1/cpn60 chaperonin family protein	
13395859	AT2G22360	molecular chaperone DnaJ	
13352327	AT1G55020	Lipoxygenase 1	LOX1
13515028	AT5G54660	nuclear-enriched phloem companion cell 8 protein	
13355059	AT1G64390	endoglucanase 6	GH9C2

13544661	AT5G67280	receptor-like kinase	RLK
13446913	AT3G09440	protein heat shock protein 70-3	
13340379	AT1G15410	aspartate-glutamate racemase-like protein	
13402202	AT2G38080	laccase-4	IRX12
13533451	AT5G39110	germin-like protein subfamily 1 member 14	
13485055	AT4G15550	indole-3-acetate beta-D-glucosyltransferase	IAGLU
13465362	AT4G03500	ankyrin repeat-containing protein	
13530175	AT5G24150	Squalene monooxygenase 1,1	SQP1
13443420	AT3G01790	large subunit ribosomal protein L13	
13437797	AT3G05520	F-actin-capping protein subunit alpha	
13400668	AT2G34460	NAD(P)-binding Rossmann-fold-containing protein	
13460579	AT3G56040	UDP-glucose pyrophosphorylase 3	UGP3
13512653	AT5G49480	Ca2+-binding protein 1	CP1
13458515	AT3G51010		
13460373	AT3G55360	enoyl reductase	CER10
13357339	AT1G70250	putative receptor serine/threonine kinase	
13522070	AT5G04790		
13545617	AT3G50250		
13335301	AT1G03360	exosome complex component RRP4	RRP4
13456794	AT3G46540	ENTH/VHS family protein	
13342418	AT1G19960		
13530185	AT5G24155	FAD/NAD(P)-binding oxidoreductase family protein	
13545631	AT4G19270		
13403683	AT2G41310	two-component response regulator ARR8	RR3
13479975	AT4G00270	NA-binding storekeeper protein-related transcriptional regulator	
13361503	AT1G80370	cyclin-A2-4	CYCA2;4
13509650	AT5G42480	chaperone DnaJ-domain containing protein	ARC6
13521711	AT5G03850	40S ribosomal protein S28-1	
13543072	AT5G63790	NAC domain-containing protein 102	NAC102
13458521	AT3G51030	Thioredoxin H-type 1	TRX1
13445024	AT3G05520	F-actin-capping protein subunit alpha	
13517400	AT5G60280	concanavalin A-like lectin kinase-like protein	
13398023	AT2G28000	Encodes chaperonin-60 alpha, a molecular chaperone involved in Rubisco folding	CPN60A

13359993	AT1G76880	putative trihelix DNA-binding protein	
13395740	AT2G22055	protein RALF-like 15	RALFL15
13358197	AT1G72130	putative peptide/nitrate transporter	
13519821	AT5G65683	C3H4 type zinc finger protein	
13341406	AT1G17470	developmentally regulated G-protein 1	DRG1
13455432	AT3G32180		
13337127	AT1G07890	L-ascorbate peroxidase 1, cytosolic	APX1
13378962	AT1G53240	Malate dehydrogenase 1, mitochondrial	mMDH1
13494761	AT4G37450	Lysine-rich arabinogalactan protein 18	AGP18
13520271	AT5G66740		
13527695	AT5G18150	Methyltransferase-related protein	
13460736	AT3G56340	40S ribosomal protein S26-3	
13335773	AT1G04501		
13357899	AT1G71680	Lysine histidine transporter-like 5	
13365497	AT1G09157		
13386746	AT1G73240		
13513711	AT5G51720	Iron-binding zinc finger CDGSH type domain-containing protein	
13510286	AT5G44030	cellulose synthase A catalytic subunit 4 [UDP-forming]	CESA4
13375245	AT1G33640		
13490760	AT4G28250	expansin B3	EXPB3
13508299	AT5G39080	HXXXD-type acyl-transferase-like protein	
13426731	AT3G11120	60S ribosomal protein L41	
13401383	AT2G36026	Ovate family protein	
13545711	AT1G32290		
13403136	AT2G40150	trichome birefringence-like 28 protein	TBL28
13472213	AT4G22930	dihydroorotase	PYR4
13459649	AT3G53890	40S ribosomal protein S21-1	
13343889	AT1G23710		
13350964	AT1G51700	Dof zinc finger protein DOF1.7	DOF1
13520861	AT5G01430	Got1/Sft2-like vescicle transport protein	
13538980	AT5G53205		
13371618	AT1G23340		
13494833	AT4G37610	BTB and TAZ domain protein 5	BT5

	13392313	AT2G07722		
	13394755	AT2G19750	40S ribosomal protein S30	
	13417394	AT2G35860	fasciclin-like arabinogalactan protein 16	FLA16
	13412564	AT2G24850	Probable aminotransferase TAT3	TAT3
	13474404	AT4G27860	vacuolar iron transporter-like protein	
	13498037	AT5G05600	oxidoreductase, 2OG-Fe(II) oxygenase family protein	
	13500911	AT5G13220	jasmonate-zim-domain protein 10	JAZ10, TIFY9
	13476200	AT4G31800	WRKY transcription factor 18	WRKY18
	13531409	AT5G27060	receptor like protein 53	RLP53
	13400703	AT2G34600	jasmonate-zim-domain protein 7	JAZ7, TIFY58
	13525220	AT5G12340		
	13353727	AT1G60110	jacalin-like lectin domain-containing protein	
	13341929	AT1G18710	myb domain protein 47	MYB47
	13508854	AT5G40690		
	13522950	AT5G07010	sulfotransferase 2A	ST2A
	13452269	AT3G21500	1-deoxy-D-xylulose 5-phosphate synthase 1	DXPS1
	13354187	AT1G61810	beta-glucosidase 45	BGLU45
	13347601	AT1G33960	protein AIG1	AIG1
	13360299	AT1G77640	ethylene-responsive transcription factor ERF013	
	13456089	AT3G44660	Putative histone deacetylase 10	hda10
	13517784	AT5G61160	anthocyanin 5-aromatic acyltransferase 1	AACT1
	13434307	AT3G28510	AAA-type ATPase family protein	
	13346398	AT1G30640	Protein kinase family protein	
	13439444	AT3G54730		
	13519067	AT5G64000	SAL2 phosphatase	SAL2
	13537386	AT5G49360	beta-xylosidase 1	BXL1
	13470228	AT4G18170	Probable WRKY transcription factor 28	WRKY28
	13338220	AT1G10070	Branched-chain-amino-acid aminotransferase, chloroplastic	BCAT-2
	13392523	AT2G11015		
	13378412	AT1G51890	putative leucine-rich repeat protein kinase	
5	13435953	AT3G45970	expansin-like A1	EXLA1
	13518330	AT5G62360	plant invertase/pectin methylesterase inhibitor domain-containing protein	
	13485325	AT4G16260	catalytic/ cation binding / hydrolase	

13353090	AT1G57630	Toll-Interleukin-Resistance domain-containing protein	
13481931	AT4G05330	putative ADP-ribosylation factor GTPase-activating protein AGD13	AGD13
13399125	AT2G30930		
13344936	AT1G27020		
13346118	AT1G30135	protein TIFY 5A	JAZ8
13531431	AT5G27238	self-incompatibility S1 family protein	
13483613	AT4G12470	azelaic acid induced 1	AZI1
13378927	AT1G53100	Core-2/I-branching beta-1,6-N-acetylglucosaminyltransferase-like protein	
13366223	AT1G10550	xyloglucan:xyloglucosyl transferase	XTH33
13357031	AT1G69520	S-adenosylmethionine-dependent methyltransferase domain-containing protein	
13530076	AT5G23980	ferric reduction oxidase 4	FRO4
13400156	AT2G33080	receptor like protein 28	RLP28
13334842	AT1G02380		
13451641	AT3G20180	putative copper transport protein	
13456858	AT3G46720	UDP-glycosyltransferase-like protein	
13532022	AT5G33300	chromosome-associated kinesin-like protein	
13383995	AT1G66520	methionyl-tRNA formyltransferase	pde194
13340483	AT1G15670	putative F-box/kelch-repeat protein	
13458642	AT3G51340	aspartyl protease family protein	
13478916	AT4G37770	1-aminocyclopropane-1-carboxylate synthase 8	ACS8
13518322	AT5G62340	plant invertase/pectin methylesterase inhibitor domain-containing protein	
13484817	AT4G14960	tubulin alpha-6 chain	TUA6
13371259	AT1G22400	cytokinin-O-glucosyltransferase 2	UGT85A1
13387986	AT1G76170	2-thiocytidine tRNA biosynthesis protein, TtcA	
13447329	AT3G10320	Glycosyltransferase family 61 protein	

13519397	AT5G64750	Ethylene-responsive transcription factor ABR1	ABR1
13376854	AT1G48145		
13498728	AT5G07310	Ethylene-responsive transcription factor ERF115	
13360431	AT1G77950	MADS-box	AGL67
13472127	AT4G22710	cytochrome P450, family 706, subfamily A, polypeptide 2	CYP706A2
13375475	AT1G34400		
13341228	AT1G17180	glutathione S-transferase TAU 25	GSTU25
13458258	AT3G50373		

13384345	AT1G67160	putative F-box protein	
13410166	AT2G18810		
13381058	AT1G59675	F-box protein	
13452831	AT3G23245		
13400463	AT2G34120	Cytochrome C oxidase polypeptide VIB family protein	
13483254	AT4G11653	RALF-like 29 protein	RALFL29
13355467	AT1G65360	Type 1 MADS-box gene that control female gametophyte development	AGL23
13529689	AT5G22890	C2H2 and C2HC zinc finger-containing protein	
13356561	AT1G68290	endonuclease 2	ENDO
13454993	AT3G28990		
13422348	AT2G47150	Rossmann-fold NAD(P)-binding domain-containing protein	
13412370	AT2G24430	NAC domain containing protein 38	NAC038
13535432	AT5G44540	Tapetum specific protein TAP35/TAP44	
13418987	AT2G39415	putative F-box protein	
13465882	AT4G05018		
13533781	AT5G40220	MADS-box	AGL43
13484419	AT4G14226		
13439798	AT3G55515	protein rotundifolia like 7	RTFL7
13388429	AT1G77160		
13375477	AT1G34419		
13457788	AT3G49340	putative cysteine proteinase	
13541253	AT5G59110	subtilisin-like serine protease-like protein	
13409491	AT2G16955		
13507373	AT5G35715	Cytochrome P450 71B8	CYP71B8
13455360	AT3G30778		
13410527	AT2G19900	malate dehydrogenase (oxaloacetate-decarboxylating)(NADP+)	NADP-ME1
13384025	AT1G66570	putative sucrose transport protein SUC7	SUC7
13335650	AT1G04180	YUCCA 9 protein	YUC9
13456472	AT3G45760	Nucleotidyltransferase family protein	
13483883	AT4G13230	late embryogenesis abundant domain-containing protein	
13392865	AT2G14206		
13533418	AT5G38900	DSBA oxidoreductase family protein	
13436223	AT3G46760	protein kinase family protein	

	13475335	AT4G29770		
6	13347647	AT1G34041		
	13482992	AT4G10870		
	13440207	AT3G56470	F-box protein	
	13482388	AT4G08967		
	13345840	AT1G29480		
	13376257	AT1G44130	aspartyl protease-like protein	
	13364501	AT1G07050	CCT motif family protein	
	13415224	AT2G31018		
	13407886	AT2G04870		
	13461735	AT3G58676		
	13405029	AT2G44690	Rac-like GTP-binding protein ARAC9	ARAC9
	13434283	AT3G28310		
	13414343	AT2G28850	cytochrome P450, family 710, subfamily A	CYP710A3
	13455347	AT3G30730		
	13414291	AT2G28755	UDP-D-glucuronate carboxy-lyase-related protein	
	13345894	AT1G29580		
	13518531	AT5G62780	chaperone DnaJ-domain containing protein	
	13494609	AT4G37060	PATATIN-like protein 5	PLP5
	13423901	AT3G03800	syntaxin 1B/2/3	SYP131
	13507130	AT5G34870	zinc knuckle (CCHC-type) family protein	
	13334831	AT1G02320		
	13457884	AT3G49551		
	13391255	AT2G03600	ureide permease 3	UPS3
	13375297	AT1G33870	putative avirulence-responsive protein	
	13537378	AT5G49350	glycine-rich protein	
	13509102	AT5G41200	MADS-box	AGL75
	13474983	AT4G28870		
	13355841	AT1G66553		
	13383722	AT1G65890	acyl activating enzyme 12	AAE12
	13376746	AT1G48010	plant invertase/pectin methylesterase inhibitor domain-containing protein	
	13543843	AT5G65533		
	13422999	AT3G01760	Lysine histidine transporter-like 4	

13467593	AT4G11760	defensin-like protein 151	LCR17
13492358	AT4G32208	heat shock protein 70 (Hsp 70) family protein	
13481987	AT4G05523		
13393304	AT2G16019		
13345520	AT1G28304		
13494653	AT4G37160	protein SKU5 similar 15	sks15
13342376	AT1G19830	SAUR-like auxin-respionsive protein family	SAUR54
13423348	AT3G02480	Late embryogenesis abundant protein (LEA) family protein	
13480550	AT4G01520	NAC domain containing protein 67	NAC067
13374694	AT1G31875		
13381126	AT1G59920	MADS-box family protein	
13469467	AT4G16165	carbohydrate-binding X8 domain-containing protein	
