# Supplementary material for: Brevicoryne brassicae aphids interfere with transcriptome responses of Arabidopsis thaliana to feeding by Plutella xylostella caterpillars in a density-dependent manner
Source: Oecologia. 2016 Oct 22;183(1):107–20. doi: 10.1007/s00442-016-3758-3 (PMC5239811; doi:10.1007/s00442-016-3758-3)
Supplement: Supplementary file 7 — Supplementary material 7 (PDF 657 kb) [file 442_2016_3758_MOESM7_ESM.pdf]

Brevicoryne brassicae aphids interfere with the whole-genome transcriptional responses of *Arabidopsis thaliana* to feeding by *Plutella xylostella* caterpillars in a density-dependent manner

Oecologia

Anneke Kroes, Colette Broekgaarden, Marcos Castellanos Uribe, Sean May, Joop JA van Loon, Marcel Dicke
Wageningen University, annekekroes@hotmail.com

ESM 5C. Annotation for interaction significant genes (two-way ANOVA) up- or down-regulated based on fold-change ratios compared to control treatments within each cluster in response to single *P. xylostella* and dual *P. xylostella* and *B. brassicae* at low or high density at both 24 and 48 h.

Cluster	Probe-ID	AGI	Protein/Description	Gene
	13447998	AT3G11750	Dihydroneopterin aldolase	FOLB1
	13401072	AT2G35500	Magnesium-ion binding, Probable inactive shikimate kinase like 2, chloroplastic	SKL2
	13443329	AT3G01510	Phosphoglucan phosphatase LSF1, chloroplastic	LSF1
	13417922	AT2G36870	xyloglucan:xyloglucosyl transferase	XTH32
	13400831	AT2G34860	Protein EMBRYO SAC DEVELOPMENT ARREST 3, chloroplastic	EDA3
	13450367	AT3G16910	acyl-activating enzyme 7	AAE7
	13462022	AT3G59400	tetrapyrrole-binding protein	GUN4
	13406354	AT2G47590	photolyase/blue-light receptor 2	PHR2
	13369792	AT1G18650	plasmodesmata callose-binding protein 3	PDCB3
	13414157	AT2G28470	beta-galactosidase 8	BGAL8
	13494881	AT4G37800	xyloglucan:xyloglucosyl transferase	XTH7
	13527997	AT5G18660	PALE-GREEN AND CHLOROPHYLL B REDUCED 2	PCB2
	13477286	AT4G34050	Probable caffeoyl-CoA O-methyltransferase	CCoAOMT1
	13443056	AT3G01120	cystathionine gamma-synthase	MTO1
	13468983	AT4G15350	cytochrome P450, family 705, subfamily A, polypeptide 2	CYP705A2
	13460579	AT3G56040	UDP-glucose pyrophosphorylase 3	UGP3
	13543072	AT5G63790	NAC domain-containing protein 102	NAC102
	13372600	AT1G26355	protein SPIRAL1-like1	SP1L1
	13387209	AT1G74260	phosphoribosylformylglycinamidine synthase	PUR4
	13339925	AT1G14150	Encodes a subunit of the NAD(P)H dehydrogenase complex	PQL2
	13507303	AT5G35490	mto 1 responding up 1	MRU1
	13448512	AT3G12680	zinc finger CCCH domain-containing protein 37	HUA1

13438484	AT3G52290	protein IQ-domain 3	IQD3
13450509	AT3G17330		ECT6
13530156	AT5G24120	RNA polymerase sigma factor	SIGE
13387934	AT1G76080	thioredoxin-like protein CDSP32	CDSP32
13336879	AT1G07180	alternative NAD(P)H dehydrogenase 1	NDA1
13389583	AT1G79550	Phosphoglycerate kinase, involved in glycolytic process	PGK
13367202	AT1G12820	Protein AUXIN SIGNALING F-BOX 3	AFB3
13405973	AT2G46830	Protein CIRCADIAN CLOCK ASSOCIATED 1	CCA1
13490082	AT4G26850	Encodes a novel protein involved in ascorbate biosynthesis, defense response by callose deposition in cell wall and JA	VTC2
13423605	AT3G03110	exportin 1B	XPO1B
13411980	AT2G23430	Cyclin-dependent kinase inhibitor 1	ICK1
13340615	AT1G15950	cinnamoyl coa reductase 1	CCR1
13356961	AT1G69390	bacterial MinE 1-like protein	MINE1
13361503	AT1G80370	cyclin-A2-4	CYCA2;4
13509650	AT5G42480	chaperone DnaJ-domain containing protein	ARC6
13439861	AT3G55630	DHFS-FPGS homolog D	DFD
13489281	AT4G25100	Superoxide dismutase [Fe], chloroplastic; Superoxide dismutase	FSD1
13460174	AT3G54900	Monothiol glutaredoxin-S14, chloroplastic	CXIP1
13495983	AT4G40060	homeobox-leucine zipper protein ATHB-16	HB16
13402772	AT2G39470	PsbP-like protein 2, chloroplastic	PPL2
13498318	AT5G06290	2-Cys peroxiredoxin BAS1-like, chloroplastic	2-Cys
13536344	AT5G46800	mitochondrial carnitine/acylcarnitine carrier-like protein	BOU
13338612	AT1G10960	Ferredoxin-1, chloroplastic	FD1
13346348	AT1G30520	acyl-activating enzyme 14	AAE14
13485418	AT4G16370	oligopeptide transporter	OPT3
13479147	AT4G38430	Rop guanine nucleotide exchange factor 1	ROPGEF1
13388974	AT5G20280	Sucrose-phosphate synthase 1, Plays a major role in photosynthetic sucrose synthesis	SPS1
13399351	AT2G31380	Salt tolerance-like protein	STH
13530918	AT5G25610	Dehydration-responsive protein RD22	RD22
13526218	AT5G14640	Shaggy-related protein kinase epsilon	SK13
13421886	AT2G46070	Mitogen-activated protein kinase 12	MPK12
13422824	AT3G01370	CRM family member 2	CFM2

13430817	AT3G20000	mitochondrial import receptor subunit TOM40-1	TOM40
13414988	AT2G30520	Root phototropism protein 2	RPT2
13361019	AT1G79270		ECT8
13385338	AT1G69440	argonaute-like protein	AGO7
13448560	AT3G12750	Zinc transporter 1	ZIP1
13520589	AT5G67560	ADP-ribosylation factor-like A1D	ARLA1D
13446521	AT3G22960	serine/threonine-protein kinase AtPK1/AtPK6	PK1
13397513	AT2G26670	heme oxygenase 1	TED4
13367467	AT1G13440	Glyceraldehyde-3-phosphate dehydrogenase, key enzyme in glycolysis	GAPC2
13453709	AT3G25717	protein rotundifolia like 16	RTFL16
13423490	AT3G02830	zinc finger CCCH domain-containing protein 33	ZFN1
13500532	AT5G11790	protein N-MYC downregulated-like 2	NDL2
13443363	AT3G01550	phosphoenolpyruvate (pep)/phosphate translocator 2	PPT2
13425053	AT3G06510	beta-glucosidase-like SFR2	SFR2
13408130	AT2G06520	photosystem II subunit X	PSBX
13338378	AT1G10510	leucine-rich repeats-ribonuclease inhibitor domain-containing protein	emb2004
13463699	AT3G63410	MPBQ/MSBQ methyltransferase	APG1
13407183	AT2G02500	2-C-methyl-D-erythritol 4-phosphate cytidylyltransferase, chloroplastic	ISPD
13361674	AT1G80770	putative GTP-binding protein	PDE318
13523004	AT5G07180	LRR receptor-like serine/threonine-protein kinase ERL2	ERL2
13498909	AT5G07690	myb domain protein 29, defence response to fungus	MYB29
13355545	AT1G65590	beta-hexosaminidase 3	HEXO3
13383786	AT1G65980	peroxiredoxin-2B	TPX1
13484233	AT4G13930	Serine hydroxymethyltransferase	SHM4
13375973	AT1G41830	SKU5 similar 6	SKS6
13394929	AT2G20260	Photosystem I reaction center subunit IV B, chloroplastic	PSAE-2
13407995	AT2G05620	Protein PROTON GRADIENT REGULATION 5, chloroplastic	PGR5
13421979	AT2G46340	protein SUPPRESSOR OF PHYA-105 1	SPA1
13395068	AT2G20570	GBF's pro-rich region-interacting factor 1	GPRI1
13440362	AT3G56940	COPPER RESPONSE DEFECT 1, involved in photosynthesis	CRD1
13463455	AT3G62880	translocase Oep16	ATOEP16-4
13466967	AT4G10120	Probable sucrose-phosphate synthase 4, plays a role in photosynthetic sucrose synthesis	ATSPS4F
13519470	AT5G64940	putative ABC transporter	ATH13

1

13378982	AT1G53280	4-methyl-5(b-hydroxyethyl)-thiazole monophosphate biosynthesis	
13384690	AT1G67785		
13374919	AT1G32550	Encodes FdC2, a ferredoxin protein	FDC2
13374726	AT1G32080		
13369699	AT1G18360	alpha/beta-hydrolase domain-containing protein	
13475409	AT4G30020	PA-domain containing subtilase family protein	
13417706	AT2G36630	Sulfite exporter TauE/SafE family protein	
13535385	AT5G44410	FAD-binding and BBE domain-containing protein	
13405356	AT2G45340	putative leucine-rich repeat transmembrane protein kinase	
13473882	AT4G26530	FRUCTOSE-BISPHOSPHATE ALDOLASE 5, involved in glycolysis	FBA5
13531860	AT5G28750	sec-independent protein translocase protein TatA	
13465157	AT4G02920		
13504272	AT5G21430	chaperone DnaJ-domain containing protein	
13400179	AT2G33255	Haloacid dehalogenase-like hydrolase	
13354850	AT1G63850	TIR-NBS-LRR class disease resistance protein	
13387161	AT1G74070	cyclophilin-like peptidyl-prolyl cis-trans isomerase-like protein	
13357877	AT1G71500	Rieske (2Fe-2S) domain-containing protein	
13346985	AT1G32160		
13536625	AT5G47610	RING-H2 finger protein ATL79	
13443014	AT3G01060		
13418745	AT2G38870	serine protease inhibitor, potato inhibitor I-type protein	
13460452	AT3G55573		
13358889	AT1G73870	zinc finger protein CONSTANS-LIKE 7	
13472159	AT4G22850	SNARE associated Golgi protein family	
13457018	AT3G47070		
13379873	AT1G55530	RING/U-box domain-containing protein	
13359455	AT1G75460	ATP-dependent protease La domain-containing protein	
13534578	AT5G42530		
13436188	AT3G46600	scarecrow-like protein 30	
13387889	AT3G05520	F-actin-capping protein subunit alpha	
13458322	AT3G05520	F-actin-capping protein subunit alpha	
13456759	AT3G46450	SEC14 cytosolic factor family protein / phosphoglyceride transfer family protein	
13369517	AT1G18060		

13437797	AT3G05520	F-actin-capping protein subunit alpha
13343889	AT1G23710	
13334720	AT1G02110	
13483795	AT4G12980	putative auxin-responsive protein
13449828	AT3G15570	Phototropic-responsive NPH3 family protein
13427204	AT3G12320	
13423435	AT3G02690	nodulin MtN21 /EamA-like transporter protein
13352931	AT1G56500	haloacid dehalogenase-like hydrolase domain-containing protein
13528828	AT5G20700	
13525489	AT5G13140	Pollen Ole e 1 allergen and extensin family protein
13396588	AT2G24280	alpha/beta-hydrolase domain-containing protein
13386873	AT1G73390	Endosomal targeting BRO1-like domain-containing protein
13362633	AT1G03055	
13337337	AT1G08390	
13457417	AT3G48200	
13410772	AT2G20250	
13358147	AT1G72030	Acyl-CoA N-acyltransferases-like protein
13452538	AT3G22210	
13363230	AT1G04530	tetratricopeptide repeat domain-containing protein
13337838	AT5G45720	AAA-type ATPase family protein
13370341	AT5G45720	AAA-type ATPase family protein
13494665	AT5G45720	AAA-type ATPase family protein
13531589	AT5G45720	AAA-type ATPase family protein
13544460	AT5G45720	AAA-type ATPase family protein
13520345	AT5G66820	
13355217	AT1G64680	
13435123	AT3G43540	
13504425	AT5G22340	
13385435	AT1G69730	wall-associated receptor kinase-like 9
13350737	AT1G51350	armadillo/beta-catenin-like repeats-containing protein
13540188	AT5G56850	
13408577	AT2G12461	
13429613	AT3G17640	leucine-rich repeat-containing protein

13489365	AT4G25290	DNA photolyase
13494746	AT4G37380	Tetratricopeptide repeat (TPR)-like superfamily protein
13401284	AT2G35830	
13448738	AT3G13120	30S ribosomal protein S10
13415936	AT2G32640	Lycopene beta/epsilon cyclase protein
13417861	AT2G36835	
13376206	AT1G44000	
13538174	AT5G51110	transducin/WD40 domain-containing protein
13467928	AT4G12830	hydrolase, alpha/beta fold family protein
13509573	AT5G42310	pentatricopeptide repeat-containing protein
13386129	AT1G71710	endonuclease/exonuclease/phosphatase domain-containing protein
13350511	AT1G50732	
13400712	AT2G34620	transcription termination factor-like protein
13427066	AT3G12010	
13540861	AT5G58330	lactate/malate dehydrogenase family protein
13423027	AT3G01810	
13523692	AT5G08720	
13359039	AT1G74160	
13430702	AT3G19800	
13510644	AT5G44870	TIR-NBS-LRR class disease resistance protein
13479049	AT4G38220	Peptidase M20/M25/M40 family protein
13430293	AT3G18890	Rossmann-fold NAD(P)-binding domain-containing protein
13493792	AT4G35270	RWP-RK domain-containing protein
13454176	AT3G26990	ENTH/VHS family protein
13489115	AT4G24780	putative pectate lyase 18
13542485	AT5G62300	40S ribosomal protein S20-1
13386428	AT1G72500	
13542205	AT5G61510	NADPH2:quinone reductase
13474890	AT4G28760	
13502865	AT5G18080	SAUR-like auxin-responsive protein
13476408	AT4G32240	
13502717	AT5G17560	BolA-like protein
13400731	AT2G34655	

	13465302	AT4G03260	Outer arm dynein light chain 1 protein	
	13530667	AT5G24890		
	13442858	AT3G63170	Chalcone-flavanone isomerase family protein	
	13429390	AT3G17070	peroxidase 29	
	13510883	AT5G45430	conserved peptide upstream open reading frame 24	
	13341359	AT1G17360		
	13451553	AT3G19895	RING/U-box-containing protein-like protein	
	13413132	AT2G26190	calmodulin-binding-like protein	
	13374910	AT1G32520		
	13422760	AT3G01210	RNA recognition motif-containing protein	
	13421104	AT2G44090	ankyrin repeat-containing protein	
	13496452	AT5G02180	Transmembrane amino acid transporter family protein	
	13454230	AT3G27050		
	13355531	AT1G65490		
	13432020	AT3G22970		
	13418072	AT2G37240	Thioredoxin-like protein	
	13397436	AT2G26500	putative cytochrome b6f complex subunit	
	13513630	AT5G51560	leucine-rich repeat protein kinase-like protein	
	13346141	AT1G30230	elongation factor 1-delta 1	
	13361537	AT1G80440	F-box/kelch-repeat protein	
	13515550	AT5G55970	RING/U-box domain-containing protein	
2	13439444	AT3G54730		
	13531431	AT5G27238	self-incompatibility S1 family protein	
	13378927	AT1G53100	Core-2/I-branching beta-1,6-N-acetylglucosaminyltransferase-like protein	
	13360299	AT1G77640	ethylene-responsive transcription factor ERF013	
	13434307	AT3G28510	AAA-type ATPase family protein	
	13474305	AT4G27654		
	13354187	AT1G61810	beta-glucosidase 45	BGLU45
	13392523	AT2G11015		
	13347601	AT1G33960	protein AIG1	AIG1
	13483635	AT4G12530	bifunctional inhibitor/lipid-transfer protein/seed storage 2S albumin-like protein	
	13451641	AT3G20180	putative copper transport protein	
	13518322	AT5G62340	plant invertase/pectin methylesterase inhibitor domain-containing protein	

13499340	AT5G08570	pyruvate kinase	
13335301	AT1G03360	exosome complex component RRP4	RRP4
13545363	ATMG01110		ArthMp097
13519988	AT5G65990	Transmembrane amino acid transporter family protein	
13368108	AT1G14840	microtubule-associated proteins 70-4	MAP70-4
13402202	AT2G38080	Laccase-4	IRX12
13520412	AT2G19045	protein ralf-like 34	RALFL34
13510286	AT5G44030	cellulose synthase A catalytic subunit 4 [UDP-forming]	CESA4
13472868	AT4G24350	phosphorylase family protein	
13340261	AT1G15125	S-adenosyl-L-methionine-dependent methyltransferase domain-containing protein	
13514432	AT5G53410		
13425812	AT3G08680	putative inactive receptor kinase	
13334609	AT1G01970	pentatricopeptide repeat-containing protein	
13427403	AT3G12930	Lojap-related protein	
13421630	AT2G45520		
13417937	AT2G36885		
13446640	AT3G08943	armadillo/beta-catenin-like repeat-containing protein	
13373638	AT1G29370	Kinase-related protein	
13464742	AT4G02100	Heat shock protein DnaJ with tetratricopeptide repeat	
13395532	AT2G21580	40S ribosomal protein S25-2	
13422367	AT2G47180	galactinol synthase 1	GolS1
13373034	AT1G27730	zinc finger protein STZ/ZAT10	STZ
13503023	AT5G18550	zinc finger CCCH domain-containing protein 58	
13448857	AT3G13275		
13400127	AT2G32990	endoglucanase 11	GH9B8
13459649	AT3G53890	40S ribosomal protein S21-1	
13538980	AT5G53205		
13494761	AT4G37450	Lysine-rich arabinogalactan protein 18	AGP18
13401383	AT2G36026	Ovate family protein	
13422565	AT2G47840		
13336462	AT3G23490	U5 small nuclear ribonucleoprotein component	MEE5
13382710	AT1G63470	AT hook motif DNA-binding family protein	
13400668	AT2G34460	NAD(P)-binding Rossmann-fold-containing protein	

13426731	AT3G11120	60S ribosomal protein L41	
13383058	AT1G64200	V-type proton ATPase subunit E3	VHA-E3
13519821	AT5G65683	C3H4 type zinc finger protein	
13508299	AT5G39080	HXXXD-type acyl-transferase-like protein	
13460373	AT3G55360	enoyl reductase	CER10
13358197	AT1G72130	putative peptide/nitrate transporter	
13341406	AT1G17470	developmentally regulated G-protein 1	DRG1
13337127	AT1G07890	L-ascorbate peroxidase 1, cytosolic	APX1
13460736	AT3G56340	40S ribosomal protein S26-3	
13475194	AT4G29350	profilin 2	PFN2
13371618	AT1G23340		
13472213	AT4G22930	dihydroorotase	PYR4
13394755	AT2G19750	40S ribosomal protein S30	
13507625	AT5G37020	Auxin response factor 8	ARF8
13352563	AT1G55480	protein containing PDZ domain, a K-box domain, and a TPR region, involved in photosynthesis	ZKT
13481730	AT4G04890	Homeobox-leucine zipper protein PROTODERMAL FACTOR 2	PDF2
13493027	AT4G33470	Histone deacetylase 14	hda14
13445203	AT3G05880	Hydrophobic protein RCI2A	RCI2A
13356853	AT1G69040	ACT DOMAIN REPEAT 4, involved in response to cytokinin	ACR4
13424371	AT3G04940	Cysteine synthase	CYSD1
13502387	AT5G16715	valyl-tRNA synthetase	EMB2247
13536190	AT5G46290	3-oxoacyl-[acyl-carrier-protein] synthase I	KAS
13450449	AT3G17170	Translation elongation factor EF1B/ribosomal protein S6 family protein	RFC3
13506402	AT5G27360	sugar transporter ERD6-like 18	SFP2
13397176	AT2G25840	tryptophanyl-tRNA synthetase	OVA4
13457302	AT3G47990	E3 ubiquitin-protein ligase SIS3	SIS3
13485377	AT4G16340	guanyl-nucleotide exchange factor / GTPase binding / GTP binding protein	SPK1
13443754	AT3G02730	Thioredoxin F-type 1, chloroplastic	TRXF1
13471775	AT4G21670	RNA polymerase II C-terminal domain phosphatase-like 1	CPL1
13509561	AT5G42270	VARIEGATED 1, PSII associated light-harvesting complex II catabolic process	VAR1
13356287	AT1G67730	Very-long-chain 3-oxoacyl-CoA reductase 1, this protein is involved in the pathway fatty acid biosynthesis	KCR1
13477107	AT4G33580	beta carbonic anhydrase 5	BCA5

13542012	AT5G61130	glucan endo-1,3-beta-glucosidase-like protein 2	PDCB1
13393237	AT2G15620	Ferredoxin--nitrite reductase	NIR1
13492719	AT4G33010	glycine dehydrogenase [decarboxylating] 2	GLDP1
13470328	AT4G18370	Encodes DEG5, involved in photosystem II repair	DEG5
13537326	AT5G49230	protein dehydration-INDUCED 19-7	HRB1
13526934	AT5G16390	biotin carboxyl carrier protein of acetyl-CoA carboxylase 1	CAC1
13460363	AT3G55330	PsbP-like protein 1	PPL1
13448084	AT4G08620	Sulfate transporter 1.1	HST
13401680	AT2G36830	aquaporin TIP1-1	GAMMA-TIP
13367225	AT1G12900	glyceraldehyde 3-phosphate dehydrogenase A subunit 2	GAPA-2
13366423	AT1G10870	ADP-ribosylation factor GTPase-activating protein AGD4	AGD4
13493558	AT4G34720	V-type H+-transporting ATPase 16kDa proteolipid subunit	AVA-P1
13348790	AT1G44575	Chlorophyll A-B binding family protein	NPQ4
13518456	AT5G62670	H(+)-ATPase 11	HA11
13471571	AT4G21280	Oxygen-evolving enhancer protein 3-1, required for photosystem II assembly/stability	PSBQA
13413501	AT2G26930	4-diphosphocytidyl-2-C-methyl-D-erythritol kinase, chloroplastic, isopentenyl diphosphate biosynthetic process	CDPMEK
13341450	AT1G17580	myosin 1	MYA1
13443316	AT3G01500	Carbonic anhydrase, chloroplastic	CA1
13537253	AT5G49030	isoleucyl-tRNA synthetase	OVA2
13387506	AT1G74970	30S ribosomal protein S9	RPS9
13447167	AT3G10050	threonine dehydratase biosynthetic	OMR1
13512110	AT5G48300	Glucose-1-phosphate adenylyltransferase small subunit, chloroplastic	ADG1
13349052	AT1G45474	Encodes a component of the light harvesting complex of photosystem I	LHCA5
13408998	AT2G15290	translocon at inner membrane of chloroplasts 21	TIC21
13391759	AT2G05170	Vacuolar protein-sorting-associated protein 11 homolog	VPS11
13416588	AT2G33990	IQ-domain 9 protein	iqd9
13507084	AT5G33320	Glucose-6-phosphate/phosphate translocator-like protein	CUE1
13407216	AT2G02710	Encodes a putative blue light receptor protein.	PLPB
13421972	AT2G46330	Arabinogalactan peptide 16	AGP16
13506305	AT5G27150	sodium/hydrogen exchanger 1	NHX1
13404755	AT2G44160	Methylenetetrahydrofolate reductase 2	MTHFR2
13448369	AT3G12400	Protein ELC	ELC

13510989	AT5G45680	FKBP-type peptidyl-prolyl cis-trans isomerase 3	FKBP13
13362866	AT1G03680	Thioredoxin M-type 1, chloroplastic	THM1
13413149	AT2G26250	3-ketoacyl-CoA synthase 10	KCS10
13379948	AT1G55670	Photosystem I reaction center subunit V, chloroplastic	PSAG
13450754	AT3G18110	pentatricopeptide repeat-containing protein	EMB1270
13537235	AT5G48930	hydroxycinnamoyl-CoA shikimate/quinate hydroxycinnamoyl transferase, involved in response to wounding	HCT
13480002	AT4G00370	putative anion transporter 2	ANTR2
13419207	AT2G39940	Coronatine-insensitive protein 1	COI1
13545577	AT2G20860	lipoic acid synthase 1	LIP1
13360235	AT1G77490	L-ascorbate peroxidase T, chloroplastic	TAPX
13516003	AT5G57030	Lycopene epsilon cyclase, chloroplastic	LUT2
13429028	AT3G16260	tRNAse Z4	TRZ4
13540856	AT5G58300	Methylesterase 18, JA metabolic process	MES18
13469780	AT4G17050	ureidoglycine aminohydrolase	UGLYAH
13476781	AT4G32770	Tocopherol cyclase, chloroplastic	VTE1
13442639	AT3G62410	CP12-2 is coordinately regulated by light with the photosynthetic GAPDH and PRK	CP12-2
13539851	AT5G55740	pentatricopeptide repeat-containing protein	CRR21
13537539	AT5G49730	ferric reduction oxidase 6	FRO6
13396355	AT2G23420	Nicotinate phosphoribosyltransferase 2	NAPRT2
13414889	AT2G30390	Ferrochelatase-2, chloroplastic	FC2
13541115	AT5G58870	cell division protease ftsH-9	ftsh9
13439196	AT3G54050	fructose-1,6-bisphosphatase	HCEF1
13456790	AT3G46530	disease resistance protein RPP13	RPP13
13514017	AT5G52520	prolyl-tRNA synthetase	OVA6
13516378	AT5G57930	APO protein 2	APO2
13529630	AT5G22800	alanyl-tRNA synthetase	EMB1030
13544835	ATCG00210	cytochrome b6/f complex subunit N	petN
13499720	AT5G09870	cellulose synthase A catalytic subunit 5 [UDP-forming]	CESA5
13508937	AT5G40870	putative uracil phosphoribosyltransferase	UK
13458420	AT3G50750	BES1/BZR1 homolog 1	BEH1
13496208	AT5G01530	Light harvesting complex photosystem II	LHCB4.1
13544968	ATCG00220	photosystem II protein M	psbM

13458310	AT3G50500	Serine/threonine-protein kinase SRK2D	SNRK2.2
13544882	ATCG00690	photosystem II protein T	psbT
13545250	ATMG00160	cytochrome c oxidase subunit 2	cox2
13390604	AT2G01850	xyloglucan:xyloglucosyl transferase	EXGT-A3
13495384	AT4G38740	Peptidyl-prolyl cis-trans isomerase CYP18-3	ROC1
13404653	AT2G43820	UDP-glucosyltransferase 74F2	UGT74F2
13340042	AT1G14370	protein kinase 2A	APK2A
13390271	AT1G80920	chaperone protein dnaJ 8	J8
13535388	AT5G44420	PLANT DEFENSIN 1.2	PDF1.2
13375843	AT1G36370	serine hydroxymethyltransferase 7	SHM7
13452680	AT3G22740	Homocysteine S-methyltransferase 3	HMT3
13469234	AT4G15800	protein ralf-like 33	RALFL33
13357574	AT1G70700	Protein TIFY 7	TIFY7, JAZ9
13444660	AT3G04720	Hevein-like protein	PR4
13387476	AT1G74950	Protein TIFY 10B	TIFY10B. JAZ[illegible]
13433248	AT3G25770	Allene oxide cyclase 2, chloroplastic	AOC2
13373610	AT1G29330	ER lumen protein retaining receptor	ERD2
13491495	AT4G30270	Xyloglucan endotransglucosylase/hydrolase protein 24	XTH24
13499587	AT5G09440	protein exordium like 4	EXL4
13493131	AT4G33680	LL-diaminopimelate aminotransferase	AGD2
13418127	AT2G37340	arginine/serine-rich zinc knuckle-containing protein 33	RSZ33
13505091	AT5G23860	Tubulin beta-8 chain	TUB8
13517236	AT5G59890	Actin-depolymerizing factor 4	ADF4
13541003	AT5G58670	Phosphoinositide phospholipase C 1	PLC1
13451587	AT3G20020	protein arginine N-methyltransferase 6	PRMT6
13418630	AT2G38650	alpha-1,4-galacturonosyltransferase	GAUT7
13345851	AT1G29510	SAUR-like auxin-respionsive protein family	SAUR68
13450075	AT3G16110	protein PDI-like 1-6	PDIL1-6
13400735	AT2G34660	ABC transporter C family member 2	MRP2
13397254	AT2G26020	PLANT DEFENSIN 1.2B	PDF1.2b
13385301	AT1G69295	plasmodesmata callose-binding protein 4	PDCB4
13369112	AT1G17370	oligouridylate binding protein 1B	UBP1B
13355470	AT1G65380	receptor-like protein CLAVATA2	CLV2

13452840	AT3G23400	Encodes FIBBRILLIN 4, fibrillins are a large family of chloroplast proteins linked with stress tolerance and disease resistance	FIB4
13402667	AT2G39140	suppressor of variegation1 causing pseudouridine synthase-like protein	SVR1
13491851	AT4G31120	protein arginine N-methyltransferase 5	SKB1
13382811	AT1G63700	YODA MAPKK kinase	YDA
13490616	AT4G27960	SUMO-conjugating enzyme UBC9	UBC9
13545285	ATMG00060	NADH dehydrogenase subunit 5	nad5
13478646	AT4G37040	methionine aminopeptidase 1D	MAP1D
13413589	AT2G27040	argonaute 4	AGO4
13452140	AT3G13090	multidrug resistance-associated protein 6	MRP6
13424730	AT3G05590	60S ribosomal protein L18-2	RPL18
13473203	AT4G25050	acyl carrier protein 4	ACP4
13416075	AT2G32930	zinc finger CCCH domain-containing protein 26	ZFN2
13543325	AT5G64290	dicarboxylate transport 2.1	DIT2.1
13487423	AT4G21100	Component of light signal transduction machinery. Involved in repression of photomorphogenesis in darkness	DDB1B
13380460	AT1G56580		
13502293	AT5G16540	Zinc finger CCCH domain-containing protein 57, DNA-binding	ZFN3
13376163	AT1G43710	putative group II plp decarboxylase	emb1075
13442096	AT3G61150	homeobox-leucine zipper protein HDG1	HDG1
13398097	AT2G28190	Superoxide dismutase [Cu-Zn]	CSD2
13354448	AT5G46290	3-oxoacyl-[acyl-carrier-protein] synthase III	KAS
13462903	AT3G61650	Tubulin gamma-1 chain	TUBG1
13539982	AT5G56250	protein hapless 8	HAP8
13498516	AT5G06870	Polygalacturonase inhibitor 2, involved in plant defense response, induced by MeJA	PGIP2
13484659	AT4G14680	3'-phosphoadenosine 5'-phosphosulfate synthase	APS3
13456801	AT3G46550	fasciclin-like arabinogalactan protein 4	SOS5
13350527	AT1G50840	polymerase gamma 2	POLGAMMA2
13510327	AT5G44130	Fasciclin-like arabinogalactan-protein 13	FLA13
13400716	AT2G34630	geranyl diphosphate synthase 1	GPS1
13441841	AT3G60620	cytidinediphosphate diacylglycerol synthase 5	CDS5
13393044	AT2G14890	arabinogalactan protein 9	AGP9
13350405	AT1G50460	hexokinase	HKL1

	13382993	AT1G64040	serine/threonine-protein phosphatase PP1 isozyme 3	TOPP3
	13517246	AT5G59920	cysteine/histidine-rich C1 domain-containing protein	ULI3
	13439579	AT3G55010	Phosphoribosylformylglycinamidine cyclo-ligase, chloroplastic	PUR5
	13433381	AT3G26060	Peroxiredoxin Q, chloroplastic	ATPRX
	13404847	AT3G59760	Cysteine synthase, mitochondrial	ATCS
	13513562	AT5G51460	Trehalose-phosphate phosphatase A	ATTPPA
	13362007	AT1G01610	glycerol-3-phosphate acyltransferase	GPAT4
	13507587	AT5G36940	Catalase-3	CAT3
	13474876	AT4G28730	glutaredoxin-C5	
	13334767	AT1G02150	pentatricopeptide repeat-containing protein	
	13504132	AT5G20935		
	13534454	AT5G42110		
	13402304	AT2G38330	MATE efflux family protein	
	13398908	AT2G30150	UDP-glucoronosyl/UDP-glucosyl transferase family protein	
	13415422	AT2G31440	gamma-secretase subunit APH1-like protein	
	13354674	AT1G63330	pentatricopeptide repeat-containing protein	
	13476805	AT4G32790	Exostosin family protein	
	13460664	AT3G56160	Sodium bile acid symporter family protein	
	13453585	AT3G25410	putative sodium-bile acid cotransporter	
	13502841	AT5G18030	SAUR-like auxin-responsive protein	
	13344574	AT1G26180		
	13337639	AT1G08845	Ribosomal L.8/L5e family protein	
	13429108	AT3G16370	GDSL esterase/lipase APG	
	13345832	AT1G29418		
	13423886	AT3G03790	ankyrin repeat and regulator of chromosome condensation (RCC1) domain-containing protein	
	13376366	AT1G44920		
	13476018	AT4G31354		
4	13398536	AT2G29290	tropine dehydrogenase	
	13369849	AT1G18810	protein phytochrome kinase substrate 3	
	13427968	AT3G14100	RNA recognition motif-containing protein	
	13455810	AT3G43610	Spc97 / Spc98 family of spindle pole body (SBP) component	
	13466659	AT4G09200	SPla/RYanodine receptor (SPRY) domain-containing protein	
	13545118	AT2G07727	cytochrome b	

13349578	AT1G48440	protein B-cell receptor-associated 31-like protein	
13545140	AT2G07835		
13478656	AT4G37080		
13500341	AT5G11450	Mog1/PsbP/DUF1795-like photosystem II reaction center PsbP family protein	PPD5
13453276	AT3G24420	hydrolase, alpha/beta fold family protein	
13540849	AT5G58260	oxidoreductase	
13338164	AT1G09900	pentatricopeptide repeat-containing protein	
13509725	AT5G42620	metalloendopeptidase	
13531184	AT5G26230		
13405024	AT2G44670		
13346942	AT1G32050	secretory carrier-associated membrane protein 4	
13444678	AT3G04760	pentatricopeptide repeat-containing protein	
13368009	AT1G14710	hydroxyproline-rich glycoprotein-like protein	
13373706	AT1G29450	SAUR-like auxin-responsive protein	
13352817	AT1G56190	phosphoglycerate kinase	
13405400	AT2G45530	RING/U-box domain-containing protein	
13531676	AT5G28050	cytidine/deoxycytidylate deaminase-like protein	
13476306	AT4G32020		
13455581	AT3G42628	phosphoenolpyruvate carboxylase-related protein	
13495244	AT4G38510	V-type proton ATPase subunit B2	
13460154	AT3G54880		
13351642	AT1G53430	Leucine-rich repeat transmembrane protein kinase	
13497887	AT5G05460	mannosyl-glycoprotein endo-beta-N-acetylglucosaminidase	
13454697	AT3G28120		
13476424	AT4G32285	putative clathrin assembly protein	
13503555	AT5G19500	Tryptophan/tyrosine permease	
13348842	AT1G44835	YbaK/aminoacyl-tRNA synthetase-associated domain-containing protein	
13476949	AT4G33080	AGC (cAMP-dependent, cGMP-dependent and protein kinase C) kinase family protein	
13437950	AT3G51150	ATP binding microtubule motor family protein	
13495904	AT4G39970	haloacid dehalogenase-like hydrolase family protein	
13389735	AT1G79720	aspartyl protease-like protein	
13464571	AT4G01883	Polyketide cyclase / dehydrase and lipid transport protein	
13440708	AT3G57810	Cysteine proteinases-like protein	

13425997	AT3G09180		
13355396	AT1G65230		
13478582	AT4G36960	RNA recognition motif-containing protein	
13516600	AT5G58250		
13361515	AT1G80380	D-glycerate 3-kinase	
13446898	AT3G09410	putative pectinacetylesterase	
13530312	AT5G24320	transducin/WD40 domain-containing protein	
13514219	AT5G52970	thylakoid lumenal protein 2	
13535223	AT5G44090	protein phosphatase 2 (formerly 2A), regulatory subunit B"	
13374544	AT1G31540	TIR-NBS-LRR class disease resistance protein	
13446161	AT3G07460		
13392616	AT2G12905		
13542220	AT5G61590	ethylene-responsive transcription factor ERF107	
13449554	AT3G14770	nodulin MtN3-like protein	
13359181	AT1G74670	putative gibberellin-regulated protein	
13402512	AT2G38780		
13486518	AT4G18440	adenylosuccinate lyase	
13400476	AT2G34170		
13426598	AT3G10760	myb family transcription factor	
13481072	AT4G02715		
13447654	AT3G10940	dual-specificity protein-like phosphatase 3	
13382220	AT1G62250		
13520171	AT5G66520	pentatricopeptide repeat-containing protein	
13446087	AT3G07300	translation initiation factor eIF-2B beta subunit	
13422185	AT2G46915		
13450493	AT3G17310	S-adenosyl-L-methionine-dependent methyltransferase-like protein	
13370697	AT1G20950	putative pyrophosphate-dependent phosphofructokinase alpha subunit	
13533250	AT5G38430	Ribulose bisphosphate carboxylase (small chain) family protein	RBCS1B
13358384	AT1G72645		
13501057	AT5G13510	Ribosomal protein L10 family protein	
13517189	AT5G59750	GTP cyclohydrolase II	
13441966	AT3G60910	S-adenosyl-L-methionine-dependent methyltransferase-like protein	
13512961	AT5G50100	putative thiol-disulfide oxidoreductase DCC	

13544348	AT5G66530	aldose 1-epimerase family protein
13528028	AT5G18760	RING/U-box domain-containing protein
13472628	AT4G23940	FtsH extracellular protease
13401358	AT2G36000	transcription termination factor domain-containing protein
13389550	AT1G79510	
13405917	AT2G46710	Rho GTPase activating protein with PAK-box/P21-Rho-binding domain
13462230	AT3G59870	
13377662	AT1G50020	
13388394	AT1G77090	PsbP domain-containing protein 4
13469756	AT4G16980	arabinogalactan family protein
13470679	AT4G19100	
13434542	AT3G29230	pentatricopeptide repeat-containing protein
13510907	AT5G45480	
13342841	AT1G21060	
13405114	AT2G44870	
13360177	AT1G77350	
13386215	AT1G72020	
13408383	AT2G07734	ribosomal protein S4
13408544	AT2G11910	
13509784	AT5G42740	glucose-6-phosphate isomerase
13347000	AT1G32220	NAD(P)-binding Rossmann-fold-containing protein
13369906	AT1G19000	myb family transcription factor
13458388	AT3G50685	
13467520	AT4G11630	ribosomal protein L19
13406959	AT2G01755	
13373751	AT1G29700	metallo-beta-lactamase domain-containing protein
13445595	AT3G06550	O-acetyltransferase family protein
13379812	AT1G55360	
13545211	AT2G07749	Mitovirus RNA-dependent RNA polymerase
13415595	AT2G31800	Integrin-linked protein kinase-like protein
13399635	AT2G32150	haloacid dehalogenase-like hydrolase domain-containing protein
13490383	AT4G27450	aluminum induced protein with YGL and LRDR motifs
13453072	AT3G24040	Core-2/I-branching beta-1,6-N-acetylglucosaminyltransferase family protein

13471363	AT4G20840	FAD-binding and BBE domain-containing protein
13355724	AT1G66180	aspartyl protease-like protein
13392212	AT2G07672	
13335374	AT1G03610	
13434746	AT3G30380	esterase/lipase domain-containing protein
13392233	AT2G07678	
13412683	AT2G25200	
13452877	AT3G23550	mate efflux domain-containing protein
13505062	AT5G23820	MD-2-related lipid recognition domain-containing protein
13336673	AT1G06640	1-aminocyclopropane-1-carboxylate oxidase-2
13465432	AT4G03635	
13536080	AT5G46160	large subunit ribosomal protein L14
13456715	AT3G46320	histone H4
13517163	AT5G59690	histone H4
13451377	AT3G19553	Amino acid permease family protein
13444562	AT3G04500	RNA recognition motif-containing protein
13480156	AT4G00620	Amino acid dehydrogenase family protein
13439087	AT3G53870	40S ribosomal protein S3-2
13544124	AT5G66120	putative 3-dehydroquinate synthase
13545128	AT2G07774	
13545190	AT2G07774	
13385322	AT1G69360	
13373203	AT1G28190	
13539841	AT5G55710	
13458629	AT3G51330	aspartyl protease family protein
13450030	AT3G15970	NUP50 protein
13353115	AT1G57720	elongation factor EF-1 gamma subunit
13364296	AT1G06690	NAD(P)-linked oxidoreductase-like protein
13401786	AT2G37050	putative receptor protein kinase
13436512	AT3G47650	DnaJ/Hsp40 cysteine-rich domain-containing protein
13535113	AT5G43870	
13477446	AT4G34290	SWIB/MDM2 domain-containing protein
13372331	AT1G25440	zinc finger protein CONSTANS-LIKE 16

13478204	AT4G35880	aspartyl protease family protein
13465848	AT4G04925	
13537702	AT5G49990	nucleobase-ascorbate transporter 5
13515291	AT5G55220	trigger factor type chaperone family protein
13493399	AT4G34220	receptor protein kinase-like protein
13496582	AT5G02450	60S ribosomal protein L36-3
13475215	AT4G29400	
13432598	AT3G23940	dihydroxy-acid dehydratase
13469510	AT4G16360	SNF1-related protein kinase regulatory subunit beta-2
13435669	AT3G44950	glycine-rich protein
13526682	AT5G15740	O-fucosyltransferase family protein
13520041	AT5G66100	winged-helix DNA-binding transcription factor family protein
13463447	AT3G62870	60S ribosomal protein L7a-2
13493773	AT4G35250	Rossmann-fold NAD(P)-binding domain-containing protein
13486584	AT4G18670	leucine-rich repeat extensin-like protein 5
13505475	AT5G25040	major facilitator protein
13465089	AT4G02790	GTP-binding protein
13443749	AT3G02700	NC domain-containing protein
13420819	AT2G43460	60S ribosomal protein L38
13466766	AT4G09630	
13453324	AT3G24480	leucine-rich repeat extensin-like protein 4
13350204	AT1G49975	
13434151	AT3G28040	probably inactive leucine-rich repeat receptor-like protein kinase
13485126	AT4G15810	GTP binding protein
13386414	AT1G72480	lung seven transmembrane receptor-like protein
13396722	AT2G24592	
13473746	AT4G26230	60S ribosomal protein L31-2
13399415	AT2G31610	40S ribosomal protein S3-1
13540270	AT5G56990	SNARE associated Golgi protein
13371404	AT1G22850	SNARE associated Golgi protein family
13468709	AT4G14950	SNARE associated Golgi protein
13428751	AT3G15640	cytochrome c oxidase subunit Vb
13527814	AT5G18380	40S ribosomal protein S16-3

13388333	AT1G76960		
13415538	AT2G31670	Stress responsive alpha-beta barrel domain protein	
13509621	AT5G42420	Nucleotide-sugar transporter family protein	
13403960	AT2G42040		
13451034	AT3G18770	Autophagy-related protein 13	
13391563	AT2G04390	40S ribosomal protein S17-1	
13539094	AT5G53580	aldo/keto reductase family protein	
13388852	AT1G78060	putative beta-D-xylosidase 7	
13493158	AT4G33760	aspartyl-tRNA synthetase	
13493685	AT4G35025		
13431605	AT3G21930	putative cysteine-rich repeat secretory protein 19	
13351900	AT1G53970		
13375475	AT1G34400		
13417793	AT2G36760	UDP-glucosyl transferase 73C	UGT73C2
13457547	AT3G48700	carboxyesterase 13	CXE13
13535432	AT5G44540	Tapetum specific protein TAP35/TAP44	
13364821	AT1G07705	CCR4-NOT transcription complex subunit 2	
13486146	AT4G17585	aluminum activated malate transporter family protein	
13449491	AT3G14510	geranylgeranyl diphosphate synthase, type II	
13375237	AT1G33607	putative defensin-like protein 26	
13545357	ATMG01030		
13342911	AT1G21245	leucine-rich repeat receptor-like protein kinase	
13373355	AT1G28550	RAB GTPase-like protein A1I	RABA1i
13425753	AT3G08520	60S ribosomal protein L41	
13463007	AT3G61826		
13483523	AT4G12220		
13334650	AT1G02074		
13395268	AT2G21010	calcium-dependent lipid-binding domain	
13446320	AT3G07850	galacturan 1,4-alpha-galacturonidase	
13453371	AT3G24516		
13458748	AT3G51642		
13545721	AT2G13422		
13397273	AT2G26120	glycine-rich protein	

13453682	AT3G25650	S-phase kinase-associated protein 1	SK15
13408378	AT2G07820		
13428988	AT3G16175	thioredoxin family protein	
13342418	AT1G19960		
13378962	AT1G53240	Malate dehydrogenase 1, mitochondrial	mMDH1
13386746	AT1G73240		
13482463	AT4G09350	chaperone DnaJ-domain containing protein	
13398023	AT2G28000	Encodes chaperonin-60 alpha, a molecular chaperone involved in Rubisco folding	CPN60A
13473175	AT4G24972	tapetum determinant 1	TPD1
13391467	AT2G04039		
13357899	AT1G71680	Lysine histidine transporter-like 5	
13338413	AT1G10522		
13451016	AT3G18750	putative serine/threonine-protein kinase WNK6	WNK6
13390161	AT1G15360	Ethylene-responsive transcription factor WIN1	WIN1
13412084	AT2G23670	homolog of Synechocystis YCF37	YCF37
13396399	AT2G23670	HOMOLOG OF SYNECHOCYSTIS YCF37	YCF37
13409748	AT2G17695		
13400845	AT2G34925	protein CLAVATA3/ESR-related 42	CLE42
13390506	AT2G01590	chlororespiratory reduction 3	CRR3
13521349	AT5G02830	pentatricopeptide repeat-containing protein	
13371792	AT1G23740	Alkenal/one oxidoreductase, helps to maintain the photosynthetic process	AOR
13406443	AT2G47844		
13545711	AT1G32290		
13429756	AT3G17840	putative inactive receptor kinase RLK902	RLK902
13475697	AT4G30825	pentatricopeptide repeat-containing protein	
13430215	AT3G18680	uridylate kinase	
13383032	AT1G64150		
13342758	AT1G20830		MCD1
13479975	AT4G00270	NA-binding storekeeper protein-related transcriptional regulator	
13417929	AT2G36880	S-adenosylmethionine synthase 3	MAT3
13434078	AT3G27830	50S ribosomal protein L12-1	RPL12-A
13531188	AT5G26330	plastocyanin-like domain-containing protein / putative mavicyanin	
13522070	AT5G04790		

13545631	AT4G19270		
13458521	AT3G51030	Thioredoxin H-type 1	TRX1
13517400	AT5G60280	concanavalin A-like lectin kinase-like protein	
13335773	AT1G04501		
13365497	AT1G09157		
13490760	AT4G28250	expansin B3	EXPB3
13525122	AT5G12190	pre-mRNA branch site protein p14	
13533451	AT5G39110	germin-like protein subfamily 1 member 14	
13545617	AT3G50250		
13395740	AT2G22055	protein RALF-like 15	RALFL15
13375245	AT1G33640		
13367065	AT1G12451		
13367387	AT1G13250	putative galacturonosyltransferase-like 3	GATL3
13406291	AT2G47500	putative kinesin heavy chain	
13500309	AT5G11380	1-deoxy-D-xylulose 5-phosphate synthase 3	DXPS3
13495716	AT4G39460	S-adenosylmethionine carrier 1	SAMC1
13521663	AT5G03760	glucomannan 4-beta-mannosyltransferase 9	ATCSLA09
13484612	AT4G14480	protein kinase family protein	
13414849	AT2G30320	putative tRNA pseudouridine synthase	
13403215	AT2G40400		
13442973	AT3G63510	FMN-linked oxidoreductase-like protein protein	
13512872	AT5G49910	HEAT SHOCK PROTEIN 70	CPHSC70-2EA
13508979	AT5G40950	50S ribosomal protein L27	RPL27
13396318	AT2G23350	poly(A) binding protein 4	PAB4
13529473	AT5G22620	phosphoglycerate/bisphosphoglycerate mutase family protein	
13377349	AT1G49140	NADH dehydrogenase [ubiquinone] iron-sulfur protein 6	
13340379	AT1G15410	aspartate-glutamate racemase-like protein	
13452760	AT3G22980	elongation factor EF-2	
13459251	AT3G52870	IQ calmodulin-binding motif family protein	
13373761	AT1G29720	Leucine-rich repeat transmembrane protein kinase	
13402227	AT2G38140	30S ribosomal protein S31	PSRP4
13458515	AT3G51010		
13521711	AT5G03850	40S ribosomal protein S28-1	

	13431526	AT3G21560	sinapate 1-glucosyltransferase	UGT84A2
	13406021	AT2G46930	pectinacetylesterase-like protein	
	13366928	AT1G12000	pyrophosphate--fructose-6-phosphate 1-phosphotransferase	
	13542051	AT5G61270	Transcription factor acting negatively in the phytochrome B signaling pathway under prolonged red light	PIF7
6	13492088	AT4G31700	Disease resistance (R) protein that specifically recognizes the hopA1 type III effector avirulence protein from Pseudomonas syringae	RPS6
	13443420	AT3G01790	large subunit ribosomal protein L13	
	13510120	AT5G43630	zinc knuckle (CCHC-type) family protein	TZP
	13426301	AT3G10060	FKBP-like peptidyl-prolyl cis-trans isomerase-like protein	
	13351979	AT1G54200		
	13437511	AT3G49670	receptor-like kinase BAM2	BAM2
	13418251	AT2G37640	expansin-A3	EXP3
	13408939	AT2G14880	SWIB/MDM2 domain-containing protein	
	13374904	AT1G32500	protein ABCI7	NAP6
	13525307	AT5G12470		
	13468629	AT4G14750	protein IQ-domain 19	IQD19
	13387187	AT1G74180	receptor like protein 14	RLP14
	13411302	AT2G21280	Rossmann-fold NAD(P)-binding domain-containing protein	SULA
	13394851	AT2G20060	large subunit ribosomal protein L4	
	13492467	AT4G32470	ubiquinol-cytochrome c reductase subunit 7	
	13457959	AT3G49670	receptor-like kinase BAM2	BAM2
	13444908	AT3G05180	GDSL esterase/lipase	
	13401303	AT2G35860	fasciclin-like arabinogalactan protein 16	FLA16
	13388299	AT1G76880	putative trihelix DNA-binding protein	
	13524742	AT5G11270	overexpressor of cationic peroxidase 3	OCP3
	13516345	AT5G57870	MIF4G domain and MA3 domain-containing protein	eIFiso4G1
	13396484	AT2G23930	small nuclear ribonucleoprotein	SNRNP-G
	13483407	AT4G11960	PGR5-like protein 1B, chloroplastic (photosystem I)	PGRL1B
	13359993	AT1G76880	putative trihelix DNA-binding protein	
	13403136	AT2G40150	trichome birefringence-like 28 protein	TBL28
	13417394	AT2G35860	fasciclin-like arabinogalactan protein 16	FLA16
	13392865	AT2G14206		

13364170	AT1G06280	LOB domain-containing protein 2	LBD2
13452831	AT3G23245		
13374616	AT1G31750	proline-rich family protein	
13410527	AT2G19900	malate dehydrogenase (oxaloacetate-decarboxylating)(NADP+)	NADP-ME1
13482473	AT4G09440		
13475053	AT4G29035	self-incompatibility S1 family protein	
13439813	AT3G55566		
13392490	AT2G10535	Encodes a member of a family of small, secreted, cysteine rich protein, involved in defense response to fungus	LCR29
13381058	AT1G59675	F-box protein	
13496416	AT5G02110	cyclin D7-1	CYCD7;1
13412370	AT2G24430	NAC domain containing protein 38	NAC038
13384593	AT1G67520	lectin protein kinase-like protein	
13384025	AT1G66570	putative sucrose transport protein SUC7	SUC7
13456472	AT3G45760	Nucleotidyltransferase family protein	
13440480	AT3G57250	Emsy N Terminus (ENT) domain-containing protein	
13436223	AT3G46760	protein kinase family protein	
13534881	AT5G43290	Probable WRKY transcription factor 49	WRKY49
13472127	AT4G22710	cytochrome P450, family 706, subfamily A, polypeptide 2	CYP706A2
13341228	AT1G17180	glutathione S-transferase TAU 25	GSTU25
13531852	AT5G28650	putative WRKY transcription factor 74	WRKY74
13454993	AT3G28990		
13507373	AT5G35715	cytochrome P450, family 71, subfamily B, polypeptide 8	CYP71B8
13436543	AT3G47720	RCD one 4-like protein	SRO4
13358274	AT1G72290	trypsin inhibitor (Kunitz) domain-containing protein	
13432860	AT3G24750		
13435258	AT3G43910		
13538481	AT5G51900	Cytochrome P450 family protein	
13487463	AT4G21216		
13456120	AT3G44710		
13531035	AT5G25870		
13376854	AT1G48145		
13498728	AT5G07310	Encodes a member of the ERF subfamily B-4 of ERF/AP2 transcription family	ERF115

13410252	AT2G19190	Encodes a receptor-like protein kinase that is involved in early defence signalling (target of WRKY6)	FRK1
13445263	AT3G05950	germin-like protein subfamily 1 member 7	
13422348	AT2G47150	Rossmann-fold NAD(P)-binding domain-containing protein	
13509745	AT5G42635	glycine-rich protein	
13439798	AT3G55515	protein rotundifolia like 7	RTFL7
13354635	AT1G63190	cystatin-related domain protein	
13541253	AT5G59110	subtilisin-like serine protease-like protein	
13455360	AT3G30778		
13427705	AT3G13540	Transcription repressor MYB5 of trichome branching	MYB5
13483883	AT4G13230	late embryogenesis abundant domain-containing protein	
13511365	AT5G46650	RING-H2 finger protein ATL30	
13338820	AT1G11450	nodulin MtN21 /EamA-like transporter protein	
13400817	AT2G34825	RALF-like 20 protein	RALFL20
13373436	AT1G28640	GDSL esterase/lipase	
13519452	AT5G64905	elicitor peptide 3 precursos, involved in defense response	PROPEP3
13467593	AT4G11760	Encodes a member of a family of small, secreted, cysteine rich protein, involved in defense response to fungus	LCR17
13409181	AT2G16120	polyol/monosaccharide transporter 1	PMT1
13495917	AT4G39986		
13414343	AT2G28850	cytochrome P450, family 710, subfamily A	CYP710A3
13414291	AT2G28755	UDP-D-glucuronate carboxy-lyase-related protein	
13400962	AT2G35208		
13393304	AT2G16019		
13458258	AT3G50373		
13384345	AT1G67160	putative F-box protein	
13475335	AT4G29770		
13482992	AT4G10870		
13407886	AT2G04870		
13423901	AT3G03800	syntaxin 1B/2/3	SYP131
13517710	AT5G60945	ECA1 gametogenesis related family protein	
13454287	AT3G27140	putative protein phosphatase 2C-like protein 45	
13383403	AT1G65130	Ubiquitin carboxyl-terminal hydrolase-related protein	

	13487188	AT4G20235	Putative cytochrome P450 71A28	CYP71A28
	13535849	AT5G45580		
	13461735	AT3G58676		
	13507130	AT5G34870	zinc knuckle (CCHC-type) family protein	
	13457884	AT3G49551		
	13391255	AT2G03600	ureide permease 3	UPS3
	13375297	AT1G33870	putative avirulence-responsive protein	
	13404822	AT2G44250		
	13510666	AT5G44940	putative F-box protein	
	13520812	AT5G01380		
7	13488057	AT4G22420	Ubiquitin-specific protease family C19-related protein	
	13373972	AT1G30060	COP1-interacting protein-like protein	
	13409491	AT2G16955		
	13391030	AT2G03010		
	13415224	AT2G31018		
	13339479	AT1G12938		
	13355843	AT1G66560	Probable WRKY transcription factor 64	WRKY64
	13456386	AT3G45490		
	13456144	AT3G44784		
	13417375	AT2G35770	serine carboxypeptidase-like 28	scpl28
	13524672	AT5G11027		
	13543843	AT5G65533		
	13481987	AT4G05523		
	13537500	AT5G49620	Member of the R2R3 factor gene family, involved in response to ABA and salt stress	MYB78
	13423348	AT3G02480	Late embryogenesis abundant protein (LEA) family protein	
	13355467	AT1G65360	Type 1 MADS-box gene that control female gametophyte development	AGL23
	13418987	AT2G39415	putative F-box protein	
	13514695	AT5G53820	Late embryogenesis abundant protein (LEA) family protein	
	13345894	AT1G29580		
	13541974	AT5G61070	Histone deacetylase 18	HDA18
	13334831	AT1G02320		
	13473661	AT4G26040		
	13461065	AT3G57160	UPF0467 protein B	

13378510	AT1G52060	jacalin-like lectin domain-containing protein	
13381126	AT1G59920	MADS-box family protein	
13347647	AT1G34041		
13372000	AT1G24200	paired amphipathic helix repeat-containing protein	
13440207	AT3G56470	F-box protein	
13482388	AT4G08967		
13460095	AT3G54770	RNA recognition motif-containing protein	
13456422	AT3G45580	RING/U-box protein with C6HC-type zinc finger	
13415757	AT2G32050		
13466845	AT4G09775		
13474983	AT4G28870		
13355841	AT1G66553		
13376746	AT1G48010	plant invertase/pectin methylesterase inhibitor domain-containing protein	
13422999	AT3G01760	Lysine histidine transporter-like 4	
13374694	AT1G31875		
13457788	AT3G49340	putative cysteine proteinase	
13518531	AT5G62780	chaperone DnaJ-domain containing protein	
13537378	AT5G49350	glycine-rich protein	
13345520	AT1G28304		
13454741	AT3G28360		PGP16
13471623	AT4G21366	protein kinase family protein	
13541350	AT5G59340	WUSCHEL-related homeobox 2	WOX2
13509102	AT5G41200	Transcription factor, MADS-box	AGL75
13391408	AT2G03937	Encodes a defens-like (DEFL) family protein	
13507394	AT5G35737		
13434283	AT3G28310		
13449173	AT3G13890	myb domain protein 26	MYB26
13534327	AT5G41720		
13338657	AT1G11125		
13530020	AT5G23780		
13419562	AT2G40790	thioredoxin-like protein CXXS2	CXXS2
13378607	AT1G52240	RHO guanyl-nucleotide exchange factor 11	ROPGEF11
13454535	AT3G27680	self-incompatibility S1 family protein	

13502063	AT5G15960	stress-induced protein KIN1 // --- // 831453 /// AT5G15960.1 // KIN1 // stress-induced protein KIN1 // --- // 831453	KIN1
13466900	AT4G09940	AIG1-like protein	
13381067	AT1G59722		
13533781	AT5G40220	Transcription factor, MADS-box	AGL43
13484419	AT4G14226		
13375477	AT1G34419		
13468052	AT4G13095	Encodes a member of a family of small, secreted, cysteine rich protein, involved in defense response to fungus	LCR37
13400355	AT2G33670	MLO-like protein 5, involved in defence response	MLO5
13441046	AT3G58780	Contains 1 MADS-box domain	SHP1
13364501	AT1G07050	CCT motif family protein	
13479091	AT4G38310	galactosyl transferase GMA12/MNN10 family protein	
13405029	AT2G44690	Rac-like GTP-binding protein ARAC9	ARAC9
13504204	AT5G21120	ETHYLENE INSENSITIVE 3-like 2 protein	EIL2
13390498	AT2G01530	MLP-like protein 329, involved in defence response	MLP329
13344833	AT1G26796	self-incompatibility protein S1-like protein	
13376257	AT1G44130	aspartyl protease-like protein	
13342376	AT1G19830	SAUR-like auxin-respionsive protein family	
13376195	AT1G43950	Putative auxin response factor 23	ARF23
13483254	AT4G11653	RALF-like 29 protein	RALFL29
13469467	AT4G16165	carbohydrate-binding X8 domain-containing protein	
13378412	AT1G51890	putative leucine-rich repeat protein kinase	
13478749	AT4G37295		
13470228	AT4G18170	Probable WRKY transcription factor 28	WRKY28
13366223	AT1G10550	xyloglucan:xyloglucosyl transferase	XTH33
13532022	AT5G33300	chromosome-associated kinesin-like protein	
13435953	AT3G45970	expansin-like A1	EXLA1
13383995	AT1G66520	methionyl-tRNA formyltransferase	pde194
13428695	AT3G15500	Encodes an ATAF-like NAC-domain transcription factor	NAC3
13483625	AT4G12500	bifunctional inhibitor/lipid-transfer protein/seed storage 2S albumin-like protein	
13535393	AT5G44430		PDF1.2c
13370081	AT1G19610		PDF1.4

13483617	AT4G12480	bifunctional inhibitor/lipid-transfer protein/seed storage 2S albumin-like protein	pEARLI
13346118	AT1G30135	protein TIFY 5A	JAZ8
13484817	AT4G14960	tubulin alpha-6 chain	TUA6
13503924	AT5G20230	blue-copper-binding protein, involved in (defense) response to wounding and fungus	BCB
13540555	AT5G57550	xyloglucan:xyloglucosyl transferase	XTH25
13414799	AT2G30250	Probable WRKY transcription factor 25	WRKY25
13393289	AT2G15960		
13527674	AT5G18020	SAUR-like auxin-responsive protein	
13449274	AT3G14090	exocyst complex component 7	EXO70D3
13405288	AT2G45250	Integral membrane protein hemolysin-III like protein	
13396584	AT2G24240	BTB/POZ domain with WD40/YVTN repeat-containing protein	
13392255	AT2G07785	NADH dehydrogenase I subunit 1	
13373702	AT1G29440	SAUR-like auxin-responsive protein	
13461382	AT3G57930		
13469187	AT4G15670	monothiol glutaredoxin-S7	
13478398	AT4G36410	Probable ubiquitin carrier protein E2 17	UBC17
13457042	AT3G47160	RING/U-box domain-containing protein	
13469199	AT4G15700	monothiol glutaredoxin-S3	
13398059	AT2G28120	major facilitator protein	
13343602	AT1G22882	Galactose-binding protein	
13468117	AT4G13340	leucine-rich repeat extensin-like protein 3	
13410034	AT2G18340	Homeobox protein 24	HB24
13389129	AT1G78830	curculin-like (mannose-binding) lectin-like protein	
13389138	AT1G78850	D-mannose binding lectin protein with Apple-like carbohydrate-binding domain	
13387939	AT1G76090	24-methylenesterol C-methyltransferase 3	SMT3
13427803	AT3G13750	beta galactosidase 1	BGAL1
13453505	AT3G24982	receptor like protein 40	RLP40
13523421	AT5G08150	suppressor of phytochrome b 5	SOB5
13366036	AT1G10140		
13336964	AT1G07420	Methylsterol monooxygenase 2-1	SMO2-1
13401770	AT2G37025	Protein TRF-like 8	TRFL8
13475813	AT4G31000	Calmodulin-binding protein	
13402751	AT2G39420	alpha/beta-hydrolase domain-containing protein	

	13370766	AT1G21130	O-methyltransferase-like protein	
	13392226	AT2G07768	Cytochrome C assembly protein	
	13538343	AT5G51550	protein EXORDIUM like 3	EXL3
	13445533	AT3G06435		
9	13533418	AT5G38900	DSBA oxidoreductase family protein	
	13375294	AT1G33850	40S ribosomal protein S15	
	13379025	AT1G53340	cysteine/histidine-rich c1 domain-containing protein	
	13518450	AT5G62623	putative defensin-like protein 264	
	13391402	AT2G03933	defensin-like protein 59	
	13383722	AT1G65890	acyl activating enzyme 12	AAE12
	13474273	AT4G27530		
	13410166	AT2G18810		
	13478104	AT4G35690		
	13492358	AT4G32208	heat shock protein 70 (Hsp 70) family protein	
	13454079	AT3G26870	self-incompatibility S1 family protein	
	13400463	AT2G34120	Cytochrome C oxidase polypeptide VIB family protein	
	13480550	AT4G01520	NAC domain containing protein 67	NAC067
10	13388429	AT1G77160		
	13356561	AT1G68290	endonuclease 2	ENDO
	13543771	AT5G65420	cyclin-D4-1	CYCD4;1
	13431841	AT3G22415		
	13544411	AT5G66607		
	13354426	AT1G62600	flavin-containing monooxygenase-like protein	
	13345840	AT1G29480		
	13455347	AT3G30730		
	13485180	AT4G15990		
	13455212	AT3G29630	UDP-glycosyltransferase-like protein	
	13466482	AT4G08876	pyrophosphate--fructose-6-phosphate 1-phosphotransferase/pyrophosphate-dependent 6-phosphofructose-1-kinase-related protein	
	13508854	AT5G40690		
	13452269	AT3G21500	1-deoxy-D-xylulose 5-phosphate synthase 1	DXPS1
	13364449	AT1G06920	ovate family protein 4	OFP4
	13360431	AT1G77950	MADS-box	AGL67

13383988	AT1G66490		
13529689	AT5G22890	C2H2 and C2HC zinc finger-containing protein	
13468107	AT4G13310	Cytochrome P450 71A20	CYP71A20
13465882	AT4G05018		
13424105	AT3G04410	no apical meristem-domain containing transcriptional regulator	
13434313	AT3G28530	UDP-glucose 4-epimerase	
13427308	AT3G12700	aspartyl protease family protein	
13409649	AT2G17470	Aluminium activated malate transporter family protein	
13433243	AT3G25760	Allene oxide cyclase 1, chloroplastic	AOC1
13417017	AT2G34930	disease resistance-like protein/LRR domain-containing protein	
13517407	AT5G60300	L-TYPE LECTIN RECEPTOR KINASE I.9, involved in Phytophthora resistance	LecRK-1.9
13443544	AT3G02140	UPF0737 protein AFP4	TMAC2
13448215	AT3G12145	polygalacturonase inhibitory protein-like protein	FLR1
13486048	AT4G17470	palmitoyl-protein thioesterase	
13396571	AT1G60140	myrcene/ocimene synthase	TPS10
13370131	AT1G19670	CORONATINE-INDUCED PROTEIN 1	CLH1
13418960	AT2G39330	Jacalin-related lectin 23	JAL23
13412050	AT2G23600	acetone-cyanohydrin lyase	ACL
13395461	AT2G21410	V-type H+-transporting ATPase subunit I	VHA-A2
13532169	AT5G35220	Peptidase M50 family protein	EGY1
13420998	AT2G43910	thiocyanate methyltransferase 1	HOL1
13351851	AT1G53885		
13351875	AT1G53885		
13413371	AT2G26740	soluble epoxide hydrolase	SEH
13491711	AT4G30740		
13445933	AT3G07090	PPPDE putative thiol peptidase family protein	
13502561	AT5G17170	rubredoxin family protein	ENH1
13359343	AT1G75210	HAD-superfamily hydrolase, subfamily IG, 5'-nucleotidase	
13480918	AT4G02420	concanavalin A-like lectin kinase-like protein	
13438719	AT3G52960	peroxiredoxin-2E	
13545583	AT4G32600	C3H4 type zinc finger protein	
13438702	AT3G52920		
13431845	AT3G22420	Serine/threonine-protein kinase WNK2	WNK2

13512541	AT5G49215	glycoside hydrolase family 28 protein	
13491011	AT4G29220		PFK1
13493379	AT4G34190	STRESS ENHANCED PROTEIN 1	SEP1
13482544	AT4G09670	uncharacterized oxidoreductase	
13441605	AT3G60130	beta glucosidase 16	BGLU16
13523358	AT5G08040	mitochondrial import receptor subunit TOM5-like protein	TOM5
13363290	AT1G04630	GRIM-19 protein	MEE4
13406739	AT2G01290	ribose-5-phosphate isomerase 2	RPI2
13522906	AT5G06860	Polygalacturonase inhibitor 1	PGIP1
13483539	AT4G12320	cytochrome P450, family 706, subfamily A, polypeptide 6	CYP706A6
13419687	AT2G41010	Calmodulin-binding protein that functions as a negative regulator of osmotic stress tolerance + regulation of SA metabolic process	CAMBP25
13476031	AT4G31390		
13336011	AT1G05200	Glutamate receptor 3.4, involved in response to wounding	GLR3.4
13397081	AT2G25605		
13413721	AT2G27290		
13358555	AT1G73060	low PSII accumulation 3 protein	LPA3
13398347	AT2G28605	PsbP domain-containing protein 2	
13517992	AT5G61670		
13338483	AT1G10660		
13469984	AT4G17560	50S ribosomal protein L19-1	
13497463	AT5G04440		
13531174	AT5G26220	ChaC-like family protein	
13376095	AT1G43560		ty2
13511468	AT5G46910	transcription factor jumonji and C5HC2 type zinc finger domain-containing protein	
13382202	AT1G62180	5'-adenylylsulfate reductase 2, chloroplastic	APR2
13528178	AT5G19260		
13537549	AT5G49740	ferric reduction oxidase 7	FRO7
13514822	AT5G54160	Encodes a flavonol 3-O-methyltransferase	OMT1
13498512	AT5G06860	Polygalacturonase inhibitor 1	PGIP1
13539269	AT5G54170	Lipid-binding START domain-containing protein	
13537205	AT5G48850	tetratricopeptide repeat domain-containing protein	ATSDI1
13503420	AT5G19290	esterase/lipase/thioesterase family protein	

13399746	AT2G32295	EXS (ERD1/XPR1/SYG1) domain-containing protein	
13485963	AT4G17230	Scarecrow-like protein 13	SCL13
13503094	AT5G18670	inactive beta-amylase 9	BMY3
13433713	AT3G26810	Protein AUXIN SIGNALING F-BOX 2	AFB2
13387919	AT1G76020	Thioredoxin superfamily protein	
13468415	AT4G14220	E3 ubiquitin-protein ligase RHF1A	RHF1A
13505503	AT5G25120	cytochrome P450 71B11	CYP71B11
13449744	AT3G15356	lectin-like protein	
13345095	AT1G27340	F-box only protein 6	
13438427	AT3G52155	Phosphoglycerate mutase family protein	
13365404	AT1G09020	sucrose nonfermenting 4-like protein	SNF4
13388867	AT1G78140		
13528330	AT5G19600	putative sulfate transporter 3.5	SULTR3;5
13545106	ATMG00110	cytochrome c biogenesis orf206	ccb206
13430236	AT3G18780	actin 2	ACT2
13399897	AT2G32540	cellulose synthase-like protein B4	CSLB04
13521038	AT5G01820	CBL-interacting serine/threonine-protein kinase 14	SR1
13425411	AT3G07350		
13386165	AT1G71810	aarF domain-containing kinase	
13389869	AT1G79920	Heat shock protein 70	
13403593	AT2G41140	CDPK-related kinase 1	CRK1
13452401	AT3G21670	major facilitator protein	
13408065	AT2G05940	Encodes a receptor-like cytoplasmic kinase that phosphorylates the host target RIN4, leading to the activation of a plant innate immune receptor RPM1	RIPK
13403439	AT2G40830	ubiquitin-protein ligase RNF115/126	RHC1A
13358502	AT1G72930	toll/interleukin-1 receptor-like protein	TIR
13418529	AT2G38300	myb-like HTH transcriptional regulator-like protein	
13541693	AT5G60460	protein transport protein SEC61 subunit beta	
13339973	AT1G14250	GDA1/CD39 nucleoside phosphatase family protein	
13460652	AT3G56110	PRA1 family protein B1	PRA1.B1
13539826	AT5G55640		
13463128	AT3G62120	prolyl-tRNA synthetase	
13417147	AT2G35240	putative plastid developmental protein DAG	

	13404563	AT2G43550	defensin-like protein 197	
	13400810	AT2G34810	FAD-binding and BBE domain-containing protein	
	13383834	AT1G66100	thionin	
	13534610	AT5G42650	Allene oxide synthase, chloroplastic	AOS
	13440117	AT3G56200	transmembrane amino acid transporter-like protein	
	13352904	AT1G56430	nicotianamine synthase	NAS4
	13449970	AT3G15900		
	13424039	AT3G04120	glyceraldehyde-3-phosphate dehydrogenase, cytosolic	GAPC1
	13374940	AT1G32640	Basic helix-loop-helix (bHLH) DNA-binding family protein	MYC2
	13482365	AT4G08870	putative arginase	
	13470305	AT4G18300	Trimeric LpxA-like enzyme	
	13433862	AT3G27325		
12	13524480	AT5G10690	pentatricopeptide repeat-containing protein	
	13508753	AT5G40500		
	13530480	AT5G24660	response to low sulfur 2	LSU2
	13403683	AT2G41310	two-component response regulator ARR8	RR3
	13445024	AT3G05520	F-actin-capping protein subunit alpha	
	13468477	AT4G14400	ankyrin repeat-containing protein	ACD6
	13456984	AT3G47010	beta-D-glucan exohydrolase - like protein	
	13504183	AT5G21100	L-ascorbate oxidase	
	13528418	AT5G19850	hydrolase, alpha/beta fold family protein	
	13539409	AT5G54610	Induced in response to Salicylic acid. Belongs to the ankyrin repeat protein family.	ANK
	13350964	AT1G51700	Dof zinc finger protein DOF1.7	DOF1
	13392313	AT2G07722		
	13362335	AT1G02340	transcription factor HFR1	HFR1
	13480813	AT4G02075	protein pitchoun 1	PIT1
	13470146	AT4G17900	PLATZ transcription factor family protein	
	13469953	AT4G17460	homeobox-leucine zipper protein HAT1	HAT1
	13419256	AT2G40000	HS1 PRO-1 2-like protein	HSPRO2
	13382362	AT1G62510	bifunctional inhibitor/lipid-transfer protein/seed storage 2S albumin-like protein	
	13388306	AT1G76930	Encodes an extensin gene, involved in response to ABA, JA, SA and wounding	EXT4
	13543253	AT5G64120	peroxidase 71	
	13539858	AT5G55790		

13	13494433	AT4G36670	putative polyol transporter 6	
	13424993	AT3G06380	tubby-like F-box protein 9	TLP9
	13454110	AT3G26910	hydroxyproline-rich glycoprotein family protein	
	13449877	AT3G15630		
	13349836	AT1G49032		
	13453844	AT3G26220	cytochrome P450 71B3	CYP71B3
	13391844	AT2G05440	glycine-rich protein 9	GRP9
	13499650	AT5G09600	succinate dehydrogenase 3-1	SDH3-1
	13471325	AT4G20670		
	13534853	AT5G43240		
	13364362	AT1G06800	phospholipase A1-Igamma1	PLA-I{gamma
	13525768	AT5G13790	Agamous-like MADS-box protein AGL15	AGL15
	13396440	AT2G23830	vesicle-associated membrane protein-like protein	
	13463327	AT3G62540	pentatricopeptide repeat-containing protein	
	13353727	AT1G60110	jacalin-like lectin domain-containing protein	
	13456089	AT3G44660	Putative histone deacetylase 10	hda10
	13346398	AT1G30640	Protein kinase family protein	
	13357031	AT1G69520	S-adenosylmethionine-dependent methyltransferase domain-containing protein	
	13478916	AT4G37770	1-aminocyclopropane-1-carboxylate synthase 8	ACS8
	13530076	AT5G23980	ferric reduction oxidase 4	FRO4
	13456858	AT3G46720	UDP-glycosyltransferase-like protein	
	13396386	AT2G23540	GDSL esterase/lipase	
	13400156	AT2G33080	Putative leucine-rich repeat disease resistance protein	RLP28
	13473409	AT4G25515	protein SEUSS-like 3	SLK3
	13384409	AT1G67290	glyoxal oxidase-related protein	
	13506522	AT5G27580	protein agamous-like 89 /	AGL89
	13410894	AT2G20465	defensin-like protein 103	
	13494609	AT4G37060	PATATIN-like protein 5	PLP5
	13354420	AT1G62580	flavin-containing monooxygenase-like protein	
	13494653	AT4G37160	protein SKU5 similar 15	sks15
	13457101	AT3G47410		
