# Supplementary material for: Brevicoryne brassicae aphids interfere with transcriptome responses of Arabidopsis thaliana to feeding by Plutella xylostella caterpillars in a density-dependent manner
Source: Oecologia. 2016 Oct 22;183(1):107–20. doi: 10.1007/s00442-016-3758-3 (PMC5239811; doi:10.1007/s00442-016-3758-3)
Supplement: Supplementary file 9 — Supplementary material 9 (PDF 3816 kb) [file 442_2016_3758_MOESM9_ESM.pdf]

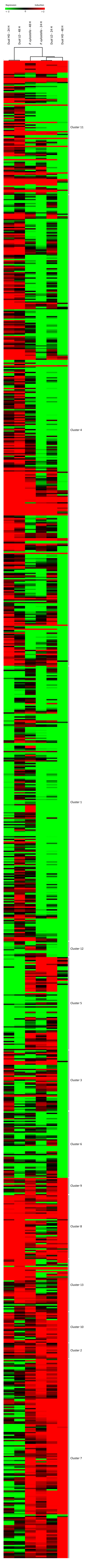
Repression
Induction
< -2
0
> 2
Dual HD - 24 H
Dual LD - 48 H
P. xylostella - 48 H
P. xylostella - 24 H
Dual LD - 24 H
Dual HD - 48 H
Cluster 11
Cluster 4
Cluster 1
Cluster 12
Cluster 5
Cluster 3
Cluster 6
Cluster 9
Cluster 8
Cluster 13
Cluster 10
Cluster 2
Cluster 7
